# Methods of Assessment of Zinc Status in Humans: An Updated Review and Meta-analysis

**DOI:** 10.1093/nutrit/nuae072

**Published:** 2024-06-25

**Authors:** Marena Ceballos-Rasgado, Anna K M Brazier, Swarnim Gupta, Victoria H Moran, Elisa Pierella, Katalin Fekete, Nicola M Lowe

**Affiliations:** Centre for Global Development, University of Central Lancashire, Preston PR1 2HE, United Kingdom; Centre for Global Development, University of Central Lancashire, Preston PR1 2HE, United Kingdom; Centre for Global Development, University of Central Lancashire, Preston PR1 2HE, United Kingdom; Centre for Global Development, University of Central Lancashire, Preston PR1 2HE, United Kingdom; Centre for Global Development, University of Central Lancashire, Preston PR1 2HE, United Kingdom; Department of Biochemistry and Medical Chemistry, University of Pécs Medical School, Pécs 7624, Hungary; Centre for Global Development, University of Central Lancashire, Preston PR1 2HE, United Kingdom

**Keywords:** zinc, zinc status, biomarker, systematic review, meta-analysis

## Abstract

**Context:**

The assessment of zinc status is difficult but essential for the identification of zinc deficiency and evaluation of interventions to improve zinc status.

**Objective:**

The purpose of this systematic review (SR) and meta-analysis was to update the previously published SR of biomarkers of zinc status, conducted by the European Micronutrient Recommendations Aligned (EURRECA) network in 2009, to answer the question: Which putative measures (biomarkers) of zinc status appropriately reflect a change in zinc intake of at least 2 weeks?

**Data Sources:**

A structured search strategy was used to identify articles published between January 2007 and September 2022 from MEDLINE (Ovid), Embase (Ovid), Cochrane Database of Systematic Reviews, and Cochrane Central Register of Controlled Trials (CENTRAL). Relevant articles were identified using previously defined eligibility criteria.

**Data Extraction:**

Data were extracted and combined with data from the previous SR.

**Data Analysis:**

A random-effects model was used to calculate pooled mean differences using STATA (StataCorp). The risk of bias and the certainty of evidence for all outcomes were assessed. Additional data on 7 of the 32 previously reported biomarkers were identified, along with data on an additional 40 putative biomarkers from studies published since 2007. Pooled data analysis confirmed that, in healthy participants, both plasma/serum zinc concentration and urinary zinc excretion responded to changes in zinc intake (plasma/serum: mean effect [95% CI], controlled studies: 2.17 µmol/L [1.73, 2.61]; P < .005, I^2^ = 97.8; before-and-after studies: 2.87 µmol/L [2.45, 3.30]; P < .005, I^2^ = 98.1%; urine zinc: 0.39 mmol/mol creatinine [0.17, 0.62]; P < .005, I^2^ = 81.2; 3.09 µmol/day [0.16, 6.02]; P = .039, I^2^ = 94.3).

**Conclusion:**

The updated analyses support the conclusion that plasma/serum and urinary zinc respond to changes in zinc intake in studies of healthy participants. Several additional putative biomarkers were identified, but more studies are needed to assess the sensitivity and reliability.

**Systematic Review Registration:**

PROSPERO no. CRD42020219843.

## INTRODUCTION

Zinc is an essential component of hundreds of enzymes, plays a pivotal role in optimal nucleic acid and protein metabolism, promotes cell growth and differentiation, and is involved in cell-mediated immunity.^1^ Consequently, zinc deficiency is associated with a range of health conditions, including, but not limited to, impaired growth and neurodevelopment in children, increased infection susceptibility in both children and adults, and adverse pregnancy-related complications.^2–5^ Assessment of zinc status is not only essential for understanding the prevalence and magnitude of zinc deficiency but also for designing, implementing, and evaluating the impact of nutritional interventions to improve zinc nutriture.^6^^,^^7^ The term “status” in this context implies a clear association between biomarker values and exposure (dose–response) and a threshold value at which clinical symptoms of deficiency can be identified, thus enabling a cutoff value or series of values for the diagnosis of “inadequate status” or “optimal status.” Unfortunately for type 2 nutrients such as zinc, this clear dose–response–clinical outcome relationship is elusive because the physiological effects of zinc deficiency result in numerous biochemical changes, linked with a broad range of physiological functions.^8^ Further complexity is added by the body's efficient regulation of zinc homeostasis that mitigates the impact of zinc intakes that are either too high or too low. When intake is insufficient, the body conserves zinc by reducing excretory losses while the fraction of dietary zinc absorbed is increased. Failure of the homeostatic response to restore zinc balance leads to clinical symptoms, such as skin lesions, and functional consequences, such as such as impaired linear and ponderal growth, and immune dysfunction.^8^ Metabolic balance studies have estimated that these changes are driven by the loss of zinc from a small, mobilizable pool of zinc representing less than 2% of total body zinc, and comprised partly of zinc located in the blood plasma, while the majority of zinc in the body located within muscle tissue, bone, and organs (2–3 g in adult males) is highly conserved and not mobilized, even in conditions of severe dietary zinc restriction. Similarly, small increases in dietary zinc can lead to a rapid repletion of the mobilizable zinc pool and improvements in the clinical and functional consequences of deficiency.^8^ Thus, identification of a sensitive and reliable biomarker has been a priority for zinc, not only to identify those with marginal deficiencies or subclinical deficiencies but also to understand the response to dietary interventions that provide moderate additional zinc intakes.^9^^,^^10^ The first step in this process is to explore the exposure–response relationship. A previous systematic review and meta-analysis conducted by the European Micronutrient Recommendations Aligned (EURRECA) network in 2009^11^ found that, of potentially 32 biomarkers, plasma zinc concentration responded in a dose-dependent manner to dietary manipulation in adult populations. Urinary and hair zinc were also found to respond reliably to changes in dietary zinc intake, but data for these were more limited. Several other potential biological indicators lacked sufficient data for evaluation.

In 2015, the Biomarkers of Nutrition for Development (BOND) Zinc Expert Panel^1^ recommended 3 measures for estimating zinc status: dietary zinc intake, plasma zinc concentration, and height-for-age in growing infants and children. It was noted, however, that plasma zinc concentration has limited responsiveness to dietary changes, considerable interindividual variability with changes in dietary zinc, and may be influenced by recent meal consumption, the time of day, inflammation, and certain drugs and hormones. Several potential or emerging zinc biomarkers were identified (eg, hair, nail, and urinary zinc concentrations; concentrations of zinc-dependent proteins; zinc kinetic markers; and DNA-repair functions), but there was insufficient evidence to recommend their use in evaluating the zinc status of individuals or populations.

A number of new studies have been published since the EURRECA systematic review and the BOND expert panel recommendations using both well-established biomarkers, such as plasma and urinary zinc concentrations, and emerging biomarkers, such as nail zinc, DNA integrity, and enzymes involved in fatty acid metabolism.^12–15^ Given these developments we have undertaken an update of the EURRECA review^11^ to include studies published from 2007 to 2023, detailing the most recent advances in zinc research and capitalizing on a more extensive dataset that includes studies for both established and putative biomarkers. By doing so, our aim was to provide a comprehensive update on the current understanding of available zinc biomarkers and to determine which biomarkers are sufficiently reliable, in terms of their response to zinc exposure, to be explored further for their potential use to evaluate zinc status in individuals and populations.

## METHODS

This systematic review was conducted according to the Preferred Reporting Items for Systematic Reviews and Meta-Analyses (PRISMA) checklist (PRISMA-2020)^16^ and registered in the International Prospective Register of Systematic Reviews (PROSPERO; registration no. CRD42020219843).

### Inclusion criteria

This review follows the same inclusion criteria as the original review,^11^ which was based on the EURRECA methodology for systematic reviews assessing potential biomarkers of micronutrient status.^17^ The inclusion criteria, based on the Population, Intervention, Comparison, Outcomes, and Study design (PICOS) elements, are presented in Table 1.

**Table 1.** PICOS Criteria for Inclusion of Studies

Parameter	Criteria
Population	Healthy humans without restriction in gender and age who had not recently used mineral or vitamin supplements
Intervention, exposures	Depletion or supplementation of zinc in humans for a span time of a period of ≥2 weeks over which the change was measured. Supplementation used the form of the following supplements: zinc sulfate, zinc acetate, zinc gluconate or Zinc methionine. In depletion studies, subjects were purposefully maintained on diets containing marginally low or deficient levels of zinc.
Comparators	Higher zinc intake vs lower or no zinc intake, or before and after zinc intake
Outcome	The outcomes of interest are those biomarkers that give us information about concentrations of zinc status in humans (eg, serum, plasma, urinary excretion, nails, hair) at baseline and at ≥2 weeks of zinc supplementation or depletion. This may include but is not restricted to the following: serum/plasma zinc urinary zinc zinc in nails and hair
Study designs	Randomized controlled trials (RCTs), controlled clinical trials, and before-and-after studies (B/A)

### Search strategy, study selection, and data extraction

The search was carried out using MEDLINE (Ovid), Embase (Ovid), Cochrane Database of Systematic Reviews, and Cochrane Central Register of Controlled Trials (CENTRAL; Cochrane Library). The search strategy was adapted from that of the original review with assistance from an expert reference librarian (C.H.) using a combination of key words and MeSH (Medical Subject Heading) terms based on the exposure of interest (terms related to zinc intake). The search strategy is presented in Information S1. The search was conducted in September 2020 and was updated in September 2021 and July 2022. The aim of this review is to update the previous EURRECA review^11^; therefore, the search was restricted to articles published from 2007. However, articles from the original review^11^ were included for completeness in the meta-analyses. The search had no language restriction. Additionally, previous reviews on biomarkers of zinc intake^1^^,^^18^ were screened to ensure no potential zinc biomarkers were overlooked.

Results from the searches were merged into EndNote X7 Referencing Software for Windows (Thomson Reuters, New York) where duplicates were removed and uploaded into Rayyan software^19^ for title and abstract screening (stage 1). The eligibility of the studies was assessed based on the inclusion criteria (A.K.M.B., M.C.-R.) (Table 1). If the abstract did not contain sufficient information for a definitive decision to be reached, a conservative approach was used, such that it was carried forward to the second (full-text) screening stage. During this first screening stage, a randomly selected 10% of articles were cross-checked by a second member of the review team (N.M.L., M.C.-R., or A.K.M.B.). At stage 2, full-text copies were obtained, and assessed based on the inclusion criteria by at least 2 members of the team (A.K.M.B., M.C.-R., E.P.). Any disagreement or uncertainty during all screening stages was discussed with members of the research team (N.M.L., V.H.M., A.K.M.B., M.C.-R., E.P., S.G.) until reaching consensus and changes were made accordingly.

### Data extraction and synthesis

Two reviewers (A.K.M.B., E.P.) extracted the data from the included articles into a specifically designed Excel (Microsoft Excel for Microsoft 365 MSO version 2208; Microsoft Corporation, Redmond, WA, USA) form. All extracted articles were cross-checked by a member of the review team (S.G., N.M.L., V.H.M., M.C.-R., A.K.M.B., E.P.). Data extracted included bibliographic information, location, study design, population characteristics (ie, sex, age), intervention (ie, type of supplement, dose, and duration), and study outcome measures, including previously identified zinc biomarkers and potential biomarkers of interest. A list of all the outcomes measured reported in the studies was reviewed by an experienced researcher (N.M.L.) and to identify plausible novel biomarkers based on a potential functional or structural role of zinc and on previous zinc biomarker reviews.^1^^,^^11^^,^^18^ Where data on potential biomarkers were presented as a graph, authors were contacted for precise data. Where data from studies could not be pooled for meta-analysis, the results are reported narratively. Data from the original 2009 review were provided by a member of the EURRECA review team (K.F.) and added to the review database.

### Risk-of-bias assessment

Risk of bias was assessed using the Cochrane Risk of Bias 2 (RoB2) tool^20^ for all randomized controlled trials (RCTs) and the Cochrane Risk of Bias in Non-Randomized Studies of Interventions (ROBINS-I) for nonrandomized trials.^21^

One reviewer (A.K.M.B.) assessed the risk of bias of the included studies. A second reviewer (V.H.M.) assessed 10% of the studies as a quality check and, where there was difference of opinion, the articles were discussed in detail and a consensus was reached.

The Grading of Recommendations Assessment, Development, and Evaluation (GRADE)^22^ assessment was used to evaluate the certainty of evidence of all outcomes included in meta-analyses. The GRADE assessment began with the assumption of high-quality evidence and was then downgraded based on risk of bias, inconsistency, indirectness, and imprecision. GRADE publication bias was only assessed if there were more than 5 articles included in the meta-analysis. The GRADE assessment was carried out by 1 reviewer (A.K.M.B.) and checked by a second reviewer (V.H.M.).

### Data preparation

Mean values and SDs of the potential biomarkers at baseline and post-intervention were extracted from each study. When IQR was reported, authors were contacted to provide values for the mean and SD. Where mean and SD values could not be obtained, studies were excluded from the meta-analyses. For those studies reporting the SE mean or 95% CI, the SD was calculated using the Cochrane RevMan Calculator (RevMan Calculator; Cochrane Training accessed in August 2023).^23^

For comparability, the units were standardized across studies as follows: plasma/serum zinc concentration units were standardized to μmol/L; urinary zinc units were standardized to either mmol/mol creatinine, µmol/day, or µmol/L; and fasting insulin units were standardized to μIU/mL (conversions were made using the online calculator https://unitslab.com; accessed March 2023^24^); fasting blood glucose units were standardized to mg/dL (conversions were made using the online calculator https://www.diabetes.co.uk/blood-sugar-converter.html, accessed July 2023^25^). Where plasma/serum zinc concentrations were corrected for inflammation, the corrected values were used in the meta-analyses.

For studies that had more than 1 intervention group with different zinc doses, both groups were included in the meta-analyses separately and data from the control group were divided into 2 to avoid double-counting as per Cochrane recommendations.^26^

### Statistical analysis

A random-effects model (DerSimonian-Laird methodology) was used to calculate the mean difference (MD) (or difference in means) of studies with similar outcomes to estimate the effect of daily zinc intake on the potential biomarkers. Since it was not possible to standardize the units for erythrocyte superoxide dismutase (SOD), the standardized MD (SMD) was used to estimate the effect of zinc supplementation. Where possible, subgroup analyses were conducted according to sex, population, dose, and supplementation type. Additionally, where possible, RCTs and uncontrolled before‐and‐after studies were analyzed independently. The before‐and‐after studies meta‐analyses also included the intervention groups of the RCTs. The calculations and the forest plots were conducted using the “METAN” command for continuous data in STATA version 16 (StataCorp, College Station, TX, USA). In all analyses, the level indicating statistical significance was set at *P* < .05.

### Usefulness of biomarker assessment

To assess the effectiveness of a biomarker reflecting a change in zinc intake, the same criteria from the original review^11^ were followed. To be considered an effective or noneffective biomarker, all criteria had to be met, as shown in Table 2.

**Table 2.** Criteria to Assess Usefulness of a Biomarker Reflecting a Change in Zinc Intake

Usefulness	Conditions
Effective biomarker	(a) Statistical difference within the forest plot (95% CI did not include 0 or *P* < .05)
	(b) ≥3 trials contributing data
	(c) ≥50 participants contributing data in the intervention arm, control, or both
Ineffective biomarker	(a) Lack of statistical difference within the forest plot (95% CI included 0 or *P* ≥ .05)
	(b) ≥3 trials contributing data
	(c) ≥50 participants contributing data in the intervention arm, control, or both
	(d) Comparable study results (ie, acceptable heterogeneity levels so that *I^2^* <50%)
Unclear evidence	Does not meet all the conditions for an effective or ineffective biomarker

### Heterogeneity and certainty assessment

Between-study heterogeneity was determined using chi-square, Cochran’s *Q* test, *I^2^* statistic,^27^ and a visual inspection of the forest plots. A chi-square *P*-value less than .1 was considered to show significant heterogeneity. Heterogeneity was rated in accordance with the Higgins et al^27^ classification approach for low (25%), moderate (50%), and high (75%) heterogeneity. The possible existence of publication bias was checked by funnel plots that were generated by plotting the effect sizes against the precision for each study. Additionally, using the “META BIAS” command, Egger’s test was also performed to evaluate possible publication bias for the analyses of the impact of zinc on the potential biomarkers where more than 10 studies were included.^28^ Visual inspection of the forest plots and Galbraith plots was used to identify potential outlier studies, which may contribute to the heterogeneity of the meta-analyses. Certainty assessment was conducted through a leave-one-out sensitivity analysis to ensure that the overall effect size was not dependent on any single study.

## RESULTS

### Description of studies

**Figure 1.**
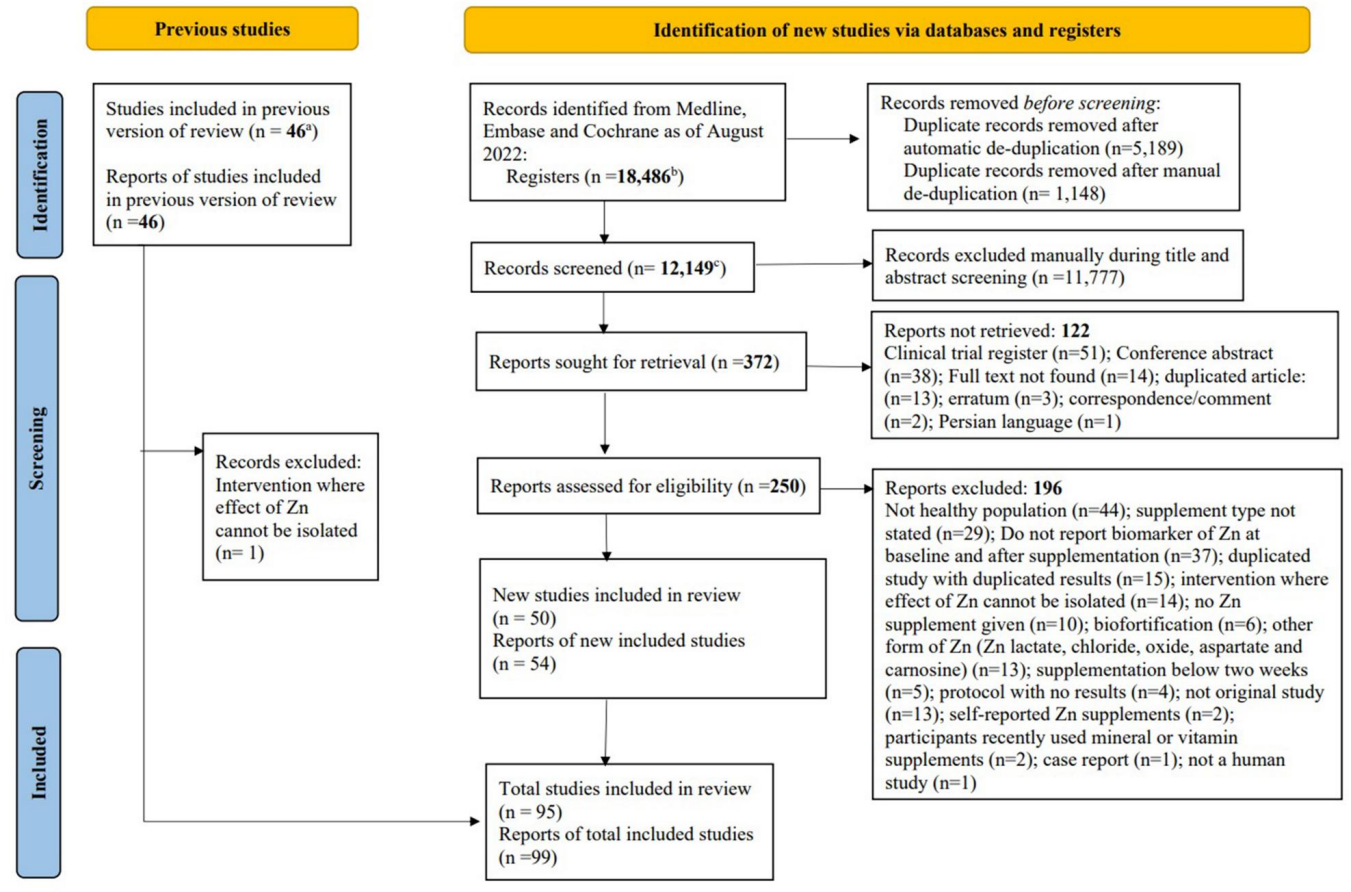
PRISMA-2020 Flow Diagram of the Search Procedure. ^a^The original review^11^ reported 48 studies in 46 articles. After reviewing articles, we noted that 2 articles^29^^,^^30^ presented data from studies already included in the review,^31^^,^^32^ resulting in a total of 46 studies in 46 articles. ^b^Of which *n* = 14 020 resulted from a search of September 2020, *n* = 2585 from a search of September 2021, and *n* = 1881 from a search of July 2022. ^c^Of which *n* = 9101 resulted from a search of September 2020, *n* = 1710 from a search of September 2021, and *n* = 1338 from a search of July 2022. *Abbreviation:* PRISMA, Preferred Reporting Items for Systematic Reviews and Meta-Analyses

The flow diagram for this review is shown in Figure 1. From the 2007–2022 search, a total of 12 149 titles and abstracts were screened; of these, 372 appeared relevant and full-text articles were sought. Of these, a total of 54 articles met the inclusion criteria and were included in the review. Some studies were reported on more than 1 article; thus, the final number of studies included from this search was 50.

From the 46 studies included in the original review,^11^ 1 study^33^ was excluded from the updated review since the effect of zinc supplementation could not be isolated from the intervention. One study^34^ from the original review^11^ was not included in the updated meta-analysis since there were no new data for erythrocyte metallothionein and the study did not report any other biomarkers for zinc. Data from all the remaining 44 articles in the original review were included in the updated meta-analyses.

A combined total of 95 studies from 99 articles were included in this review. A summary of the characteristics of the included studies is presented in Table 3.

**Table 3.** Basic Characteristics of the Included Studies in the Original and Updated Review

First author (year)	Review	**Country(s); age (y); sex; no. included**	**Description of intervention; latest time point (w); no. in intervention; no. in control group at latest time**	Micronutrient type	Study design	Biomarkers reported
Abdollahi et al (2019)^35^	U	Iran; 0.5–2; X; 682	5 mg Zn; 26; 272; 308	Zinc sulfate	RCT p	Pl Zn
Abdulla and Suck (1998)^36^	O	India and Pakistan; 37.5; X; 45	15 mg Zn; 6; 15 + 15+15	Zinc gluconate	B/A	Pl Zn
Abdulla and Svensson^a^ (1979)^37^	O	Sweden; 25; X; 12	135 mg Zn; 12; 7; 5	Zinc sulfate	nRCT c	Pl Zn; ALAD
Abdulla and Svensson^b^ (1979)^37^	O	Sweden; 25; X; 7	45 mg Zn; 12; 7	Zinc sulfate	B/A	Pl Zn; ALAD
Adriani and Wirjatmadi (2014)^38^	U	Indonesia; 4–5; X; 24	0.09 mg Zn; 26; 12; 12	Zinc sulfate	RCT p	Pl Zn1^c^; IGF-1^c^; serum retinol
Ahmadi et al (2020)^39^	U	Iran; 37.40 ± 12.26; X; 80	50 mg Zn; 8; 40; 40	Zinc sulfate	RCT p	Pl Zn
Allan et al (2000)^40^	O	United States; 27–47; M; 7	4.6 mg Zn; 10; 7	Depletion	B/A	Pl Zn, TL MT-2A mRNA
Attia et al (2022)^41^	U	Australia; 40–70; X; 98	30 mg Zn; 52; 48; 50	Zinc gluconate	RCT p	FBG, HbA1c
Ayatollahi et al (2022)^42^	U	Iran; 39.4 ± 8.7; F; 80	50 mg Zn; 13; 40; 40	Zinc sulfate	RCT p	Pl Zn
Ba Lo et al (2011)^43^	U	Senegal; 13.0 ± 2.43; X; 137	6 mg Zn; 2.14; 33; 32	Zinc sulfate	RCT p	Pl Zn
Bales et al (1994)^44^	O	United States; 59–78; X; 15	3.97 mg Zn; 2; 15; 15	Depletion	B/A	Pl Zn; Pl ALP; Pl 5′NT
Bao et al (2010)^45^	U	United States; 67 ± 7; X; 40	45 mg Zn; 26; 20; 20	Zinc gluconate	RCT p	Pl Zn^d^; IL-6; sPLA
Barrie et al (1987)^46^	O	United States; students; X; 15	50 mg Zn; 4; 15; 15	Zinc gluconate	RCT c	Pl Zn; urinary Zn; E Zn; hair Zn
Becquey et al (2016)^47^	U	United States; 0.5–2.5; X; 7641	7 mg Zn; 16, 38, 48; 90 + 86; 196	Zinc sulfate	RCT p	Pl Zn^e^
Berger et al (2015)^48^	U	United States; 9–11; F; 147	9 mg Zn; 4; 72; 75	Zinc sulfate	RCT p	Pl Zn; IGF-1
Bertinato et al (2012)^49^	U	Canada; 6–9; M; 37	5 mg Zn, 10 mg Zn, 15 mg Zn; 17.38; 10 + 9+8; 10	Zinc gluconate	RCT p	Pl Zn^f^; urinary Zinc^f^; eSOD1; CCS:SOD1
Black et al (1988)^32^	O	United States; 19–29; M; 45	50 mg Zn, 75 mg Zn; 12; 13 + 9; 9	Zinc gluconate	RCT p	Pl Zn; urinary Zn
Bogden et al (1988)^50^	O	United States; 60–89; X; 103	15 mg Zn, 100 mg Zn; 12; 36 + 31; 36	Zinc acetate	RCT p	Pl Zn, MNC Zn, Pl ALP; PMNC Zn, Plat Zn
Bogale et al (2015)^51^	U	Ethiopia; 33 ± 5; F; 48	20 mg Zn; 3.2; 24; 23	Zinc sulfate	RCT p	Pl Zn; urinary Zn; MT1; Zip 3; Zip 4; Zip 8, ZnT1^g^
Brown et al (2007)^52^	U	Peru; 0.5-0.7; X; 302	3 mg Zn; 26.1; 80; 91	Zinc sulfate	RCT p	Pl Zn
Cesur et al (2009)^53^	U	Turkey; 11 ± 3; X; 29	50 mg Zn; 8.6; 29	Zinc sulfate	B/A	Pl Zn; IGF-1; IGFBP-3
Chung et al (2008)^54^	U	United States; 19–50; M; 9	4 mg Zn; 6; 9	Depletion	B/A	Pl Zn; urinary Zn
Crouse et al (1984)^55^	O	United States; 20–55; M; 44	28.7 mg Zn; 8; 11 + 12; 10 + 11	Zinc sulfate	RCT p	Pl Zn
de Brito et al (2014)^56^	U	Brazil; 8-9; X; 30	10 mg Zn; 8.6; 15; 15	Zinc sulfate	RCT p	Pl Zn^g^
Deguchi et al (2019)^57^	U	Japan; 78.89 ± 5.75; X; 18	30 mg Zn; 12; 9	Zinc acetate	B/A	Pl Zn
Demetree et al (1980)^58^	O	United States; 27–34; M; 10	50 mg Zn; 3; 5; 5	Zinc sulfate	RCT p	Pl Zn
DiSilvestro et al (2015)^59^	U	United States; 18–24; F; 30	60 mg Zn; 6; 10; 10	Zinc gluconate	RCT p	Pl Zn; eSOD1
Donangelo et al (2002)^60^	O	United States; 20–28; F; 11	22 mg Zn; 6; 11	Zinc gluconate	B/A	Pl Zn; urinary Zn
Duchateau et al (1981)^61^	O	Belgium; 20–40 and 40–60; X; 83	150 mg Zn; 4; 20 + 20+20 + 23	Zinc sulfate	B/A	Pl Zn
Eskici et al (2017)^62^	U	Turkey; 15.1 ± 1.07; F; 20	50 mg Zn; 4; 10 + 10	Zinc sulfate	B/A	Pl Zn
Eskici et al (2016)^63^	U	Turkey; 14.2 ± 0.42; M; 10	50 mg Zn; 12; 10	Zinc sulfate	B/A	Urinary Zn
Fahmida et al (2007)^64^	U	Indonesia; 0.25–0.5; X; 800	10 mg Zn; 26; 25; 34	Zinc sulfate	RCT p	Pl Zn
Farrell et al (2011)^65^	U	United States; 18–60; X; 46	53.2 mg Zn; 2; 5	Zinc gluconate	B/A	Pl Zn
Fernandes de Oliveira et al (2009)^66^	U	Brazil; 13 ± 0.4; M; 47	22 mg Zn; 12; 21; 26	Zinc gluconate	RCT p	Pl Zn; urinary Zn; E Zn; EOF; PCD; FRAP
Field et al (1987)^67^	O	United Kingdom; 84.4; F; 15	50 mg Zn, 100 mg Zn, 150 mg Zn; 4; 5 + 5+5	Zinc sulfate	B/A	Pl Zn; MNC Zn, PMNC Zn
Fischer et al (1984)^68^	O	Canada; Adults; M; 26	50 mg Zn; 6; 13; 13	Zinc gluconate	nRCT p	Pl Zn
Freeland-Graves et al (1981)^69^	O	United States; 23–44; F; 12	3.2 mg Zn; 3; 6	Depletion	B/A	Pl Zn, mixed saliva Zn, salivary-sediment Zn
Gatto and Samman (1995)^70^	O	Australia; 24.3 ± 4.2; M; 10	50 mg Zn; 4; 10; 10	Zinc sulfate	RCT c	Pl Zn
Gomes Dantas Lopes et al (2015)^71^	U	Brazil; 8–9; X; 62	10 mg Zn; 12; 31; 31	Zinc sulfate	RCT p	Pl Zn
Grider et al (1990)^34^	O	United States; 25–32; M; 6	50 mg Zn; 9; 6	Zinc gluconate	B/A	E MT
Gülsan et al (2013)^72^	U	Turkey; 23 ± 16; X; 103	1 mg Zn; 12; 39; 40	Zinc sulfate	RCT p	Pl Zn
Gupta et al (1998)^73^	O	India; 50 ± 10.65; X; 20	150 mg Zn; 6; 20	Zinc sulfate	B/A	Pl Zn
Hayee et al (2005)^74^	O	Bangladesh; 51.62 ± 10.49; X; 20	150 mg Zn; 6; 20	Zinc sulfate	B/A	Pl Zn
Heckmann et al (2005)^75^	O	Germany; 41–82; X; 50	20 mg Zn; 12; 24; 26	Zinc gluconate	RCT p	Pl Zn, saliva Zn
Hininger-Favier et al (2007)^31^	O	France, United Kingdom, Italy; 55–85; X; 256	15 mg Zn, 30 mg Zn; 24; 126; 130	Zinc gluconate	RCT p	Pl Zn; urinary Zn; SOD1; E Zn; ALP
Hodkinson et al (2007)^29^	O	Northern Ireland; 55–70; X; 93	15 mg Zn; 25; 28 + 34; 31	Zinc gluconate	RCT p	Pl Zn^h^; urinary Zn^h^; E Zn^h^
Hollingsworth et al (1987)^76^	O	United States; 66–85; X; 8	100 mg Zn; 13; 8	Zinc sulfate	B/A	Pl Zn; L ecto-5'-NT
Hunt et al (1985)^77^	O	United States; 16; F; 138	20 mg Zn; 19; 56; 47	Zinc sulfate	RCT p	Pl Zn
Islam et al (2016)^78^	U	Bangladesh; 30–65; X; 2886	30 mg Zn; 26.1; 439 + 431; 450	Zinc sulfate	RCT p	Pl Zn^d^
Islam et al (2022)^79^	U	Bangladesh; 9.74 ± 0.84; X; 2886	10 mg Zn; 24; 52; 55	Zinc gluconate	RCT p	Pl Zn
Jafari et al (2020)^80^	U	Iran; 23.04 ± 2.97; F; 60	30 mg Zn; 12; 27; 30	Zinc gluconate	RCT p	Pl Zn; BDNF; TAC
Joray et al (2014)^81^	U	United States; 32.3 ± 1.2; F; 40	20 mg Zn; 2.42; 17; 18	Zinc sulfate	RCT p	Pl Zn^g^, DNA fragmentation
Kaseb et al (2013)^82^	U	Iran; 11.93 ± 2.3; X; 100	1 mg Zn; 16; 48; 47	Zinc sulfate	RCT p	Pl Zn
Khorsandi et al (2019)^83^	U	Iran; 18–45; X; 50	30 mg Zn; 15; 18; 22	Zinc sulfate	RCT p	Pl Zn; HOMA-IR^f^; FINS^f^
Kim et al (2014)^84^	U	South Korea; 20.8 ± 2.2; F; 40	30 mg Zn; 8; 20; 20	Zinc gluconate	RCT p	Pl Zn; urinary Zn; IL-6
Kim et al (2012)^85^	U	South Korea; 20.8 ± 2.2; F; 40	30 mg Zn; 8; 20; 20	Zinc gluconate	RCT p	Pl Zn^i^; urinary Zn^i^; FBG; HOMA-IR; Serum SOD; FINS; Pl ALP
Leite et al (2009)^86^	U	Brazil; 6–9; X; 42	5 mg Zn; 12.8; 42	Zinc sulfate	B/A	Pl Zn; total body Zn clearance; VZnTT
Lowe et al (2004)^87^	O	United States; 28 ± 6; M; 5	0.23 mg Zn; 12; 5	Depletion	B/A	Pl Zn; urinary Zn; EZP, Pl ALP, EZE, Pl Zn flux; EZP
Long et al (2022)^88^	U	United States; 0.75–0.91 X; 174	10 mg Zn; 54; 53	Zinc sulfate	RCT	Pl Zn, EZP
Lukaski et al (1984)^89^	O	United States; 32.2 ± 6.3; M; 5	3.6 mg Zn; 17; 5	Depletion	B/A	Pl Zn
Mahajan et al (1992)^90^	O	United States; 21–30; M; 8	3.2–5.6 mg Zn; 24; 8	Depletion	B/A	Pl Zn, Plat Zn, L Zn, Neutr Zn
Marques et al (2011)^91^	U	Brazil; 32 ± 8; M; 7	22 mg Zn; 8.5; 7	Zinc gluconate	B/A	Pl Zn; FBG; HOMA-IR; FINS; Pl Zn: Cu
Massih et al (2021)^92^	U	United States; 23.8 ± 1.71; M; 35	25 mg Zn; 1.8; 17 + 16	Zinc gluconate	B/A	Pl Zn; AA:DGLA ratio; GLA: LA; ARA^f^; DGLA^f^; GLA^f^; LA^f^; DGLA:LA molar ratio
Mazaheri Nia et al (2021)^93^	U	Iran; 52.68 ± 3.12; F; 116	25 mg Zn; 6; 57; 55	Zinc sulfate	RCT p	Pl Zn
Medeiros et al (1987)^30^	O	United States; 19–29; M; 31	50 mg Zn, 75 mg Zn; 12; 13 + 9; 9	Zinc gluconate	RCT p	Pl Zn^j^; urinary Zn^j^; Hair Zn^k^
Mesdaghinia et al (2019)^94^	U	Iran; 30.3 ± 5.2; F; 60	30 mg Zn; 10; 26; 26	Zinc gluconate	RCT p	Pl Zn; FBG; HOMA-IR; FINS; VLDL; GSH; TAC
Milne et al (1987)^95^	O	United States; 50-6; F; 5	2.6 mg Zn; 25; 5	Depletion	B/A	Pl Zn; urinary Zn; E Zn; Plat Zn; MNC Zn; Neutr Zn; CA; feces Zn; Pl ALP, Pl ACE
Mujica-Coopman et al (2015)^96^	U	Chile; 34.9 ± 9.5; F; 87	30 mg Zn; 12; 26; 28	Zinc sulfate	RCT p	Pl Zn
Noh et al (2014)^97^	U	South Korea; 18–28; F; 40	30 mg Zn; 8; 17; 18	Zinc gluconate	RCT p	Pl Zn^i^; urinary Zn^i^; serum SOD^i^; IL-6^i^; ZnT1; Znt2; Znt5; Znt6; Znt9
Pachotikarn et al (1985)^98^	O	United States; 18–29; M; 23	50 mg Zn; 6; 23	Zinc gluconate	B/A	Pl Zn^k^
Palin et al (1979)^99^	O	United States; 16.8 ± 5.1; X; 17	23 mg Zn; 8; 7; 10	Zinc sulfate	nRCT p	Pl Zn
Payahoo et al (2013)^100^	U	Iran; 31 ± 8; X; 60	30 mg Zn; 4; 30; 30	Zinc gluconate	RCT p	Pl Zn; FBG
Peretz et al (1993)^101^	O	Belgium; 24–46; X; 9	45 mg Zn; 8.6; 9	Zinc gluconate	B/A	Pl Zn; MNC Zn; PMNC Zn
Pinna et al (2002)^102^	O	United States; 27–47; M; 8	4.6 mg Zn; 10; 8	Depletion	B/A	Pl Zn
Prasad et al (1996)^103^	O	United States; 62 ± 7; M; 9	30 mg Zn; 26; 5	Zinc gluconate/depletion	B/A	Pl Zn; L Zn^l^; PMNC Zn^l^
Prasad et al (2007)^104^	U	United States; 55–87; X; 50	45 mg Zn; 52.14; 24; 25	Zinc gluconate	RCT p	Pl Zn, L Zn, PMNC Zn
Rohmawati et al (2021)^105^	U	Indonesia; 28.8 ± 3.6; F; 82	20 mg Zn; 12; 35; 36	Zinc sulfate	RCT p	Pl Zn
Ruz et al (1992)^106^	O	Canada; 25.3 ± 3.3; M; 15	4 mg Zn; 16; 15	Depletion	B/A	Pl Zn; urinary Zn; Neutr Zn; Neutr ALP; Neutr αDM; Plat Zn; EM Zn; EM; ALP; EM NP
Samman and Roberts (1987)^107^	O	Australia; 28; X; 47	150 mg Zn; 6; 21 + 20; 41	Zinc sulfate	RCT c	Pl Zn
Shaaban et al (2005)^108^	O	Egypt; postpartum; F; 60	10 mg Zn; 8.6; 30; 30	Zinc sulfate	RCT p	Nail Zn, hair Zn
Solati et al (2015)^109^	U	Iran; 29.77 ± 4.21; X; 50	30 mg Zn; 12; 22; 24	Zinc gluconate	RCT p	Pl Zn; BDNF
Song et al (2009)^110^	U	United States; 38 ± 8; M; 9	4 mg Zn; 6; 9	Zinc gluconate	B/A	Pl Zn^m^; eSOD1; ARA; TAC; DNA fragmentation; FRAP
Stur et al (1996)^111^	O	Austria; 71; X; 112	45 mg Zn; 104.2; 38; 42	Zinc sulfate	RCT p	Pl Zn
Sullivan and Cousins (1997)^112^	O	United States; 19–35; M; 20	50 mg Zn; 2; 10; 10	Zinc gluconate	RCT p	Pl Zn; monocyte MT cDNA
Sullivan et al (1998)^113^	O	United States; 24; M; 11	50 mg Zn; 2.5; 11	Zinc gluconate	RCT p	Pl Zn; monocyte MT cDNA; E MT
Surono et al (2014)^114^	U	Indonesia; not stated; X; 48	8 mg Zn; 12.8; 12 + 12; 12 + 12	Zinc sulfate	RCT p	Pl Zn; fecal sIgA
Swanson et al (1988)^115^	O	Switzerland; 64–95; X; 34	30 mg Zn; 4; 17; 17	zinc acetate	RCT p	Pl Zn; urinary Zn; ALP^d^, PMNC Zn, Plat Zn
Takacs et al (2020)^116^	U	Hungary; 35 ± 7; F; 22	7 mg Zn; 2; 12 + 10	Zinc acetate	B/A	Pl Zn; CVL Zn level
Tamura et al (1996)^117^	O	United States; 13–39; F; 135	25 mg Zn; 17; 70; 65	Zinc sulfate	RCT p	Pl Zn, E Zn
Tamura et al (2001)^118^	O	United States; pregnant (19 wk); F; 63	25 mg Zn; 20; 30; 31	Zinc sulfate	RCT p	Pl Zn^n^; SOD; E Zn; ALP; Pl EC-SOD
Thomas et al (1992)^119^	O	United States; 27 ± 3.6; M; 5	3.2 mg Zn; 13; 5	Depletion	B/A	Pl Zn; urinary Zn; E Zn; E MT
Vale et al (2014)^120^	U	Brazil; 6–9; X; 45	5 mg Zn; 13; 40	Zinc sulfate	B/A	Pl Zn; total body Zn clearance^f^; renal zinc clearance
Wang et al (2021)^121^	U	China; 40–58; X; 93 + 210	35 mg Zn; 2; 33; 35	Zinc gluconate	RCT p	Pl Zn^g^, DNA fragmentation
Weismann et al (1977)^122^	O	Denmark; 17–37; X; 39	135 mg Zn; 12; 13; 12	Zinc sulfate	RCT p	Pl Zn
Wessells et al (2010)^123^	U	United States; 19–54; M; 58	10 mg Zn, 20 mg Zn; 3; 10 + 20; 20	Zinc sulfate	RCT p	Pl Zn
Wessells et al (2012)^124^	U	Burkina Faso; 13.4 ± 5.1; X; 451	5 mg Zn; 3; 142 + 137; 146	Zinc sulfate	RCT p	Pl Zn
Wessells et al (2021)^13^	U	Lao People’s Democratic Republic (PDR); 1.3 ± 0.4; X; 3407	7 mg Zn; 36; 137; 138	Zinc sulfate	RCT p	Pl Zn; hair Zn; nail Zn
Yadrick et al (1989)^125^	O	United States; 25–40; F; 9	50 mg Zn; 10; 9	Zinc gluconate	B/A	Pl Zn
Yalda and Ibrahiem (2010)^126^	U	Iraq; 23.54 ± 5.719; F; 100	22.5 mg Zn; 24; 50; 50	Zinc sulfate	RCT p	Pl Zn
Yosaee et al (2020)^127^	U	Iran; 38.71 ± 7.16; X; 140	4 mg Zn; 12; 32 + 31; 33 + 29	Zinc gluconate	RCT p	Pl Zn; BDNF^e^

Abbreviations: AA:DGLA, arachidonic acid to dihomo-γ-linolenic acid ratio; ALAD, amino levunic acid dehydratase; ARA, arachidonic acid; B/A, before-and-after study; BDNF, brain-derived neurotrophic factor; CA, carbonic anhydrase; CCS:SOD1, erythrocyte CCS to SOD1 ratio; CVL Zn level, cervicovaginal lavage zinc level; DGLA:LA, dihomo-γ-linolenic acid to linoleic acid molar ratio; E, erythrocytes; E MT, erythrocyte metallothionein; EM, erythrocyte membrane; EM ALP, erythrocyte membrane phosphatase; EM NP, erythrocyte membrane neutral phosphatase; EOF, erythrocyte osmotic fragility; eSOD1, erythrocyte superoxide dismutase; EZE, endogenous zinc expression; EZP, exchangeable zinc pool; F, exclusively female group; FBG, fasting blood glucose; FINS, fasting insulin; FRAP, plasma ferric-reducing ability of plasma; GLA:LA, γ-linolenic acid to linoleic acid ratio; GSH, total glutathione; HbA1c, glycated hemoglobin; HOMA-IR, Homeostatic Model Assessment of Insulin Resistance; IGF-1, insulin-like growth factor 1; IGFBP-3, insulin-like growth factor binding protein 3; IL-6, interleukin 6; L, lymphocytes; L ecto-5'-NT, ecto-5’nucleotidase; M, exclusively male group; monocyte MT cDNA, monocyte metallothionein cDNA; MNC, mononuclear cells; MT1, gene expression of metallothionein 1; Neutr, neutrophils; Neutr Alp, neutrophil alkaline phosphatase; Neutr αDM, neutrophil α-D-mannosidase; nRCT c, non–randomized controlled trial—crossover; nRCT p, non–randomized controlled trial—parallel; O, original review^11^; PCD, plasma conjugated dienes; Pl, plasma; Pl ACE, plasma angiotensin-converting enzyme; Pl ALP, plasma alkaline phosphatase; Pl EC-SOD, plasma extracellular superoxide dismutase; Pl 5′NT, plasma 5′-nucleotidase; Pl Zn, plasma or serum zinc; Pl Zn:Cu, plasma zinc to copper ratio; Plat, platelet; PMNC, polymorphonuclear cells; RCT c, randomized controlled trial—crossover; RCT p, randomized controlled trial—parallel; sIgA, secretory immunoglobulin A; SOD, superoxide dismutase; sPLA, secretory phospholipase; TAC, total antioxidant capacity; TL MT-2A mRNA, T lymphocyte metallothionein-2A mRNA; U, updated review; VLDL, very-low-density lipoprotein; VZnTT, kinetics parameters of venous zinc tolerance test; X, mixed group; Zip3, gene expression of Zrt- and Irt-like protein 3; Zip4, gene expression of Zrt- and Irt-like protein 4; Zip8, gene expression of Zrt- and Irt-like protein 8; ZnT1, gene expression of zinc transporter 1; ZnT2, gene expression zinc transporter 2; ZnT5, gene expression zinc transporter 5; ZnT6, gene expression zinc transporter 6; ZnT9, gene expression zinc transporter 9.

a Study 1.

b Study 2.

c Excluded from meta-analysis, intake of 0.0925 mg elemental zinc, which is too low for this age group.

d Excluded from meta-analysis; unable to convert units reported.

e Excluded from meta-analysis; data presented as change.

f Excluded from meta-analysis; data unable to convert to means and SD.

g Data presented as graph.

h Not included in meta-analysis, subsample of Hininger-Favier et al.^31^

i Not included in meta-analysis as same data as Kim et al.^84^

j Excluded from meta-analysis as same data as Black et al.^32^

k Excluded from meta-analysis; no SD reported.

l Data from depletion study.

m Excluded from meta-analysis as same data as Chung et al.^54^

n Excluded from meta-analysis; same study as Tamura et al.^117^

Study participants included adults (*n* = 61),^29^^,^^30^^,^^32^^,^^34^^,^^36^^,^^37^^,^^39–42^^,^^51^^,^^54^^,^^55^^,^^58–61^^,^^65^^,^^68–70^^,^^72–75^^,^^78^^,^^80^^,^^81^^,^^83–85^^,^^87^^,^^89–98^^,^^100–102^^,^^105–107^^,^^109^^,^^110^^,^^112^^,^^113^^,^^116^^,^^119^^,^^121^^,^^122^^,^^125–127^ children and adolescents (*n* = 18),^13^^,^^35^^,^^38^^,^^43^^,^^48^^,^^49^^,^^53^^,^^56^^,^^63^^,^^66^^,^^71^^,^^72^^,^^77^^,^^82^^,^^86^^,^^114^^,^^120^^,^^124^ elderly individuals (*n* = 9),^45^^,^^50^^,^^57^^,^^67^^,^^76^^,^^103^^,^^104^^,^^111^^,^^115^ infants (*n* = 5),^47^^,^^52^^,^^64^^,^^79^^,^^88^ pregnancy and lactating women (*n* = 3),^105^^,^^117^^,^^126^ and postmenopausal women (*n* = 3).^93^^,^^95^^,^^108^ A total of *n* = 25 studies included data from females,^42^^,^^48^^,^^51^^,^^59^^,^^60^^,^^62^^,^^67^^,^^69^^,^^77^^,^^80^^,^^81^^,^^84^^,^^85^^,^^93–97^^,^^105^^,^^108^^,^^116–118^^,^^125^^,^^126^*n* = 26 studies from males,^30^^,^^32^^,^^34^^,^^40^^,^^49^^,^^54^^,^^55^^,^^58^^,^^63^^,^^66^^,^^68^^,^^70^^,^^87^^,^^89–92^^,^^98^^,^^102^^,^^103^^,^^106^^,^^110^^,^^112^^,^^113^^,^^119^^,^^123^ and *n* = 48 studies from both males and females. Studies provided zinc supplementation in the form of zinc sulfate (*n* = 49),^13^^,^^35^^,^^37–39^^,^^42^^,^^43^^,^^47^^,^^48^^,^^51–53^^,^^55^^,^^56^^,^^58^^,^^61–64^^,^^67^^,^^70–74^^,^^76–78^^,^^81–83^^,^^86^^,^^88^^,^^93^^,^^96^^,^^99^^,^^105^^,^^107^^,^^108^^,^^111^^,^^114^^,^^117^^,^^118^^,^^120^^,^^122–124^^,^^126^ zinc gluconate (*n* = 36),^29–32^^,^^34^^,^^36^^,^^41^^,^^45^^,^^46^^,^^49^^,^^59^^,^^60^^,^^65^^,^^66^^,^^68^^,^^75^^,^^79^^,^^80^^,^^84^^,^^85^^,^^91^^,^^92^^,^^94^^,^^97^^,^^98^^,^^100^^,^^101^^,^^103^^,^^104^^,^^109^^,^^110^^,^^112^^,^^113^^,^^121^^,^^125^^,^^127^ and zinc acetate (*n* = 4).^50^^,^^57^^,^^115^^,^^116^ One study provided both zinc acetate and gluconate.^116^ Supplementation ranged from 0.09 mg Zn/day to 150 mg Zn/day of elemental zinc, with a minimum duration of 1.8 weeks and a maximum duration of 104.2 weeks. Twelve studies^40^^,^^44^^,^^54^^,^^69^^,^^87^^,^^89^^,^^90^^,^^95^^,^^102^^,^^103^^,^^106^^,^^119^ presented data on the effect of depletion on potential biomarkers of zinc, and of these only 1 study^54^ was identified in the updated search.

### Quality of included studies

We were unable to obtain the full texts of 2 articles that were included in the original review,^74^^,^^90^ and although their data could be included in the meta-analyses, we were unable to conduct quality and risk-of-bias assessments on them. Overall, the Cochrane RoB2 and ROBINS-I assessment criteria found that 48% of the 96 articles were at high risk of bias. Of the 64 included RCTs, 23% were assessed to have high risk of bias, with the main contributor being the randomization process. Of the 32 nonrandomized studies of interventions (NRS), 100% of studies had a high risk of bias, with the main contributors being confounding and participant selection.^21^ For the articles included in the meta-analysis (55 RCTs and 30 NRS), the GRADE certainty of evidence assessment ranged from very low to high quality. The primary categories for downgrading the certainty of evidence were risk of bias, inconsistency, and imprecision.^22^ The risk-of-bias and GRADE assessment can be found in Information S2 and is discussed with the associated meta-analysis below.

### Biomarkers identified

The original review^11^ identified 32 potential biomarkers of zinc and new data were identified in the updated search for 6 of these (plasma/serum zinc, urinary zinc, hair zinc, nail zinc, plasma alkaline phosphatase, plasma extracellular SOD). Forty additional potential biomarkers of zinc were identified in the updated search. In total, 13 biomarkers had sufficient data (≥2 studies) to be included in a meta-analysis. A summary of these, including the number of studies, participants, and the results of the primary analyses, is presented in Table 4. For those biomarkers that did not have sufficient data to be included in a meta-analysis, a descriptive summary is presented in Information S3.

**Table 4.** Subgroup Analysis of the Results of the Meta-analysis of the Effect of Zinc Supplementation or Depletion on Potential Biomarkers of Zinc Status

Biomarker	No. of studies^a^ (no. of participants^b^)	Mean effect (95% CI)	*P*	*I^2^*, %	Appears effective as a biomarker?^c^
Serum zinc concentration (controlled trials), µmol/L	48 (4316)	2.17 (1.73, 2.61)	<.005	97.8	Yes
Serum zinc (before/after measurements), µmol/L	80 (2985)	2.87 (2.5, 3.30)	<.005	98.1	Yes
Urinary zinc, mmol/mol creatinine	4 (476)	0.39 (0.17, 0.62)	<.005	81.2	Yes
Urinary zinc, µmol/L	4 (87)	2.88 (-1.55, 7.31)	.202	95.8	Unclear
Urinary zinc, µmol/d	6 (101)	3.09 (0.16, 6.02)	.039	94.3	Yes
Alkaline phosphatase (ALP), U/L	7 (581)	3.88 (0.43, 7.33)	.028	37	No
Hair zinc, μg/g	4 (381)	7.52 (-0.94, 15.99)	.082	70.8	Unclear
Nail zinc, μg/g	2 (228)	10.47 (-12.09, 33.03)	.363	80.8	Unclear
Serum superoxide dismutase activity (SOD), U/mL	2 (92)	0.42 (-0.71, 1.55)	.465	0	Unclear
Exchangeable zinc pool (EZP), mg	2 (112)	14.44 (9.43, 19.44)	<.005	0	Unclear
Erythrocyte superoxide dismutase activity (eSOD1)^d^, U/g Hb	3 (416)	0.30 (-0.26, 0.85)	.299	80.47	Unclear
Fasting blood glucose (FBG), mg/dL	5 (226)	−0.68 (-4.56, 3.19)	.731	60.7	Unclear
Fasting insulin (FINS), μIU/mL	3 (99)	−2.02 (-3.01, -1.03)	<.005	0	Unclear
Insulin resistance (HOMA-IR)	3 (99)	−0.08 (-0.69, 0.54)	.802	78.9	Unclear
Interleukin-6 (IL-6), pg/mL	2 (115)	−0.64 (-1.18, -0.10)	.021	0	Unclear
Insulin-like growth factor 1 (IGF-1), μg/L	2 (176)	3.16 (-49.60, 55.91)	.907	36.1	Unclear
Brain-derived neurotrophic factor (BDNF), ng/mL	2 (103)	2.79 (-3.23, 8.80)	.364	89.9	Unclear
Total antioxidant capacity (TAC), μmol/L	2 (109)	116.96 (25.46, 208.45)	.012	86.6	Unclear

Abbreviation: HOMA-IR, Homeostatic Model Assessment of Insulin Resistance.

a Studies may have included >1 comparator.

b Number of participants at the end of the intervention. Participants from before-and-after observations are only considered once—that is, at the end of the intervention.

c See Table 2 for criteria to assess usefulness of a biomarker reflecting a change in zinc intake.

d Values from standardized mean difference.

#### Plasma/serum zinc concentration

The most frequently investigated biomarker of zinc status was plasma/serum zinc concentration. Of the 95 articles that included data on plasma/serum zinc concentration, 52 were found in the updated search and 43 were identified in the original review. Data from 14 articles^29^^,^^30^^,^^38^^,^^45^^,^^47^^,^^49^^,^^56^^,^^78^^,^^81^^,^^85^^,^^97^^,^^110^^,^^118^^,^^121^ could not be included in the meta-analyses and reasons for exclusion are given in Table 2. Thus, a total of 82 studies (from 81 articles) were included in the meta-analyses, 41 from the original review and 41 from the updated review. Forty-six studies were parallel controlled trials,^13^^,^^31^^,^^32^^,^^35^^,^^39^^,^^42^^,^^43^^,^^48^^,^^50–52^^,^^55^^,^^58^^,^^59^^,^^64^^,^^66^^,^^68^^,^^71^^,^^72^^,^^75^^,^^77^^,^^79^^,^^80^^,^^82–84^^,^^88^^,^^93^^,^^94^^,^^96^^,^^99^^,^^100^^,^^104^^,^^105^^,^^109^^,^^111–115^^,^^117^^,^^122–124^^,^^126^^,^^127^ 4 were crossover parallel trials,^37^^,^^46^^,^^70^^,^^107^ and 32 were before-and-after studies.^36^^,^^37^^,^^40^^,^^44^^,^^53^^,^^54^^,^^57^^,^^60–62^^,^^65^^,^^67^^,^^69^^,^^73^^,^^74^^,^^76^^,^^86^^,^^87^^,^^89–92^^,^^95^^,^^98^^,^^101–103^^,^^106^^,^^116^^,^^119^^,^^120^^,^^125^

##### Analysis of controlled trials

**Figure 2.**
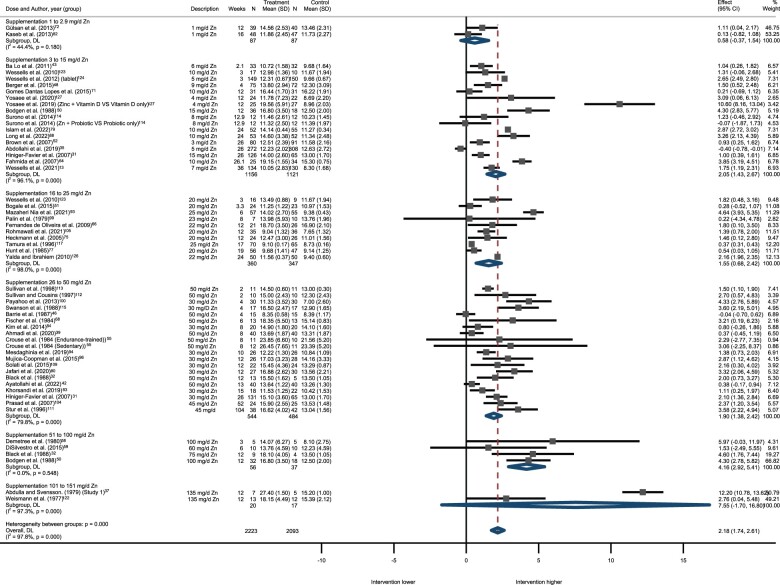
Forest Plot of Controlled Trials Assessing Effect of Zinc Supplementation on Serum Zinc, Subgroup Analysis by Dose. The figure indicates the degree of heterogeneity between the studies, *I^2^*, and the significance of this heterogeneity indicated by the *P*-value; the effect size with 95% CI and the overall effect estimate (DL) are shown. *Abbreviation:* DL, diamond line

Pooled data from the controlled trial studies, including 4316 participants, revealed an overall significant effect of zinc intake on plasma/serum zinc concentration (MD: 2.17 µmol/L; 95% CI: 1.73–2.61), yet with high heterogeneity between studies (*I^2^ *=* *98%) (Table 4). Subgroup analyses were performed by population, sex, serum/plasma zinc concentration at baseline, supplementation dose, and study design (RCTs vs non-RCTs). A summary of the subgroup analyses is presented in Table 5. A forest plot of the effect of zinc supplementation on plasma/serum zinc in the controlled trial studies by dose is presented in Figure 2.

**Table 5.** Subgroup Analysis of the Results of the Meta-analysis of the Effect of Zinc Supplementation or Depletion on Plasma/Serum Zinc Concentration (µmol/L) Including Data of Controlled Trials and Before-and-After Studies

Analysis	Controlled trials				Before-and-after^a^			
	No. of studies^b^ (no. of participants^c^)	Mean effect (95% CI)	*I^2^*, %	Useful biomarker?^d^	No. of studies^b^ (no. of participants^c^)	Mean effect (95% CI)	*I^2^*, %	Useful biomarker?^d^
All studies	48 (4316)	2.17 (1.73, 2.61)	97	Yes	80 (2985)	2.87 (2.45, 3.30)	98.1	Yes
Infants	4 (442)	2.71 (1.68, 3.75)	92.4	Yes	4 (210)	3.18 (1.55, 4.81)	94.6	Yes
Children and adolescents	11 (1789)	0.96 (0.07, 1.86)	96.2	Yes	14 (1127)	2.24 (1.38, 3.09)	97.7	Yes
Pregnancy and lactation	3 (306)	1.30 (-0.09, 2.70)	99.4	Unclear	3 (155)	0.83 (-0.86, 2.51)	99.6	Unclear
Adults	22 (996)	2.65 (1.80, 3.50)	92.9	Yes	46 (865)	3.28 (2.62, 3.94)	94.6	Yes
Postmenopausal women	1 (112)	4.64 (3.93, 5.35)	NA	Unclear	1 (57)	5.12 (4.42, 5.82)	NA	Unclear
Elderly	4 (267)	2.20 (1.74, 2.66)	30.9	Yes	9 (184)	3.23 (2.31, 4.16)	58.4	Yes
Males	8 (252)	1.67 (1.34, 2.01)	0	Yes	22 (306)	2.59 (1.85, 3.33)	91.9	Yes
Females	13 (1018)	1.58 (0.86, 2.29)	97.5	Yes	22 (664)	2.83 (2.05, 3.60)	98	Yes
Mixed	27 (3046)	2.39 (1.84, 2.94)	96	Yes	38 (1912)	2.96 (2.39, 3.54)	96	Yes
Low plasma/serum zinc concentration at baseline^e^	4 (502)	2.46 (0.90, 4.01)	89	Yes	4 (247)	2.57 (0.89, 4.26)	92.9	Yes
Normal plasma/serum zinc concentration at baseline^f^	44 (3844)	2.14 (1.69, 2.60)	97.9	Yes	76 (2635)	2.89 (2.45, 3.33)	98.1	Yes
Supplement formula: zinc sulfate	29 (3081)	1.96 (1.38, 2.54)	98.6	Yes	40 (1972)	3.22 (2.59, 3.85)	98.8	Yes
Supplement formula: zinc gluconate	18 (1097)	2.17 (1.55, 2.80)	84.3	Yes	24 (706)	2.56 (1.94, 3.18)	91.6	Yes
Supplement formula: zinc acetate	2 (138)	2.15 (1.71, 2.60)	0	Unclear	3 (94)	3.60 (2.87, 4.33)	0	Unclear
Dose								
Depletion <3 mg/day Zn	—	—	—	—	2 (10)	3.85 (-5.65, 13.36)	98.4	Unclear
Depletion 3 to 5 mg/day Zn	—	—	—	—	9 (78)	1.43 (0.27, 2.58)	87.8	Yes
Supplementation 1 to 2.9 mg/day Zn	2 (174)	0.58 (-0.37, 1.54)	44.4	Unclear	2 (87)	1.05 (0.31, 1.79)	0	Unclear
Supplementation 3 to 15 mg/day Zn	15 (2277)	2.05 (1.43, 2.67)	96.1	Yes	19 (1384)	2.21 (1.59, 2.83)	96	Yes
Supplementation 16 to 25 mg/day Zn	10 (707)	1.55 (0.68, 2.42)	98	Yes	13 (411)	1.75 (0.92, 2.57)	98.7	Yes
Supplementation 26 to 50 mg/day Zn	19 (1028)	1.90 (1.38, 2.42)	79.8	Yes	28 (662)	3.23 (2.43, 4.02)	92.2	Yes
Supplementation 51 to 100 mg/day Zn	4 (93)	4.16 (2.92, 5.41)	0	Yes	8 (84)	5.19 (1.81, 8.58)	91.9	Yes
Supplementation 101 to 151 mg/day Zn	2 (37)	7.55 (-1.70, 16.80)	97.3	Unclear	10 (166)	2.85 (2.43, 3.28)	94.2	Yes
RCTs	45 (4261)	1.97 (1.54, 2.39)	97.6	Yes	—	—	—	—
Non-RCTs	3 (55)	5.41 (-2.42, 13.23)	95.7	Unclear	—	—	—	—

Abbreviations: NA, not available; RCT, randomized controlled trial.

a In addition to before-and-after studies, analyses include before-and-after data from intervention arms included in controlled trials.

b Studies may have included >1 comparator.

c Number of participants at the end of the intervention. Participants from before-and-after observations are only considered once—that is, at the end of the intervention.

d See Table 2 for criteria to assess usefulness of a biomarker reflecting a change in zinc intake.

e Considered low serum zinc if serum zinc <8.7 µmol/L in children <10 years considered, <9.1 µmol/L in women, and <9.3 µmol/L in men.^6^

f Considered normal serum zinc if serum zinc ≥8.7 µmol/L in children ≥10 years considered, ≥9.1 µmol/L in women, and ≥9.3 µmol/L in men.^6^

As shown in Table 5, overall subgroup analyses of the controlled trials showed that zinc supplementation had a significant effect on plasma/serum zinc concentration in infants, children and adolescents, adults, elderly individuals, and in populations with a low or normal zinc status at baseline. Most had high levels of heterogeneity (*I^2^* ≥75%), except for elderly (*I^2^* =30%) and male (*I^2^* = 0%) populations where heterogeneity was moderate and low, respectively. Pooled data from 3 studies of pregnant and lactating women^105^^,^^117^^,^^126^ showed no significant effect of zinc supplementation (MD: 1.30; 95% CI: –0.09, 2.70; *I^2^* = 99.5%). Only 1 study^93^ reported data from postmenopausal women, but the effect of zinc supplementation on plasma/serum zinc was significant (MD: 4.64; 95% CI: 3.93–5.35; *I^2^* = not available).

Similarly, subgroup analysis by dose in the form of zinc sulfate or zinc gluconate, and in amounts of 3–100 mg/day of elemental zinc, revealed a significant effect on plasma/serum zinc, but with high heterogeneity (*I^2^* ≥75%). For studies providing zinc acetate, at doses of less than 3 mg/day and 101–152 mg/day of elemental zinc, the overall effect remains unclear as fewer than 3 trials contributed to the subgroup analyses. Finally, pooled data from RCTs only showed a significant effect of zinc supplementation on plasma/serum zinc, but heterogeneity remained high (MD: 1.97; 95% CI: 1.54–2.39; *I^2^* =97.6%). Data from the 3 non-RCTs^37^^,^^68^^,^^99^ showed no significant effect of zinc supplementation on plasma/serum zinc, with high heterogeneity between studies (MD: 5.41; 95% CI: -2.42, 13.23; *I^2^* = 95.7%).

#### Analysis of before-and-after data

Eighty studies were included in the meta-analyses, including before-and-after studies and controlled trials for which baseline data were available. A summary of the subgroup analyses is presented in Table 5.

**Figure 3.**
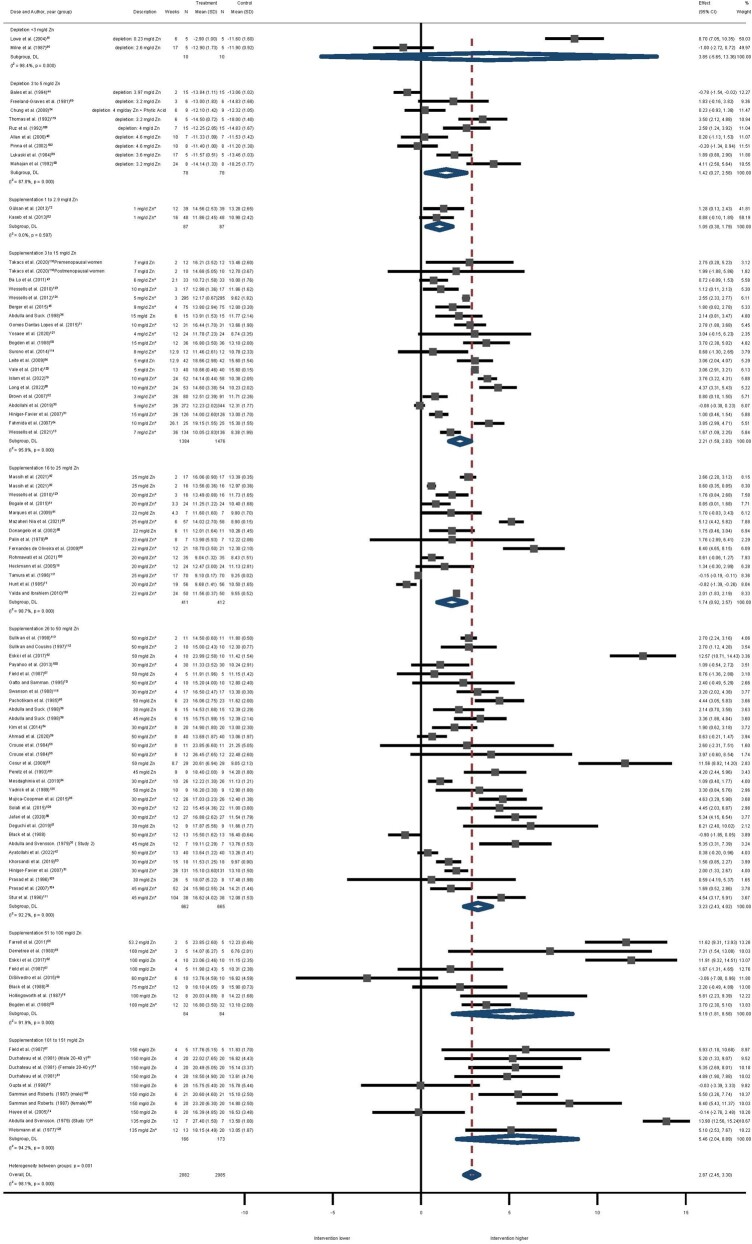
Forest Plot of the Effect of Zinc Supplementation/Depletion in Serum/Plasma Zinc in Before-and-After Data by Dose. The figure indicates the degree of heterogeneity between the studies, *I^2^*, and the significance of this heterogeneity indicated by the *P*-value; the effect size with 95% CI and the overall effect estimate (DL) are shown. *Before and after data from RCT studies. *Abbreviation:* DL, diamond line

The results of the analyses were consistent with those of the controlled trials. For the depletion studies, only before-and-after data were available. Two studies^87^^,^^95^ provided total zinc intakes of less than 3 mg Zn/day and 9 studies^40^^,^^44^^,^^54^^,^^69^^,^^89^^,^^90^^,^^102^^,^^106^^,^^119^ provided total zinc intakes of 3 to 5 mg Zn/day. Intakes of less than 3 mg Zn/day were not significant (MD: 3.85; 95% CI: –5.65, 13.36; *I^2^* =98.4%), whereas depletion intakes of 3 to 5 mg zinc per day were statistically significant (MD: 1.43; 95% CI: 0.27–2.58; *I^2^* =87.8%). However, heterogeneity between studies remained high. Figure 3 presents a forest plot of the effect of zinc supplementation/depletion in serum/plasma zinc in before-and-after data by dose.

Following visual inspection of the forest plots and Galbraith plots we completed an exercise of leave-one-out sensitivity analysis. After completing this exercise, heterogeneity between the studies remained high (*I^2^* ≥75%) and there was no impact on the overall effect; therefore, all studies were retained. The GRADE quality-of-evidence assessment for serum/plasma zinc ranged from very low to high (Information S2).

#### Urinary zinc excretion

A total of 20 articles reported urinary zinc data.^29–32^^,^^46^^,^^49^^,^^51^^,^^54^^,^^60^^,^^63^^,^^66^^,^^84^^,^^85^^,^^87^^,^^95^^,^^97^^,^^98^^,^^106^^,^^115^^,^^119^ Controlled trials and before-and-after trials were combined for meta-analyses as there were insufficient studies to warrant separate analyses. Data from 6 articles^29^^,^^30^^,^^49^^,^^85^^,^^97^^,^^98^ were not included in the meta-analyses. Studies that were excluded and reasons for exclusion are presented in Table 3.

A total of 14 studies were included in the meta-analyses, 9 studies^31^^,^^32^^,^^46^^,^^60^^,^^87^^,^^95^^,^^106^^,^^115^^,^^119^ from the original review^11^ and 5 studies^51^^,^^54^^,^^63^^,^^66^^,^^84^ from the updated search. Six studies^54^^,^^84^^,^^87^^,^^95^^,^^115^^,^^119^ reported urinary zinc measured as µmol/day, 4 studies^31^^,^^32^^,^^60^^,^^66^ as mmol/mol creatinine, and 4 studies^46^^,^^51^^,^^63^^,^^106^ as µmol/L. The results of the analyses for each unit reported are presented in Table 4, and the meta-analyses of zinc supplementation/depletion on urinary zinc (µmol/d, mmol/mol creatinine, and µmol/L) by subgroup are presented in Table 6.

**Table 6.** Summary of the Subgroup Analysis of the Results of the Meta-analysis of the Effect of Zinc Supplementation or Depletion on Urinary Zinc

Analysis	No. of studies^a^ (no. of participants^b^)	Mean effect (95% CI), µmol/day	*I^2^*, %	No. of studies^a^ (no. of participants^b^)	Mean effect (95% CI), mmol/creatinine	*I^2^* ^,^ %	No. of studies^a^ (no. of participants^b^)	Mean effect (95% CI), µmol/L	*I^2^* ^,^ %
All studies	6 (101)	3.09 (0.16, 6.02)	94.3	4 (476)	0.39 (0.17, 0.62)	81.2	4 (87)	2.88 (-1.55, 7.31)	95.8
Infants	—	—	—	—	—	—	—	—	—
Children and adolescents	—	—	—	1 (47)	0.77 (0.56, 0.98)	NA	1 (10)	7.87 (6.79, 8.96)	NA
Pregnancy and lactation	—	—	—	—	—	—	—	—	—
Adults	4 (69)	2.50 (-1.01, 6.00)	94.9	5 (429)	0.25 (0.13, 0.37)	26.5	3 (77)	1.28 (0.16, 2.39)	0
Postmenopausal women	—	—	—	—	—	—	—	—	—
Elderly	1 (27)	9.30 (5.98, 12.62)	NA	—	—	—	—	—	—
Males	4 (40)	3.87 (0.25, 7.49)	94.3	2 (78)	0.71 (0.53, 0.89)	0	1 (14)	−1.60 (-9.29, 6.09)	NA
Females	3 (61)	2.99 (-0.70, 6.67)	78.1	1 (11)	0.27 (0.02, 0.52)	NA	2 (58)	4.38 (-2.49, 11.25)	98.4
Mixed	—	—	—	1 (387)	0.21 (0.03, 0.40)	68.3	1 (15)	2.29 (0.35, 4.23)	NA
Supplement formula: zinc sulfate	1 (5)	−0.30 (-2.11, 1.51)	NA	—	—	—	2 (58)	4.38 (-2.49, 11.25)	98.4
Supplement formula: zinc gluconate	1 (40)	1.42 (-1.44, 4.28)	NA	—	—	—	1 (15)	2.29 (0.35, 4.23)	NA
Supplement formula: zinc acetate	1 (27)	9.30 (5.98, 12.62)	NA	—	—	—	—	—	—
Depletion <5 mg Zn/day	4 (29)	2.98 (-0.48, 6.43)	92.1	—	—	—	1 (14)	1.60 (-9.29, 6.09)	NA
Supplementation 15 to 25 mg/day Zn	1 (5)	−0.30 (-2.11, 1.51)	NA	3 (249)	0.38 (-0.03, 0.79)	92.2	1 (48)	0.86 (-0.52, 2.24)	NA
Supplementation 26 to 50 mg/day Zn	2 (67)	5.31 (-2.41, 13.04)	92	2 (214)	0.32 (0.18, 0.47)	0	2 (25)	5.14 (-0.33, 10.61)	95.9
Supplementation 51 to 100 mg/day Zn	—	—	—	1 (13)	0.59 (-0.04, 1.22)	NA	—	—	—
Supplementation 101 to 151 mg/day Zn	—	—	—	—	—	—	—	—	—

Abbreviation: NA, not available.

a Studies may have included >1 comparator.

b Number of participants at the end of the intervention. Participants from before-and-after observations are only considered once—that is, at the end of the intervention.

Zinc supplementation/depletion had a significant effect on urinary zinc measured as µmol/day (MD: 3.09; 95% CI: 0.16–6.02; *I^2^ *=* *94.3%) (Table 4), and can be considered an effective biomarker of zinc intake according to the previously described criteria (Table 2). However, heterogeneity was high, and data were only available for adults and in 1 study of elderly populations.^115^

The subgroup analysis by sex showed a significant effect of zinc supplementation/depletion on males but not on females. Three studies^54^^,^^87^^,^^119^ reported the effect of depletion on urinary zinc (µmol/day), and 3 studies reported the effect of zinc supplementation on urinary zinc (µmol/day). In 1 study, zinc supplementation was provided in the form of zinc sulfate,^119^ 1 study in the form of zinc gluconate,^84^ and 1 study provided zinc supplementation in the form of zinc acetate.^115^ Results from the depletion studies showed a significant effect of depletion on urinary zinc (µmol/day), yet heterogeneity was high (MD: 2.98; 95% CI: –0.48, 6.43; *I^2^ *=* *92.1%).

Zinc supplementation (given as zinc gluconate) had a significant effect on urinary zinc measured as mmol/mol creatinine (Table 4) (MD: 0.39; 95% CI: 0.17–0.62; *I^2^ *=* *81.2%) and can be considered an effective biomarker of zinc intake according to the previously described criteria (Table 2). Of these studies, only 1 study^66^ included children and adolescents. As shown in Table 5, results of the subgroup analysis on the effect of zinc supplementation in adults showed a significant effect of zinc supplementation, with low heterogeneity between studies (MD: 0.25; 95% CI: 0.13–0.37; *I^2^* = 26.5%).

Four studies measured the effect of zinc supplementation/depletion on urinary zinc measured as µmol/L.^46^^,^^51^^,^^63^^,^^106^ Analysis of the pooled data did not reveal a significant effect of zinc intake on urinary zinc, with high heterogeneity of the data between studies (Table 4). From the studies measuring urinary zinc as µmol/L, 1 study assessed the effect of depletion on urinary zinc,^106^ 1 study provided supplementation in the form of zinc gluconate,^46^ and 2 studies provided supplementation in the form of zinc sulfate.^51^^,^^63^ Only 1 study^63^ was in children and adolescents and 3 studies were conducted in adults.^46^^,^^51^^,^^106^ As shown in Table 5, the subgroup analysis in the adult population was statistically significant, without significant heterogeneity (*I^2^* = 0%). The GRADE quality-of-evidence assessment for urinary zinc ranged from very low to high (Information S2).

#### Alkaline phosphatase

A total of 7 studies^31^^,^^44^^,^^50^^,^^85^^,^^87^^,^^95^^,^^118^ reported data on alkaline phosphatase, of which 1 study was identified in the updated search.^85^ Analysis of the pooled data suggests that alkaline phosphatase is not an effective biomarker of zinc intake (MD: 3.88; 95% CI: 0.43–7.33; *I^2^ *=* *37%). Subgroup analyses showed no significant effect of zinc intake on alkaline phosphatase activity when stratified by sex, dosage, or micronutrient type (Information S2). The GRADE quality-of-evidence assessment for alkaline phosphatase ranged from very low to low (Information S2).

#### Hair and nail zinc concentration

Of the 4 studies included in the original review, 3 studies^30^^,^^46^^,^^108^ assessed hair zinc and 1 study^108^ assessed nail zinc. One additional study was retrieved in the updated search^13^ that reported both hair and nail zinc data, expressed as geometric means. Because the author was able to provide arithmetic mean values the data were combined with that of the original review. Pooled analysis of hair zinc concentration resulted in a significant mean effect of 7.52 µg/g (95% CI: –0.94, 15.99; *I^2^ *=* *71%, *P* = .016) and pooled analysis of nail zinc concentration resulted in a significant effect 10.47 µg/g (95% CI: –12.09, 33.03; *I^2^ *=* *80.8%; *P *= .023) (Table 4). However, neither hair nor nail zinc concentration met the criteria for an effective biomarker (Table 2). The GRADE quality-of-evidence assessment for both hair and nail zinc was very low (Information S2).

#### Serum and erythrocyte SOD

Three articles reported serum SOD,^85^^,^^97^^,^^118^ but 1 article^97^ was excluded from the meta-analysis as it repeated data reported by Kim and Lee.^85^ Of the remaining 2 articles, 1 article was conducted in an adult population^85^ and 1 with pregnant women.^118^ Analysis of the pooled data revealed that zinc supplementation did not have a significant effect on serum SOD, and the effectiveness of serum SOD as a biomarker of zinc status remains unclear given the lack of trials contributing data (Table 4).

Three studies reported the effect of zinc supplementation on erythrocyte SOD.^31^^,^^59^^,^^110^ Since it was not possible to combine units reported (U/mg hemoglobin [Hb], U/mL packed cells, U/g Hb), effect measures were calculated as SMDs. As shown in Table 4, zinc supplementation did not appear to have a significant effect on erythrocyte SOD, yet the effectiveness of erythrocyte SOD as a biomarker remains unclear, as heterogeneity between studies was high. The GRADE quality-of-evidence assessment for both serum and erythrocyte SOD was very low (Information S2).

#### Fasting blood glucose

**Figure 4.**
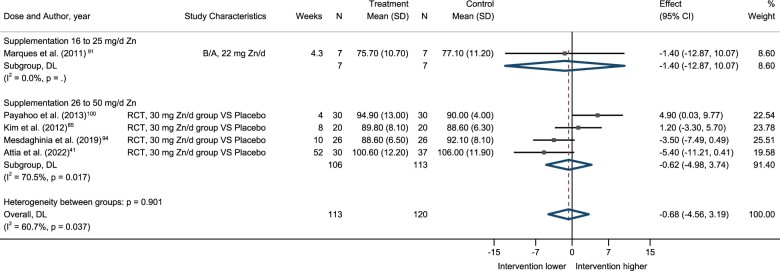
Forest Plot of the Effect of Zinc Supplementation in Fasting Glucose (mg/dL) by Dose. The figure indicates the degree of heterogeneity between the studies, *I*^2^, and the significance of this heterogeneity indicated by the *P*-value; the effect size with 95% CI and the overall effect estimate (DL) are shown. *Abbreviations:* B/A, before-and-after study; DL, diamond line; RCT, randomized controlled trial

Five studies^41^^,^^85^^,^^91^^,^^94^^,^^100^ included data on the effect of zinc supplementation on fasting blood glucose (mg/dL). All 5 studies administered zinc doses between 22 and 30 mg Zn/day for a period of 4 to 52 weeks in the form of zinc gluconate. As shown in Table 4, zinc supplementation did not have a significant effect on fasting blood glucose. However, it is interesting to note that subgroup analysis by dose (Figure 4) revealed a trend towards a reduction in fasting blood glucose with duration of the intervention. The GRADE quality-of-evidence assessment for fasting glucose was very low (Information S2).

#### Fasting insulin

Four studies^83^^,^^85^^,^^91^^,^^94^ reported fasting insulin levels (μIU/mL). Data from 1 study^83^ were not included in meta-analysis as they were presented as geometric means. All included studies were conducted in adults. As shown in Table 4, the meta-analysis suggests a significant effect of zinc supplementation on fasting insulin (–2.02; –3.01, –1.02; *I^2^ *=* *0%), with minimal heterogeneity. However, caution is needed when interpreting these results due to the small sample size and high publication bias. The GRADE quality-of-evidence assessment for fasting insulin was very low (Information S2).

#### Insulin resistance (Homeostatic Model Assessment of Insulin Resistance)

Four studies^83^^,^^85^^,^^91^^,^^94^ included data on insulin resistance. One study was not included in the meta-analysis because the data were presented as geometric means.^83^ As shown in Table 4, meta-analysis of the included studies suggested that zinc supplementation did not have a significant effect on insulin resistance, and heterogeneity between studies was high. The GRADE quality-of-evidence assessment for insulin resistance (Homeostatic Model Assessment of Insulin Resistance [HOMA-IR]) was very low (Information S2).

#### Interleukin-6

Three articles^45^^,^^84^^,^^97^ that measured the effect of zinc supplementation on interleukin-6 (IL-6) were identified. Data from 1 article^97^ were excluded as they duplicated data reported by Kim and Ahn.^84^ As shown in Table 4, it remains unclear if IL-6 is a potential biomarker of zinc intake. Although there was a statistically significant impact of zinc supplementation on IL-6 (MD: –0.64; 95% CI: –1.18, –0.10; *I^2^ *=* *0%), there was a lack of available studies. The GRADE quality-of-evidence assessment for IL-6 was low (Information S2).

#### Insulin-like growth factor 1

Three studies reported the effect of zinc supplementation on insulin-like growth factor 1 (IGF-1).^38^^,^^48^^,^^53^ Data from 1 study^38^ were excluded from the meta-analysis since the intake of 0.0925 mg elemental zinc per day was considered too low to be considered a supplementation study. The 2 studies included in the meta-analysis provided zinc sulfate to children and adolescents, with doses of 9 mg/day^48^ and 50 mg/day^53^ of elemental zinc, respectively, and pooled analysis found no statistically significant effect of zinc supplementation on IGF-1 (Table 4). Given the small number of studies it is unclear whether IGF-1 is an effective biomarker of zinc intake. The GRADE quality-of-evidence assessment for IGF-1 was very low (Information S2).

#### Brain-derived neurotrophic factor

Three studies reported brain-derived neurotrophic factor (BDNF), but only data from 2 studies^80^^,^^109^ were included in the meta-analysis since 1 study^127^ reported values reported as mean change. As shown in Table 4, the effect of zinc supplementation was not statistically significant. It remains unclear whether BDNF is an effective biomarker of zinc due to the paucity of data. The GRADE quality-of-evidence assessment for BDNF was very low (Information S2).

#### Total antioxidant capacity

The effect of zinc supplementation on total antioxidant capacity (TAC) was reported in 3 studies.^80^^,^^94^^,^^110^ Zinc supplementation had a statistically significant effect on TAC (MD: 116.96; 95% CI: 25.46–208.45); however, heterogeneity was high (*I^2^ *=* *86.6%) (Table 4). Therefore, it is unclear if TAC is an effective biomarker. The GRADE quality-of-evidence assessment for TAC was very low (Information S2).

#### Other biomarkers

The original review reported a single study^87^ that measured the exchangeable zinc pool (EZP) in response to changes in zinc intake. The updated review process retrieved 1 additional study^88^ that met the inclusion criteria. Combining these data resulted in a significant response to zinc intake (Table 4); however, the limited number of studies meant that this could not be confirmed as an effective biomarker.

The original review reported 7 studies that assessed erythrocyte zinc concentration (µmol/L). The updated search identified 1 additional study providing additional erythrocyte zinc data,^66^ but the values could not be combined as data were expressed as mmol/g Hb and thus no new meta-analysis was conducted. Results from the original review suggested that erythrocyte zinc does not appear to be an effective biomarker. In terms of new potential biomarkers, 2 studies reported data on arachidonic acid (ARA),^92^^,^^110^ 2 studies reported on DNA fragmentation,^81^^,^^110^ 2 studies reported on total body zinc clearance (CZn),^86^^,^^120^ and 2 studies reported on gene expression of *ZnT1*.^51^^,^^97^ In 1 study measuring CZn^120^ and 1 study measuring ARA^92^ it was not possible to obtain the values as means and SDs; therefore, it was not possible to complete these analyses. Moreover, it was not possible to harmonize the units for the studies assessing DNA fragmentation^81^^,^^110^ and gene expression of *ZnT1*^51^^,^^97^; therefore, these were also not included in the meta-analyses. Results from the studies assessing DNA fragmentation and total body zinc clearance showed that these biomarkers responded to changes in zinc intake. Results from 1 study assessing ARA showed that zinc supplementation did not affect the levels of ARA, whereas the other showed that ARA levels were not affected by a zinc supplement being taken with or without food. For gene expression, zinc supplementation did not change *ZnT1* mRNA abundance in 1 study,^51^ whereas zinc supplementation led to an increase in the expression of ZnT1.^97^

Other potential biomarkers that appeared to respond to changes in zinc intake but were only reported in single studies were as follows: erythrocyte osmotic fragility (%),^66^ gene expression of *Zip4*,^51^ gene expression of *Zip8*,^51^ gene expression of *ZnT1*,^51^ kinetics parameters of a venous zinc tolerance test,^86^ plasma conjugated dienes (nmol g^-1^ total lipid),^66^ plasma Zn:Cu ratio,^91^ secretory phospholipase,^45^ serum retinol,^38^ and expression of Znt5.^97^ A summary of these biomarkers is presented in Information S3.

## DISCUSSION

### Summary of main findings

The original EURRECA search protocol to review methods of assessment of zinc status in humans was re-run to include studies published between January 2007 and September 2022. The updated search identified 50 additional studies from 54 articles that provided new data for 7 of the 32 original biomarkers identified—namely, plasma/serum zinc concentration, urinary zinc excretion, hair and nail zinc concentration, alkaline phosphatase activity, and SOD activity. In addition, 48 potential new biomarkers were identified, 13 of which had sufficient data for inclusion in meta-analyses. All but 1 of the studies identified in the updated search were zinc supplementation trials, and 1 was a depletion trial.^54^ There has been a notable increase in the number of studies being conducted in adolescents, which was previously an under-investigated population group.

Plasma/serum zinc concentration continues to be the most frequently reported biomarker for zinc and is the only zinc biomarker for which there are widely accepted cutoff values for deficiency that are used clinically to indicate deficiency in individuals.^6^ The addition of new data increased the number of participants from 1454 (all study types combined) to 4316 in controlled trials and 2985 in before-and-after trials (Table 5). Findings from both sets of trials concurred with the original review, in that there was an overall significant response of plasma/serum zinc concentration to dietary zinc intake in infants, children and adolescents, adults, elderly people, men, women, and those with low and moderate/normal status at baseline. Importantly, this update clarifies the evidence for the plasma/serum zinc response to dietary zinc supplementation in children and adolescents, which was a limitation in the original review due to a lack of data in this age group. The updated review included data from 1789 and 1202 children and adolescents from controlled and before-and-after studies, respectively, compared with data from only 17 participants in the original review. However, for other population groups, including pregnant, lactating, and postmenopausal women, subgroup analysis was unable to provide further clarity on the usefulness of plasma/serum zinc concentration as a biomarker, which is somewhat in contrast to the original review,^11^ which combined controlled and before-and-after studies and reported a significant response of plasma/serum zinc concentration to dietary zinc intake in pregnant and lactating women (MD: 0.37; 95% CI: 0.32–0.43). Much discussion and controversy surrounds plasma zinc as a biomarker of zinc status, not least because it represents less than 0.1% of total body zinc and must be interpreted carefully in light of the known confounders described earlier. However, zinc isotope studies confirm that it is an important component of the mobilizable (or exchangeable) zinc pool,^8^ and thus continues to be the biomarker of choice for assessment of individual as well as population zinc status. The original review included measurement of the EZP as 1 of the identified biomarkers. At that time, only 1 study comprising 5 participants met the inclusion criteria. Our updated search identified 1 further study that measured the EZP in infants.^88^ Combining the 2 studies in the meta-analysis showed a significant effect of zinc intake on the size of the EZP; however, the small number of included studies meant that it did not meet our threshold criteria for confirming efficacy as a biomarker.

Urinary zinc excretion was reported in 5 additional articles,^51^^,^^54^^,^^63^^,^^66^^,^^84^ which were combined with the 9 studies^31^^,^^32^^,^^46^^,^^60^^,^^87^^,^^95^^,^^106^^,^^115^^,^^119^ in the original meta-analysis. The overall effect of zinc supplementation on urinary zinc excretion was significant when expressed as µmol/day and mmol/mol creatinine, and in adult populations when expressed as µmol/L. This agrees with, and adds new evidence to, the original review, where only studies that reported urinary zinc excretion expressed as mmol/mol creatinine could be pooled. Despite the inclusion of additional studies, subgroup analyses were limited and could not provide further clarity on the use of urinary zinc concentration in different population groups.

The updated search identified 1 study that provided data for both hair and nail zinc concentrations. When these were combined with the 3 studies reporting hair zinc concentration and 1 study reporting nail zinc concentration from the original review, both resulted in a significant response to dietary zinc intake. They failed, however, to meet the criteria (Table 2) for an effective biomarker, and their usefulness remains unclear.

For plasma alkaline phosphatase activity, 1 additional study was added to those found in the original review. The combined analysis concurred with the original review that this is not an effective biomarker of zinc status. For SOD activity, after the addition of new plasma and erythrocyte data, the usefulness of this as a biomarker of zinc intake remains unclear.

In terms of the potential new biomarkers identified by the updated search, 7 had sufficient data to allow meta-analyses. None, however, met the criteria for an effective biomarker of zinc status (Table 2), either because there were fewer trials reporting the biomarker (IGF-1, IL-6, BDNF, TAC) or there were fewer than 50 participants in each arm of the study (HOMA-IR, fasting insulin).

### Other biomarkers

An extensive list of potential biomarkers is presented in Information S3 for which there were insufficient data to allow for meta-analyses, but nevertheless warrant discussion. The outcome measures were selected as potential biomarkers because there was evidence from the literature of a plausible mechanistic link to zinc status. Recent studies suggest that the activity of zinc-dependent enzymes involved in fatty acid metabolism, such as the fatty acid desaturases (FADS1 and FADS2), may be sensitive to changes in dietary zinc intake, thus impacting the ratio of fatty acid metabolites circulating in the blood, such as the dihomo-γ-linolenic acid (DGLA) to γ-linolenic acid (GLA) molar ratios.^128^ DNA fragmentation, an indicator of DNA damage measured using the comet assay, has also been explored as a biomarker for zinc intake due to the role of zinc as an antioxidant (eg, as a cofactor for copper/zinc SOD), thus protecting DNA from free radical damage. In addition, zinc plays a role in transcription and replication of DNA through zinc finger proteins and as a cofactor for proteins involved in DNA repair.^129^

Glucose metabolism also has a mechanistic link to zinc status through the role of zinc in insulin storage and release. Recent reviews suggest that indices of glucose metabolism (glycated hemoglobin [HbA1c], HOMA-IR, fasting glucose) may improve following zinc supplementation in diabetic or prediabetic patients.^130^^,^^131^

### Strengths and limitations

To enable data from the updated search to be merged with the dataset from the original review, we adhered to the original search protocol and inclusion/exclusion criteria set out by the EURRECA consortium.^17^ A limitation is that this did not allow for the inclusion of studies that used a food-based vehicle for increasing dietary zinc intake (fortification or biofortification), of which there has been a rapid increase in recent years. Similarly, the chemical formulation of the zinc supplement was restricted to sulfate, citrate, and acetate, which were deemed the most easily absorbed by the EURRECA consortium. Other zinc formulations, such as zinc gluconate and citrate,^132^ are also frequently used in human trials and may provide useful additional biomarker data. As we found in the original review, the heterogeneity between the studies was generally very high, which is a common phenomenon in the meta-analysis of human nutrition studies. This impacts the risk-of-bias scores, such that, for most of the biomarkers, there is a high level of uncertainty in the findings.

A strength of this review is that it builds directly on the original EURRECA review, following the same search protocol and inclusion/exclusion criteria, thus allowing data from the original review to be combined with new data identified in the updated search. In addition, we were able to undertake a more rigorous risk-of-bias analysis with the tools that have become available since 2007. Methodologically, we have adhered closely to the original meta-analysis techniques but have been informed by recent advances and recommendations for best practice when pooling data from different study designs. To that end, we separated controlled trials (RCTs, quasi-controlled trials, non-RCTs) from before-and-after studies. In addition, we were able to extract baseline and endline data from participants in the controlled trials where baseline data were presented and included these with the data from before-and-after studies, thus enabling a larger dataset for pooled analyses.

### Implications for future research

This systematic review and meta-analysis, alongside the original review undertaken over a decade ago, reveals that, although there is a plethora of plausible new potential indicators of zinc status, we are still a long way from confirming their reliability and sensitivity and more high-quality studies are needed before threshold values for status identification purposes can be established. Plasma/serum zinc concentration remains the most widely used biomarker despite the well-documented caveats^1^ and cutoff values for identification of deficiency status have been established.^6^ More efforts to develop algorithms to mitigate these caveats, such as those suggested for introducing corrections for the impact of concurrent inflammation on plasma/serum zinc levels, are warranted.^133^ Ultimately, acknowledging that zinc is a type 2 nutrient, and thus deficiency presents a variety of nonspecific clinical and subclinical consequences, it is likely that a statistical model that includes 3 or more biomarkers in combination is needed to yield a robust and reliable means to assess zinc status and monitor the impact of changes in zinc intake. A concept described as the “zinc status index” was developed using data from an animal model, and combines fatty acid ratios with the mRNA expression of zinc-related proteins and gut microbiome profiling.^134^ Future research aimed at exploring this concept using data from human studies may move things forward significantly in the next decade.

## CONCLUSION

In this updated review, additional data for 7 of the 32 previously reported biomarkers were identified in addition to 40 new putative biomarkers from studies published since 2007. Plasma/serum zinc concentration remained the most frequently used biomarker to assess zinc status, responding to changes in zinc intake in studies of healthy infants, children and adolescents, adults, and elderly individuals or when taken in the form of zinc sulfate or gluconate. Yet, evidence gaps persist in identifying its usefulness in specific populations, such as pregnant, lactating, and postmenopausal women, as well as when supplements are provided in the form of zinc acetate. Urinary zinc excretion also responded to changes in zinc intake; however, the small number of additional studies identified from the updated search limited further insight of its applicability across different demographic groups and supplementation types and doses. Hair and nail zinc, serum SOD activity, erythrocyte SOD activity, EZP, fasting blood glucose, fasting insulin, insulin resistance, IL-6, IGF-1, BDNF, and TAC were included in the meta-analyses to assess their usefulness as potential biomarkers. While some biomarkers exhibited a statistically significant response to dietary zinc intake (ie, fasting insulin, IL-6, EZP, and TAC), they did not meet the criteria for an effective biomarker, and therefore their usefulness remains unclear. Further high-quality evidence is required to explore novel biomarkers to assess zinc status in diverse populations.

## Supplementary Material

nuae072_Supplementary_Data

## Data Availability

Data-collection forms, data extracted from included studies, and data used for all analyses are available upon request to the corresponding author. Forest plots, funnel plots, Galbraith plots, and leave-one-out sensitivity analysis can be found in our open-access institutional data repository (https://uclandata.uclan.ac.uk/id/eprint/449).

## References

[1] King JC , Brown KH, Gibson RS, et al Biomarkers of Nutrition for Development (BOND)—zinc review. J Nutr. 2015;146(4):858S-885S. 26962190 10.3945/jn.115.220079 PMC4807640

[2] Brown KH , Wuehler SE, Peerson JM. The importance of zinc in human nutrition and estimation of the global prevalence of zinc deficiency. Food Nutr Bull. 2001;22(2):113-125.

[3] Brown KH , Peerson JM, Rivera J, Allen LH. Effect of supplemental zinc on the growth and serum zinc concentrations of prepubertal children: a meta-analysis of randomized controlled trials. Am J Clin Nutr. 2002;75(6):1062-1071. 12036814 10.1093/ajcn/75.6.1062

[4] Prasad AS. Discovery of human zinc deficiency: its impact on human health and disease. Adv Nutr. 2013;4(2):176-190. 23493534 10.3945/an.112.003210 PMC3649098

[5] Fischer Walker CL , Ezzati M, Black RE. Global and regional child mortality and burden of disease attributable to zinc deficiency. Eur J Clin Nutr. 2009;63(5):591-597. 18270521 10.1038/ejcn.2008.9

[6] Hess SY , Lönnerdal B, Hotz C, et al; International Zinc Nutrition Consultative Group (IZiNCG). International Zinc Nutrition Consultative Group (IZiNCG) technical document #1. Food Nutr Bull. 2004;30(Suppl 1):S5-S11. 18046856

[7] Gupta S , Brazier AKM, Lowe NM. Zinc deficiency in low‐ and middle‐income countries: prevalence and approaches for mitigation. J Hum Nutr Diet. 2020;33(5):624-643. 32627912 10.1111/jhn.12791

[8] King JC. Zinc: an essential but elusive nutrient. Am J Clin Nutr. 2011;94(2):679S-684S. 21715515 10.3945/ajcn.110.005744 PMC3142737

[9] Lowe NM. Assessing zinc in humans. Curr Opin Clin Nutr Metab Care. 2016;19(5):321-327. 27348152 10.1097/MCO.0000000000000298

[10] Frederickson CJ , Fleming DEB, Asael D, et al Single hair analysis by X-ray fluorescence spectrometry detects small changes in dietary zinc intake: a nested randomized controlled trial. Front Nutr. 2023;10:1139017. 37032778 10.3389/fnut.2023.1139017 PMC10080032

[11] Lowe NM , Fekete K, Decsi T. Methods of assessment of zinc status in humans. Am J Clin Nutr. 2009;89(6):2040S-2051S. 19420098 10.3945/ajcn.2009.27230G

[12] Liong EM , McDonald CM, Suh J, et al Zinc-biofortified wheat intake and zinc status biomarkers in men: randomized controlled trial. J Nutr. 2021;151(7):1817-1823. 34036355 10.1093/jn/nxab092

[13] Wessells KR , Brown KH, Arnold CD, et al Plasma and nail zinc concentrations, but not hair zinc, respond positively to two different forms of preventive zinc supplementation in young Laotian children: a randomized controlled trial. Biol Trace Elem Res. 2021;199(2):442-452. 32356207 10.1007/s12011-020-02163-2 PMC7746564

[14] Zyba SJ , Shenvi SV, Killilea DW, et al A moderate increase in dietary zinc reduces DNA strand breaks in leukocytes and alters plasma proteins without changing plasma zinc concentrations. Am J Clin Nutr. 2017;105(2):343-‐351. 28003206 10.3945/ajcn.116.135327 PMC5267297

[15] Knez M , Stangoulis J, Glibetic M, Tako E. The linoleic acid: dihomo-γ-linolenic acid ratio (LA: DGLA)—an emerging biomarker of Zn status. Nutrients. 2017;9(8):825 28763004 10.3390/nu9080825 PMC5579618

[16] Page MJ , McKenzie JE, Bossuyt PM, et al The PRISMA 2020 statement: an updated guideline for reporting systematic reviews. BMJ. 2021;372:n71. 33782057 10.1136/bmj.n71 PMC8005924

[17] Hooper L , Ashton K, Harvey LJ, Decsi T, Fairweather-Tait SJ. Assessing potential biomarkers of micronutrient status by using a systematic review methodology: methods. Am J Clin Nutr. 2009;89(6):1953S- 1959S. 19403633 10.3945/ajcn.2009.27230A

[18] Knez M , Boy E. Existing knowledge on Zn status biomarkers (1963–2021) with a particular focus on FADS1 and FADS2 diagnostic performance and recommendations for further research. Front Nutr. 2022;9:1057156. 36712514 10.3389/fnut.2022.1057156 PMC9878572

[19] Ouzzani M , Hammady H, Fedorowicz Z, Elmagarmid A. Rayyan—a web and mobile app for systematic reviews. Syst Rev. 2016;5(1):210. 27919275 10.1186/s13643-016-0384-4 PMC5139140

[20] Sterne JAC , Savović J, Page MJ, et al RoB 2: a revised tool for assessing risk of bias in randomised trials. BMJ. 2019;366:l4898. 31462531 10.1136/bmj.l4898

[21] Sterne JA , Hernán MA, Reeves BC, et al ROBINS-I: a tool for assessing risk of bias in non-randomised studies of interventions. BMJ. 2016;355:i4919. 27733354 10.1136/bmj.i4919 PMC5062054

[22] Guyatt GH , Oxman AD, Schünemann HJ, Tugwell P, Knottnerus A. GRADE guidelines: a new series of articles in the Journal of Clinical Epidemiology. J Clin Epidemiol. 2011;64(4):380-382. 21185693 10.1016/j.jclinepi.2010.09.011

[23] Drahota A , Beller E. Cochrane RevMan calculator. Cochrane training. Published 2023. Accessed August 1, 2023. https://training.cochrane.org/resource/revman-calculator

[24] Unitslab.com. Zinc. Published 2023. Accessed August 1, 2023. https://unitslab.com/node/142

[25] Diabetes.co.uk. Blood sugar converter. Published 2019. Accessed August 1, 2023. https://www.diabetes.co.uk/blood-sugar-converter.html

[26] Higgins J , Thomas J, Chandler J, et al How to include multiple groups from one study. *Cochrane Handbook for Systematic Reviews of Interventions*, version 6.4. Accessed November 1, 2023. https://handbook-5-1.cochrane.org/chapter_16/16_5_4_how_to_include_multiple_groups_from_one_study.htm#:∼:text=The%20recommended%20method%20in%20most,into%20a%20single%20control%20group

[27] Higgins JPT , Thompson SG, Deeks JJ, Altman DG. Measuring inconsistency in meta-analyses. BMJ. 2003;327(7414):557-560. 12958120 10.1136/bmj.327.7414.557 PMC192859

[28] Higgins J , Thomas J, Chandler J, et al 10.4.3.1 Recommendations on testing for funnel plot asymmetry. *Cochrane Handbook for Systematic Reviews of Interventions*, version 6.4. Accessed November 1, 2023. https://handbook-5-1.cochrane.org/chapter_10/10_4_3_1_recommendations_on_testing_for_funnel_plot_asymmetry.htm

[29] Hodkinson CF , Kelly M, Alexander HD, et al Effect of zinc supplementation on the immune status of healthy older individuals aged 55-70 years: the ZENITH study. J Gerontol A Biol Sci Med Sci. 2007;62(6):598-608. 17595415 10.1093/gerona/62.6.598

[30] Medeiros DM , Mazhar A, Brunett EW. Failure of oral zinc supplementation to alter hair zinc levels among healthy human males. Nutr Res. 1987;7(11):1109-1115.

[31] Hininger-Favier I , Andriollo-Sanchez M, Arnaud J, et al Age- and sex-dependent effects of long-term zinc supplementation on essential trace element status and lipid metabolism in European subjects: the Zenith study. Br J Nutr. 2007;97(3):569-578. 17313720 10.1017/S0007114507432974

[32] Black M , Medeiros D, Brunett E, Welke R. Zinc supplements and serum lipids in young adult white males. Am J Clin Nutr. 1988;47(6):970-975. 3163879 10.1093/ajcn/47.6.970

[33] O'Brien CE , Krebs NF, Westcott JL, Dong F. Relationships among plasma zinc, plasma prolactin, milk transfer, and milk zinc in lactating women. J Hum Lact. 2007;23(2):179-183. 17478870 10.1177/0890334407300021

[34] Grider A , Bailey LB, Cousins RJ. Erythrocyte metallothionein as an index of zinc status in humans. Proc Natl Acad Sci USA. 1990;87(4):1259-1262. 2304897 10.1073/pnas.87.4.1259 PMC53453

[35] Abdollahi M , Ajami M, Abdollahi Z, et al Zinc supplementation is an effective and feasible strategy to prevent growth retardation in 6-to-24-month children: a pragmatic double blind, randomized trial. Heliyon. 2019;5(11):e02581. 31720482 10.1016/j.heliyon.2019.e02581 PMC6839004

[36] Abdulla M , Suck C. Blood levels of copper, iron, zinc, and lead in adults in India and Pakistan and the effect of oral zinc supplementation for six weeks. Biol Trace Elem Res. 1998;61(3):323-331. 9533570 10.1007/BF02789092

[37] Abdulla M , Svensson S. Effect of oral zinc intake on δ-aminolaevulinic acid dehydratase in red blood cells. Scand J Clin Lab Invest. 1979;39(1):31-36. 523952 10.3109/00365517909104936

[38] Adriani M , Wirjatmadi B. The effect of adding zinc to vitamin A on IGF-1, bone age and linear growth in stunted children. J Trace Elem Med Biol. 2014;28(4):431-435. 25439136 10.1016/j.jtemb.2014.08.007

[39] Ahmadi H , Mazloumi-Kiapey SS, Sadeghi O, et al Zinc supplementation affects favorably the frequency of migraine attacks: a double-blind randomized placebo-controlled clinical trial. Nutr J. 2020;19(1):101 32928216 10.1186/s12937-020-00618-9 PMC7491175

[40] Allan AK , Hawksworth GM, Woodhouse LR, Sutherland B, King JC, Beattie JH. Lymphocyte metallothionein mRNA responds to marginal zinc intake in human volunteers. Br J Nutr. 2000;84(5):747-756. 11177190

[41] Attia JR , Holliday E, Weaver N, et al The effect of zinc supplementation on glucose homeostasis: a randomised double-blind placebo-controlled trial. Acta Diabetol. 2022;59(7):965-975. 35451678 10.1007/s00592-022-01888-x PMC9026040

[42] Ayatollahi H , Rajabi E, Yekta Z, Jalali Z. Efficacy of oral zinc sulfate supplementation on clearance of cervical human papillomavirus (HPV): a randomized controlled clinical trial. Asian Pac J Cancer Prev. 2022;23(4):1285-1290. 35485687 10.31557/APJCP.2022.23.4.1285 PMC9375629

[43] Ba Lo N , Aaron GJ, Hess SY, et al Plasma zinc concentration responds to short-term zinc supplementation, but not zinc fortification, in young children in Senegal. Am J Clin Nutr. 2011;93(6):1348-1355. 21490143 10.3945/ajcn.111.012278

[44] Bales CW , DiSilvestro RA, Currie KL, et al Marginal zinc deficiency in older adults: responsiveness of zinc status indicators. J Am Coll Nutr. 1994;13(5):455-462. 7836623 10.1080/07315724.1994.10718434

[45] Bao B , Prasad AS, Beck FW, et al Zinc decreases C-reactive protein, lipid peroxidation, and inflammatory cytokines in elderly subjects: a potential implication of zinc as an atheroprotective agent. Am J Clin Nutr. 2010;91(6):1634-1641. 20427734 10.3945/ajcn.2009.28836 PMC2869512

[46] Barrie SA , Wright JV, Pizzorno JE, Kutter E, Barron PC. Comparative absorption of zinc picolinate, zinc citrate and zinc gluconate in humans. Agents Actions. 1987;21(1-2):223-228. 3630857 10.1007/BF01974946

[47] Becquey E , Ouédraogo CT, Hess SY, et al Comparison of preventive and therapeutic zinc supplementation in young children in Burkina Faso: a cluster-randomized, community-based trial. J Nutr. 2016;146(10):2058-2066. 27489011 10.3945/jn.116.230128

[48] Berger PK , Pollock NK, Laing EM, et al Zinc supplementation increases procollagen type 1 amino-terminal propeptide in premenarcheal girls: a randomized controlled trial. J Nutr. 2015;145(12):2699-2704. 26491117 10.3945/jn.115.218792 PMC4656906

[49] Bertinato J , Simpson JR, Sherrard L, et al Zinc supplementation does not alter sensitive biomarkers of copper status in healthy boys. J Nutr. 2012;143(3):284-289. 10.3945/jn.112.171306 23303874

[50] Bogden J , Oleske J, Lavenhar M, et al Zinc and immunocompetence in elderly people: effects of zinc supplementation for 3 months. Am J Clin Nutr. 1988;48(3):655-663. 3414581 10.1093/ajcn/48.3.655

[51] Bogale A , Clarke SL, Fiddler J, Hambidge KM, Stoecker BJ. Zinc supplementation in a randomized controlled trial decreased ZIP4 and ZIP8 mRNA abundance in peripheral blood mononuclear cells of adult women. Nutr Metab Insights. 2015;8:7-14. 26023281 10.4137/NMI.S23233 PMC4431478

[52] Brown KH , de Romaña DL, Arsenault JE, Peerson JM, Penny ME. Comparison of the effects of zinc delivered in a fortified food or a liquid supplement on the growth, morbidity, and plasma zinc concentrations of young Peruvian children. Am J Clin Nutr. 2007;85(2):538-547. 17284755 10.1093/ajcn/85.2.538

[53] Cesur Y , Yordaman N, Doğan M. Serum insulin-like growth factor-I and insulin-like growth factor binding protein-3 levels in children with zinc deficiency and the effect of zinc supplementation on these parameters. J Pediatr Endocrinol Metab. 2009;22(12):1137-1143. 20333873 10.1515/jpem.2009.22.12.1137

[54] Chung CS , Stookey J, Dare D, et al Current dietary zinc intake has a greater effect on fractional zinc absorption than does longer term zinc consumption in healthy adult men. Am J Clin Nutr. 2008;87(5):1224-1229. 18469243 10.1093/ajcn/87.5.1224

[55] Crouse SF , Hooper PL, Atterbom HA, Papenfuss RL. Zinc ingestion and lipoprotein values in sedentary and endurance-trained men. JAMA. 1984;252(6):785-787. 6748177

[56] de Brito N , Rocha É, de Araújo Silva A, et al Oral zinc supplementation decreases the serum iron concentration in healthy schoolchildren: a pilot study. Nutrients. 2014;6(9):3460-3473. 25192026 10.3390/nu6093460 PMC4179171

[57] Deguchi M , Jose H, Nishida K, Ooi K. Transepidermal water loss (TEWL)-decreasing effect by administration of zinc in the elderly people. Yakugaku Zasshi. 2020;140(2):313-318. 32009050 10.1248/yakushi.19-00198

[58] Demetree JW , Safer LF, Artis WM. The effect of zinc on the sebum secretion rate. Acta Derm Venereol. 1980;60(2):166-169. 6155029

[59] DiSilvestro RA , Koch E, Rakes L. Moderately high dose zinc gluconate or zinc glycinate: effects on plasma zinc and erythrocyte superoxide dismutase activities in young adult women. Biol Trace Elem Res. 2015;168(1):11-14. 25877802 10.1007/s12011-015-0334-3

[60] Donangelo CM , Woodhouse LR, King SM, Viteri FE, King JC. Supplemental zinc lowers measures of iron status in young women with low iron reserves. J Nutr. 2002;132(7):1860-1864. 12097660 10.1093/jn/132.7.1860

[61] Duchateau J , Delespesse G, Vereecke P. Influence of oral zinc supplementation on the lymphocyte response to mitogens of normal subjects. Am J Clin Nutr. 1981;34(1):88-93. 7446464 10.1093/ajcn/34.1.88

[62] Eskici G , Gunay M, Baltaci AK, Mogulkoc R. The effect of different doses of zinc supplementation on serum element and lactate levels in elite volleyball athletes. J Appl Biomed. 2017;15(2):133-138.

[63] Eskici G , Gunay M, Baltaci AK, Mogulkoc R. The effect of zinc supplementation on the urinary excretion of elements in female athletes. Pak J Pharm Sci. 2016;29(1):125-129. 26826808

[64] Fahmida U , Rumawas JS, Utomo B, Patmonodewo S, Schultink W. Zinc-iron, but not zinc-alone supplementation, increased linear growth of stunted infants with low haemoglobin. Asia Pac J Clin Nutr. 2007;16(2):301-309. 17468087

[65] Farrell CP , Morgan M, Rudolph DS, et al Proton pump inhibitors interfere with zinc absorption and zinc body stores. Gastroenterology Res. 2011;4(6):243-251. 27957023 10.4021/gr379w PMC5139861

[66] Fernandes de Oliveira KJ , Donangelo CM, de Oliveira AV, da Silveira CLP, Koury JC. Effect of zinc supplementation on the antioxidant, copper, and iron status of physically active adolescents. Cell Biochem Funct. 2009;27(3):162-166. 19277992 10.1002/cbf.1550

[67] Field HP , Whitley AJ, Srinivasan TR, Walker BE, Kelleher J. Plasma and leucocyte zinc concentrations and their response to zinc supplementation in an elderly population. Int J Vitam Nutr Res. 1987;57(3):311-317. 3679703

[68] Fischer PWF , L’Abbe MR, Giroux A. Effect of zinc supplementation on copper status in humans. Fed Proc. 1984;43(3):743-746. 10.1093/ajcn/40.4.743 6486080

[69] Freeland-Graves JH , Hendrickson PJ, Ebangit ML, Snowden JY. Salivary zinc as an index of zinc status in women fed a low-zinc diet. Am J Clin Nutr. 1981;34(3):312-321. 7211732 10.1093/ajcn/34.3.312

[70] Gatto LM , Samman S. The effect of zinc supplementation on plasma lipids and low-density lipoprotein oxidation in males. Free Radic Biol Med. 1995;19(4):517-521. 7590403 10.1016/0891-5849(95)00041-u

[71] Gomes Dantas Lopes MM , de Brito NJN, Dantas de Medeiros Rocha É, França MC, de Almeida MdG, Brandão-Neto J. Nutritional assessment methods for zinc supplementation in prepubertal non-zinc-deficient children. Food Nutr Res. 2015;59(1):29733 26507491 10.3402/fnr.v59.29733 PMC4623288

[72] Gülsan M , Malbora B, Avcı Z, Bayraktar N, Bozkaya I, Ozbek N. Effects of zinc sulfate supplementation in treatment of iron deficiency anemia. Turk J Haematol. 2013;30(2):144-152. 24385777 10.4274/Tjh.2012.0043 PMC3878474

[73] Gupta R , Garg VK, Mathur DK, Goyal RK. Oral zinc therapy in diabetic neuropathy. J Assoc Physicians India. 1998;46(11):939-942. 11229219

[74] Hayee MA , Mohammad QD, Haque A. Diabetic neuropathy and zinc therapy. Bangladesh Med Res Counc Bull. 2005;31(2):62-67. 16967811

[75] Heckmann SM , Hujoel P, Habiger S, et al Zinc gluconate in the treatment of dysgeusia—a randomized clinical trial. J Dent Res. 2005;84(1):35-38. 15615872 10.1177/154405910508400105

[76] Hollingsworth JW , Otte RG, Boss GR, Fryberger MF, Strause LG, Saltman P. Immunodeficiency and lymphocyte ecto-5′-nucleotidase activity in the elderly: a comparison of the effect of a trace mineral supplement (1 USRDA) with high zinc (6.7 X USRDA). Nutr Res. 1987;7(8):801-811.

[77] Hunt I , Murphy N, Cleaver A, et al Zinc supplementation during pregnancy in low-income teenagers of Mexican descent: effects on selected blood constituents and on progress and outcome of pregnancy. Am J Clin Nutr. 1985;42(5):815-828. 4061343 10.1093/ajcn/42.5.815

[78] Islam MR , Attia J, Ali L, et al Zinc supplementation for improving glucose handling in pre-diabetes: a double blind randomized placebo controlled pilot study. Diabetes Res Clin Pract. 2016;115:39-46. 27242121 10.1016/j.diabres.2016.03.010

[79] Islam M , Black R, Krebs NF, et al Different doses, forms, and frequencies of zinc supplementation for the prevention of diarrhea and promotion of linear growth among young Bangladeshi children: a six-arm, randomized, community-based efficacy trial. J Nutr. 2022;152(5):1306-1315. 35015856 10.1093/jn/nxab439

[80] Jafari F , Amani R, Tarrahi MJ. Effect of zinc supplementation on physical and psychological symptoms, biomarkers of inflammation, oxidative stress, and brain-derived neurotrophic factor in young women with premenstrual syndrome: a randomized, double-blind, placebo-controlled trial. Biol Trace Elem Res. 2020;194(1):89-95. 31154571 10.1007/s12011-019-01757-9

[81] Joray ML , Yu TW, Ho E, et al Zinc supplementation reduced DNA breaks in Ethiopian women. Nutr Res. 2015;35(1):49-55. 25491347 10.1016/j.nutres.2014.10.006 PMC4466114

[82] Kaseb F , Fallah R. Efficacy of zinc supplementation on improvement of weight and height growth of healthy 9-18 year children. World Appl Sci J. 2013;26(2):189-193.

[83] Khorsandi H , Nikpayam O, Yousefi R, et al Zinc supplementation improves body weight management, inflammatory biomarkers and insulin resistance in individuals with obesity: a randomized, placebo-controlled, double-blind trial. Diabetol Metab Syndr. 2019;11(1):101 31827626 10.1186/s13098-019-0497-8 PMC6889702

[84] Kim J , Ahn J. Effect of zinc supplementation on inflammatory markers and adipokines in young obese women. Biol Trace Elem Res. 2014;157(2):101-106. 24402636 10.1007/s12011-013-9885-3

[85] Kim J , Lee S. Effect of zinc supplementation on insulin resistance and metabolic risk factors in obese Korean women. Nutr Res Pract. 2012;6(3):221-225. 22808346 10.4162/nrp.2012.6.3.221 PMC3395787

[86] Leite LD , De Medeiros Rocha ÉD, Almeida MDG, et al Sensitivity of zinc kinetics and nutritional assessment of children submitted to venous zinc tolerance test. J Am Coll Nutr. 2009;28(4):405-412. 20368379 10.1080/07315724.2009.10718103

[87] Lowe NM , Woodhouse LR, Sutherland B, et al Kinetic parameters and plasma zinc concentration correlate well with net loss and gain of zinc from men. J Nutr. 2004;134(9):2178-2181. 15333701 10.1093/jn/134.9.2178

[88] Long JM , Khandaker AM, Sthity RA, et al Exchangeable zinc pool size reflects form of zinc supplementation in young children and is not associated with markers of inflammation. Nutrients. 2022;14(3):481. 35276840 10.3390/nu14030481 PMC8838617

[89] Lukaski HC , Bolonchuk WW, Klevay LM, Milne DB, Sandstead HH. Changes in plasma zinc content after exercise in men fed a low-zinc diet. Am J Physiol. 1984;247 (1 Pt 1):E88-93. 6742191 10.1152/ajpendo.1984.247.1.E88

[90] Mahajan S , Prasad A, Brewer G, Zwas F, Doh-Yeel L. Effect of changes in dietary zinc intake on taste acuity and dark adaptation in normal human subjects. J Trace Elem Exp Med. 1992;5(1):33-45.

[91] Marques LFJC , Donangelo CM, Franco JG, et al Plasma zinc, copper, and serum thyroid hormones and insulin levels after zinc supplementation followed by placebo in competitive athletes. Biol Trace Elem Res. 2011;142(3):415-423. 20809272 10.1007/s12011-010-8821-z

[92] Massih YN , Hall AG, Suh J, King JC. Zinc supplements taken with food increase essential fatty acid desaturation indices in adult men compared with zinc taken in the fasted state. J Nutr. 2021;151(9):2583-2589. 34236435 10.1093/jn/nxab149

[93] Mazaheri Nia L , Iravani M, Abedi P, Cheraghian B. Effect of zinc on testosterone levels and sexual function of postmenopausal women: a randomized controlled trial. J Sex Marital Ther. 2021;47(8):804-813. 34311679 10.1080/0092623X.2021.1957732

[94] Mesdaghinia E , Naderi F, Bahmani F, Chamani M, Ghaderi A, Asemi Z. The effects of zinc supplementation on clinical response and metabolic profiles in pregnant women at risk for intrauterine growth restriction: a randomized, double-blind, placebo-controlled trial. J Matern Fetal Neonatal Med. 2021;34(9):1382-1388. 31248307 10.1080/14767058.2019.1637847

[95] Milne DB , Canfield WK, Gallagher SK, Hunt JR, Klevay LM. Ethanol metabolism in postmenopausal women fed a diet marginal in zinc. Am J Clin Nutr. 1987;46(4):688-693. 3661484 10.1093/ajcn/46.4.688

[96] Mujica-Coopman MF , Borja A, Pizarro F, Olivares M. Effect of daily supplementation with iron and zinc on iron status of childbearing age women. Biol Trace Elem Res. 2015;165(1):10-17. 25582309 10.1007/s12011-014-0226-y

[97] Noh H , Paik HY, Kim J, Chung J. The changes of zinc transporter ZnT gene expression in response to zinc supplementation in obese women. Biol Trace Elem Res. 2014;162(1-3):38-45. 25240971 10.1007/s12011-014-0128-z

[98] Pachotikarn C , Medeiros D, Windham F. Effect of oral zinc supplementation upon plasma lipids, blood pressure, and other variables in young adult white males. Nutr Rep Int. 1985;32(2):373-382.

[99] Palin D , Underwood B, Denning C. The effect of oral zinc supplementation on plasma levels of vitamin A and retinol-binding protein in cystic fibrosis. Am J Clin Nutr. 1979;32(6):1253-1259. 571673 10.1093/ajcn/32.6.1253

[100] Payahoo L , Ostadrahimi A, Mobasseri M, et al Effects of zinc supplementation on the anthropometric measurements, lipid profiles and fasting blood glucose in the healthy obese adults. Adv Pharm Bull. 2013;3(1):161-165. 24312830 10.5681/apb.2013.027 PMC3846058

[101] Peretz A , Nève J, Jeghers O, Pelen F. Zinc distribution in blood components, inflammatory status, and clinical indexes of disease activity during zinc supplementation in inflammatory rheumatic diseases. Am J Clin Nutr. 1993;57(5):690-694. 8480688 10.1093/ajcn/57.5.690

[102] Pinna K , Kelley DS, Taylor PC, King JC, Research C. Immune functions are maintained in healthy men with low zinc intake. J Nutr. 2002;132(7):2033-2036. 12097688 10.1093/jn/132.7.2033

[103] Prasad AS , Mantzoros CS, Beck FWJ, Hess JW, Brewer GJ. Zinc status and serum testosterone levels of healthy adults. Nutrition. 1996;12(5):344-348. 8875519 10.1016/s0899-9007(96)80058-x

[104] Prasad AS , Beck FW, Bao B, et al Zinc supplementation decreases incidence of infections in the elderly: effect of zinc on generation of cytokines and oxidative stress. Am J Clin Nutr. 2007;85(3):837-844. 17344507 10.1093/ajcn/85.3.837

[105] Rohmawati L , Keumala Sari D, Sitepu M, Rusmil K. A randomized, placebo-controlled trial of zinc supplementation during pregnancy for the prevention of stunting: analysis of maternal serum zinc, cord blood osteocalcin and neonatal birth length. Med Glas (Zenica). 2021;18(2):415-420. 33871218 10.17392/1267-21

[106] Ruz M , Cavan KR, Bettger WJ, Gibson RS. Erythrocytes, erythrocyte membranes, neutrophils and platelets as biopsy materials for the assessment of zinc status in humans. Br J Nutr. 1992;68(2):515-527. 1445830 10.1079/bjn19920109

[107] Samman S , Roberts DCK. The effect of zinc supplements on plasma zinc and copper levels and the reported symptoms in healthy volunteers. Med J Aust. 1987;146(5):246-249. 3547053 10.5694/j.1326-5377.1987.tb120232.x

[108] Shaaban SY , El-Hodhod MAA, Nassar MF, Hegazy AET, El-Arab SE, Shaheen FM. Zinc status of lactating Egyptian mothers and their infants: effect of maternal zinc supplementation. Nutr Res. 2005;25(1):45-53.

[109] Solati Z , Jazayeri S, Tehrani-Doost M, Mahmoodianfard S, Gohari MR. Zinc monotherapy increases serum brain-derived neurotrophic factor (BDNF) levels and decreases depressive symptoms in overweight or obese subjects: a double-blind, randomized, placebo-controlled trial. Nutr Neurosci. 2015;18(4):162-168. 24621065 10.1179/1476830513Y.0000000105

[110] Song Y , Chung CS, Bruno RS, et al Dietary zinc restriction and repletion affects DNA integrity in healthy men. Am J Clin Nutr. 2009;90(2):321-328. 19515738 10.3945/ajcn.2008.27300 PMC2709309

[111] Stur M , Tittl M, Reitner A, Meisinger V. Oral zinc and the second eye in age-related macular degeneration. Invest Ophthalmol Vis Sci. 1996;37(7):1225-1235. 8641826

[112] Sullivan VK , Cousins RJ. Competitive reverse transcriptase-polymerase chain reaction shows that dietary zinc supplementation in humans increases monocyte metallothionein mRNA levels. J Nutr. 1997;127(5):694-698. 9164988 10.1093/jn/127.5.694

[113] Sullivan VK , Burnett FR, Cousins RJ. Metallothionein expression is increased in monocytes and erythrocytes of young men during zinc supplementation. J Nutr. 1998;128(4):707-713. 9521632 10.1093/jn/128.4.707

[114] Surono IS , Martono PD, Kameo S, Suradji EW, Koyama H. Effect of probiotic L. plantarum IS-10506 and zinc supplementation on humoral immune response and zinc status of Indonesian pre-school children. J Trace Elem Med Biol. 2014;28(4):465-469. 25183688 10.1016/j.jtemb.2014.07.009

[115] Swanson CA , Mansourian R, Dirren H, Rapin CH. Zinc status of healthy elderly adults: response to supplementation. Am J Clin Nutr. 1988;48(2):343-349. 3407612 10.1093/ajcn/48.2.343

[116] Takacs P , Damjanovich P, Sipos AG, Kozma B. The effect of oral zinc supplementation on cervicovaginal lavage fluid zinc level. Eur J Obstet Gynecol Reprod Biol. 2020 May;248:106-109. 32200246 10.1016/j.ejogrb.2020.03.026

[117] Tamura T , Goldenberg RL, Johnston KE, Freeberg LE, DuBard MB, Thomas EA. In vitro zinc stimulation of angiotensin-converting enzyme activities in human plasma. J Nutr Biochem. 1996;7(1):55-59.

[118] Tamura T , Olin KL, Goldenberg RL, Johnston KE, Dubard MB, Keen CL. Plasma extracellular superoxide dismutase activity in healthy pregnant women is not influenced by zinc supplementation. Biol Trace Elem Res. 2001;80(2):107-113. 11437176 10.1385/BTER:80:2:107

[119] Thomas EA , Bailey LB, Kauwell GA, Lee DY, Cousins RJ. Erythrocyte metallothionein response to dietary zinc in humans. J Nutr. 1992;122(12):2408-2414. 1453226 10.1093/jn/122.12.2408

[120] Vale SHL , Leite LD, Alves CX, et al Zinc pharmacokinetic parameters in the determination of body zinc status in children. Eur J Clin Nutr. 2014;68(2):203-208. 24327117 10.1038/ejcn.2013.250

[121] Wang M , Fan L, Wei W, et al Integrated multi-omics uncovers reliable potential biomarkers and adverse effects of zinc deficiency. Clin Nutr. 2021;40(5):2683-2696. 33933734 10.1016/j.clnu.2021.03.019

[122] Weismann K , Wadskov S, Sondergaard J. Oral zinc sulphate therapy for acne vulgaris. Acta Derm Venereol. 1977;57(4):357-360. 70931

[123] Wessells KR , Jorgensen JM, Hess SY, Woodhouse LR, Peerson JM, Brown KH. Plasma zinc concentration responds rapidly to the initiation and discontinuation of short-term zinc supplementation in healthy men. J Nutr. 2010;140(12):2128-2133. 20943956 10.3945/jn.110.122812

[124] Wessells KR , Ouédraogo ZP, Rouamba N, Hess SY, Ouédraogo JB, Brown KH. Short-term zinc supplementation with dispersible tablets or zinc sulfate solution yields similar positive effects on plasma zinc concentration of young children in Burkina Faso: a randomized controlled trial. J Pediatr. 2012;160(1):129-135.e3. 21871635 10.1016/j.jpeds.2011.06.051

[125] Yadrick MK , Kenney MA, Winterfeldt EA. Iron, copper, and zinc status: response to supplementation with zinc or zinc and iron in adult females. Am J Clin Nutr. 1989;49(1):145-150. 2912000 10.1093/ajcn/49.1.145

[126] Yalda MA , Ibrahiem AA. The effect of combined supplementation of iron and zinc versus iron alone on anemic pregnant patients in Dohuk. Jordan Med J. 2010;44(1):9-16.

[127] Yosaee S , Soltani S, Esteghamati A, et al Effects of zinc, vitamin D, and their co-supplementation on mood, serum cortisol, and brain-derived neurotrophic factor in patients with obesity and mild to moderate depressive symptoms: a phase II, 12-wk, 2 × 2 factorial design, double-blind, randomized, placebo-controlled trial. Nutrition. 2020;71:110601 31837640 10.1016/j.nut.2019.110601

[128] Chimhashu T , Malan L, Baumgartner J, et al Sensitivity of fatty acid desaturation and elongation to plasma zinc concentration: a randomised controlled trial in Beninese children. Br J Nutr. 2018;119(6):610-619. 29352828 10.1017/S000711451700366X

[129] Ho E. Zinc deficiency, DNA damage and cancer risk. J Nutr Biochem. 2004;15(10):572-578. 15542347 10.1016/j.jnutbio.2004.07.005

[130] Pompano LM , Boy E. Effects of dose and duration of zinc interventions on risk factors for type 2 diabetes and cardiovascular disease: a systematic review and meta-analysis. Adv Nutr. 2021;12(1):141-160. 32722790 10.1093/advances/nmaa087 PMC7850144

[131] Wang Z , Ronsmans C, Woolf B. Triangulating evidence for the causal impact of single-intervention zinc supplement on glycaemic control for type 2 diabetes: systematic review and meta-analysis of randomised controlled trial and two-sample Mendelian randomisation. Br J Nutr. 2023;129(11):1929-1944. 35946077 10.1017/S0007114522002616 PMC10167665

[132] Wegmüller R , Tay F, Zeder C, Brnić M, Hurrell RF. Zinc absorption by young adults from supplemental zinc citrate is comparable with that from zinc gluconate and higher than from zinc oxide. J Nutr. 2014;144(2):132-136. 24259556 10.3945/jn.113.181487 PMC3901420

[133] McDonald CM , Suchdev PS, Krebs NF, et al Adjusting plasma or serum zinc concentrations for inflammation: Biomarkers Reflecting Inflammation and Nutritional Determinants of Anemia (BRINDA) project. Am J Clin Nutr. 2020;111(4):927-937. 32266402 10.1093/ajcn/nqz304 PMC7138668

[134] Cheng J , Bar H, Tako E. Zinc Status Index (ZSI) for quantification of zinc physiological status. Nutrients. 2021;13(10):3399. 34684398 10.3390/nu13103399 PMC8541600

