# Supplementary material for: Methods of Assessment of Zinc Status in Humans: An Updated Review and Meta-analysis
Source: Nutr Rev. 2024 Jun 25;83(3):e778–800. doi: 10.1093/nutrit/nuae072 (PMC11819495; doi:10.1093/nutrit/nuae072)
Supplement: nuae072_Supplementary_Data [file nuae072_supplementary_data.zip › nuae072_Supplementary_Data/Supplemental Information 1.pdf]

Supplemental Information 1, search strategy

Contents

Update Search September 2020 1
Medline 1
EMBASE 2
Cochrane Library 2
Update search August 2021 3
Medline 3
Embase 4
Cochrane Library 5
Update Search July 2022 6
Medline 6
Embase 6
Cochrane Library 7

Update Search September 2020

Medline

Date Run: 14/09/2020

ID	Search	Hits
1	exp Zinc/	60203
2	exp Zinc Compounds/ or Zinc Acetate/	13209
3	(zinc or zn).ti,kf,kw.	63442
4	(zinc or zn).ab. /freq=2	74813
5	or/1-4	123942
6	Dietary Supplements/	57724
7	exp Biofortification/	171
8	exp Food, Fortified/	9363
9	exp clinical trial/ or feasibility studies/ or pilot projects/	1015302
10	interven*.mp.	1094590
11	supplement*.mp.	349271
12	(biofortif* or bio fortif*).mp.	1277
13	((fortif* or enrich* or enhanc* or boost*) adj2 (food* or rice or flour or corn or wheat or semolina or pulse* or bread* or sauce* or beverage* or maize or oat* or sugar or salt or oil* or fat* or condiment* or seasoning* or spice* or milk or dairy or juice* or nectar* or cereal* or biscuit* or drink$1 or diet* or beverage* or meal*)).mp.	33673
14	or/6-13	2275616
15	((zinc* or zn*) adj5 deplet*).mp.	1058
16	14 or 15	2276447

17	5 and 16	11771
18	exp animals/ not humans.sh.	4733412
19	17 not 18	8630
20	limit 19 to yr="2007 -Current"	5123

EMBASE

Date Run:	14/09/2020	
ID	Search	Hits
1	(zinc or zn).ti,kw.	73401
2	(zinc or zn).ab. /freq=2	88336
3	1 or 2	116253
4	interven*.mp.	1530456
5	supplement*.mp.	484729
6	(biofortif* or bio fortif*).mp.	1516
7	((fortif* or enrich* or enhanc* or boost*) adj2 (food* or rice or flour or corn or wheat or semolina or pulse* or bread* or sauce* or beverage* or maize or oat* or sugar or salt or oil* or fat* or condiment* or seasoning* or spice* or milk or dairy or juice* or nectar* or cereal* or biscuit* or drink$1 or diet* or beverage* or meal*)).mp.	33512
8	4 or 5 or 6 or 7	1993338
9	((zinc* or zn*) adj5 deplet*).mp.	1227
10	8 or 9	1994278
11	3 and 10	12046
12	exp animal/ not human.sh.	4821510
13	11 not 12	8446
14	limit 13 to yr="2007 -Current"	5496

Cochrane Library

Data base:	Cochrane Library	
Search Name:	SR3. Biomarkers for zinc	
Date Run:	14/09/2020	
ID	Search	Hits
1	MeSH descriptor: [Zinc] explode all trees	1625
2	MeSH descriptor: [Zinc Compounds] explode all trees	536
3	MeSH descriptor: [Zinc Acetate] this term only	24
4	zinc or zn	8459
5	{OR #1-#4}	8459
6	MeSH descriptor: [Dietary Supplements] explode all trees	12252
7	MeSH descriptor: [Biofortification] explode all trees	2
8	MeSH descriptor: [Food, Fortified] explode all trees	1405

9	MeSH descriptor: [Clinical Trial] explode all trees	140
10	MeSH descriptor: [Feasibility Studies] this term only	6031
11	MeSH descriptor: [Pilot Projects] this term only	19388
12	interven*	404715
13	supplement*	88063
14	biofortif* or (bio NEXT fortif*)	162
15	(fortif* or enrich* or enhanc* or boost*) NEAR/2 (food* or rice or flour or corn or wheat or semolina or pulse* or bread* or sauce* or beverage* or maize or oat* or sugar or salt or oil* or fat* or condiment* or seasoning* or spice* or milk or dairy or juice* or nectar* or cereal* or biscuit* or drink* or diet* or beverage* or meal*)	5498
16	{OR #6-#15}	481662
17	(zinc* or zn*) NEAR/5 deplet*	30
18	#16 OR #17	481669
19	#5 AND #18	4232
20	MeSH descriptor: [Animals] explode all trees	584579
21	MeSH descriptor: [Humans] explode all trees	584493
22	#20 NOT #21	86
23	#19 NOT #22	4232
24	#19 NOT #22 with Cochrane Library publication date from Jan 2007 to present	3445
25	#24 - Cochrane Reviews, Cochrane Protocols, Clinical Trials (not clinical answers, editorials or special collections)	3401

Update search August 2021

Medline

Ovid MEDLINE(R) and Epub Ahead of Print, In-Process, In-Data-Review & Other Non-Indexed Citations and Daily <1946 to August 31, 2021>

1 exp Zinc/ 62511

2 exp Zinc Compounds/ or Zinc Acetate/ 14260

3 (zinc or zn).ti,kf,kw. 67465

4 (zinc or zn).ab. /freq=2 79447

5 or/1-4 131100

6 Dietary Supplements/ 64331

7 exp Biofortification/ 263

8 exp Food, Fortified/ 9720

9 exp clinical trial/ or feasibility studies/ or pilot projects/ 1065670

10	interven*.mp.	1205815

11	supplement*.mp.	377390

12	(biofortif* or bio fortif*).mp.	1561

13	((fortif* or enrich* or enhanc* or boost*) adj2 (food* or rice or flour or corn or wheat or semolina or pulse* or bread* or sauce* or beverage* or maize or oat* or sugar or salt or oil* or fat* or condiment* or seasoning* or spice* or milk or dairy or juice* or nectar* or cereal* or biscuit* or drink* or diet* or beverage* or meal*)).mp.	36486

14	or/6-13 2447745

15	((zinc* or zn*) adj5 deplet*).mp.	1109

16	14 or 15	2448620

17	5 and 16	12580

18	exp animals/ not humans.sh.	4880312

19	17 not 18	9247

20	limit 19 to yr="2020 -Current"	1172

Embase

<1974 to 2021 August 31>

1	(zinc or zn).ti,kw.	77490

2	(zinc or zn).ab. /freq=2	93831

3	1 or 2	123213

4	interven*.mp.	1681988

5	supplement*.mp.	521686

6	(biofortif* or bio fortif*).mp.	1791

7	((fortif* or enrich* or enhanc* or boost*) adj2 (food* or rice or flour or corn or wheat or semolina or pulse* or bread* or sauce* or beverage* or maize or oat* or sugar or salt or oil* or fat* or condiment* or seasoning* or spice* or milk or dairy or juice* or nectar* or cereal* or biscuit* or drink* or diet* or beverage* or meal*)).mp.	36551

8	4 or 5 or 6 or 7	2179493

9	((zinc* or zn*) adj5 deplet*).mp.	1278

10	8 or 9	2180480

11	3 and 10	12900

12	exp animal/ not human.sh.	4971690

13	11 not 12	9062

14	limit 13 to yr="2020 - 2021"	1052

Cochrane Library

Search Name: SR3 Cochrane

Date Run: 02/09/2021 22:51:19

Comment:

ID	Search	Hits
#1	MeSH descriptor: [Zinc] explode all trees	1681
#2	MeSH descriptor: [Zinc Compounds] explode all trees	556
#3	MeSH descriptor: [Zinc Acetate] this term only	27
#4	zinc or zn	9041
#5	{OR #1-#4}	9041
#6	MeSH descriptor: [Dietary Supplements] explode all trees	13460
#7	MeSH descriptor: [Biofortification] explode all trees	4
#8	MeSH descriptor: [Food, Fortified] explode all trees	1474
#9	MeSH descriptor: [Clinical Trial] explode all trees	141
#10	MeSH descriptor: [Feasibility Studies] this term only	6750
#11	MeSH descriptor: [Pilot Projects] this term only	21005
#12	interven*	460387
#13	supplement*	95182
#14	biofortif* or (bio NEXT fortif*)	188
#15	(fortif* or enrich* or enhanc* or boost*) NEAR/2 (food* or rice or flour or corn or wheat or semolina or pulse* or bread* or sauce* or beverage* or maize or oat* or sugar or salt or oil* or fat* or condiment* or seasoning* or spice* or milk or dairy or juice* or nectar* or cereal* or biscuit* or drink* or diet* or beverage* or meal*)	5875
#16	{OR #6-#15}	541960
#17	(zinc* or zn*) NEAR/5 deplet*	31
#18	#16 OR #17	541967
#19	#5 AND #18	4616
#20	MeSH descriptor: [Animals] explode all trees	617483
#21	MeSH descriptor: [Humans] explode all trees	617424
#22	#20 NOT #21	59
#23	#19 NOT #22	4616
#24	#19 NOT #22 with Cochrane Library publication date Between Sep 2020 and Sep 2021, in Trials	361

Update Search July 2022

Medline

Ovid MEDLINE(R) and Epub Ahead of Print, In-Process, In-Data-Review & Other Non-Indexed Citations and Daily <1946 to July 29, 2022>

1 exp Zinc/ 65015

2 exp Zinc Compounds/ or Zinc Acetate/ 15756

3 (zinc or zn).ti,kf,kw. 71996

4 (zinc or zn).ab. /freq=2 84374

5 or/1-4 138694

6 Dietary Supplements/ 70061

7 exp Biofortification/ 330

8 exp Food, Fortified/ 9984

9 exp clinical trial/ or feasibility studies/ or pilot projects/ 1117304

10 interven*.mp. 1312196

11 supplement*.mp. 405790

12 (biofortif* or bio fortif*).mp. 1856

13 ((fortif* or enrich* or enhanc* or boost*) adj2 (food* or rice or flour or corn or wheat or semolina or pulse* or bread* or sauce* or beverage* or maize or oat* or sugar or salt or oil* or fat* or condiment* or seasoning* or spice* or milk or dairy or juice* or nectar* or cereal* or biscuit* or drink* or diet* or beverage* or meal*)).mp. 38973

14 or/6-13 2615565

15 ((zinc* or zn*) adj5 deplet*).mp. 1139

16 14 or 15 2616464

17 5 and 16 13361

18 exp animals/ not humans.sh. 5033212

19 17 not 18 9840

20 ("20210831" or 202109* or 20211* or 2022*).dt,ez,da. 1954105

21 19 and 20 840

Embase

Embase <1974 to 2022 July 29>

1 (zinc or zn).ti,kw. 73927

2 (zinc or zn).ab. /freq=2 98517

3 1 or 2 124325

4 interven*.mp. 1821943

5 supplement*.mp. 556149

6 (biofortif* or bio fortif*).mp. 2068

7 ((fortif* or enrich* or enhanc* or boost*) adj2 (food* or rice or flour or corn or wheat or semolina or pulse* or bread* or sauce* or beverage* or maize or oat* or sugar or salt or oil* or fat* or condiment* or seasoning* or spice* or milk or dairy or juice* or nectar* or cereal* or biscuit* or drink* or diet* or beverage* or meal*)).mp. 38860

8 4 or 5 or 6 or 7 2351172

9 ((zinc* or zn*) adj5 deplet*).mp. 1316

10 8 or 9 2352188

11 3 and 10 13523

12 exp animal/ not human.sh. 5119587

13 11 not 12 9518

14 limit 13 to dc=20210831-20220729 745

Cochrane Library

Search Name: SR3 Cochrane

Date Run: 01/08/2022 08:38:59

Comment:

ID	Search	Hits
#1	MeSH descriptor: [Zinc] explode all trees	1732
#2	MeSH descriptor: [Zinc Compounds] explode all trees	573
#3	MeSH descriptor: [Zinc Acetate] this term only	28
#4	zinc or zn	9395
#5	{OR #1-#4}	9395
#6	MeSH descriptor: [Dietary Supplements] explode all trees	14594
#7	MeSH descriptor: [Biofortification] explode all trees	6
#8	MeSH descriptor: [Food, Fortified] explode all trees	1518
#9	MeSH descriptor: [Clinical Trial] explode all trees	141
#10	MeSH descriptor: [Feasibility Studies] this term only	7370
#11	MeSH descriptor: [Pilot Projects] this term only	22663

#12 interven* 497185

#13 supplement* 99794

#14 biofortif* or (bio NEXT fortif*) 202

#15 (fortif* or enrich* or enhanc* or boost*) NEAR/2 (food* or rice or flour or corn or wheat or semolina or pulse* or bread* or sauce* or beverage* or maize or oat* or sugar or salt or oil* or fat* or condiment* or seasoning* or spice* or milk or dairy or juice* or nectar* or cereal* or biscuit* or drink* or diet* or beverage* or meal*) 6046

#16 {OR #6-#15} 582065

#17 (zinc* or zn*) NEAR/5 deplet* 31

#18 #16 OR #17 582072

#19 #5 AND #18 4831

#20 MeSH descriptor: [Animals] explode all trees 652285

#21 MeSH descriptor: [Humans] explode all trees 652263

#22 #20 NOT #21 22

#23 #19 NOT #22 4831

#24 #19 NOT #22 with Cochrane Library publication date Between Jul 2022 and Aug 2022, in Trials 10

#25 #19 NOT #22 with Cochrane Library publication date Between Jul 2021 and Aug 2022 328

**Note: From those 328

Cochrane reviews: 24

Cochrane protocols: 6

Trials: 296

Clinical Answers:2

Only trials N=296 were downloaded into Endnote.
