# Supplementary material for: Methods of Assessment of Zinc Status in Humans: An Updated Review and Meta-analysis
Source: Nutr Rev. 2024 Jun 25;83(3):e778–800. doi: 10.1093/nutrit/nuae072 (PMC11819495; doi:10.1093/nutrit/nuae072)
Supplement: nuae072_Supplementary_Data [file nuae072_supplementary_data.zip › nuae072_Supplementary_Data/Supplemental Information 2.pdf]

Supplementary file 2. Risk of Bias and GRADE assessments

Note: Reference numbers refer to the reference list in the review

Table of contents

Risk of Bias Summary

Randomized control trials included in the meta-analysis 1

Non-Randomized studies included in the meta-analysis 2

Randomized control trials included in the narrative analysis 3

Non-Randomized studies included in the narrative analysis 4

Risk of Bias Graphs

Randomized control trials included in the meta-analysis 5

Non-randomized studies included in the meta-analysis........ 5

Randomized control trials included in the narrative analysis 6

Non-randomized studies included in the narrative analysis 6

GRADE

Grade Evidence table: Serum/Plasma zinc, controlled trials (mmol/L) 7

Grade Evidence table: Serum/Plasma zinc, before and after studies (mmol/L) 7

Grade Evidence table: Urinary zinc 26

Grade Evidence table: Alkaline phosphatase (ALP; U/L) 36

Grade Evidence table: Other biomarkers 40

Methods of assessment of zinc status in humans: an updated review and meta-analysis.
Ceballos-Rasgado, et al.

Risk of Bias Summary

Randomized control trials included in the meta-analysis

Study	Randomisation process	Deviations from the intended interventions	Missing outcome data	Measurement of the outcome	Selection of the reported result	Overall
Abdollahi et al. (2019)[35]	!	+	+	+	+	!
Abdulla and Suck. (1998)[36]	!	+	-	+	+	-
Ahmadi et al. (2020)[39]	+	+	+	+	+	+
Attia et al. (2022)[41]	!	+	-	+	+	-
Ayatollahi et al. (2022)[42]	+	+	+	+	+	+
Ba Lo et al. (2011)[43]	+	+	+	+	+	+
Bao et al. (2010)[45]	+	+	+	+	+	+
Barrie et al. (1987)[46]	+	+	+	+	+	+
Berger et al. (2015)[48]	+	+	+	+	+	+
Black et al. (1988)[32]	-	+	-	+	+	-
Bogale et al. (2015)[51]	+	+	+	+	+	+
Bogden et al. (1988)[50]	!	+	+	+	+	!
Brown et al. (2007)[52]	+	+	+	+	+	+
Crouse et al. (1984)[55]	!	+	+	+	+	!
Demetree et al. (1980)[58]	!	+	+	+	+	!
DiSilvestro et al. (2015)[59]	!	+	+	+	+	!
Fahmida et al. (2007)[64]	+	+	+	+	+	+
Fernandes de Oliveira et al. (2009)[66]	+	+	+	+	+	+
Field et al. (1987)[67]	!	!	-	+	+	-
Gatto and Samman. (1995)[70]	+	-	+	!	+	-
Gomes Dantas Lopes et al. (2015)[71]	!	+	+	+	+	!
Gülsan et al. (2013)[72]	!	!	-	+	+	-
Heckmann et al. (2005)[75]	+	+	+	+	+	+
Hininger-Favier et al. (2007)[31]	!	+	+	+	-	-
Hunt et al. (1985)[77]	!	+	-	-	+	-
Islam et al. (2022)[79]	+	+	+	+	+	+
Jafari et al. (2020)[80]	!	+	+	+	+	!
Kaseb et al. (2013)[82]	+	+	+	+	+	+

Study	Randomisation process	Deviations from the intended interventions	Missing outcome data	Measurement of the outcome	Selection of the reported result	Overall
Khorsandi et al. (2019)[83]	+	!	+	+	+	!
Kim et al. (2014)[84]	!	-	-	+	+	-
Long et al. (2022) [88]	+	+	+	+	+	+
Massih et al. (2021)[92]	+	+	+	+	+	+
Mazaheri Nia et al. (2021)[93]	!	+	+	+	+	!
Medeiros et al. (1987)[30]	!	+	+	+	+	!
Mesdaghinia et al. (2019)[94]	+	+	+	+	+	+
Mujica-Coopman et al. (2015)[96]	+	+	+	+	+	+
Payahoo et al. (2013)[100]	!	+	+	+	+	!
Prasad et al. (2007)[104]	!	+	+	+	+	!
Rohmawati et al. (2021)[105]	+	+	+	+	+	+
Samman and Roberts. (1987)[107]	+	-	+	+	!	-
Shaaban et al. (2005)[108]	!	!	-	+	+	-
Solati et al. (2015)[109]	+	+	+	+	+	+
Stur et al. (1996)[111]	!	+	-	+	+	-
Sullivan et al. (1997)[112]	!	+	+	+	+	+
Sullivan et al. (1998)[113]	!	+	-	+	+	-
Surono et al. (2014)[114]	+	+	+	+	+	+
Swanson et al. (1988)[115]	!	-	-	+	+	-
Tamura et al. (1996)[117]	!	+	+	+	!	!
Tamura et al. (2001)[118]	!	+	+	+	!	!
Thomas et al. (1992)[119]	!	+	+	+	+	!
Weismann et al. (1977)[122]	!	+	-	+	+	-
Wessels et al. (2010)[123]	+	+	+	+	+	+
Wessels et al. (2012)[124]	+	+	+	+	+	+
Wessells et al. (2021)[13]	!	+	+	+	+	!
Yalda and Ibrahiem. (2010)[126]	!	+	+	+	+	!
Yosaee et al. (2020)[127]	+	+	+	+	+	+

Key

+	Low risk of bias
!	Unclear risk of bias
-	High risk of bias

Figure 1. Risk of Bias summary of all randomized control trials included in the meta-analysis, shown as the authors judgment for each RoB2 category for each study included.

Non-Randomized studies included in the meta-analysis

Study	Confounding	Selection of participants into the study	Classification of interventions	Deviations from intended interventions	Missing outcome data	Measurement of the outcome	Selection of reported results	Overall
Abdulla and Svensson1. (1979)[37]	!	!	+	+	!	+	+	!
Abdulla and Svensson2. (1979)[37]	!	!	+	+	!	+	+	!
Allan et al. (2000)[40]	!	!	+	+	!	+	+	!
Bales et al. (1994)[44]	-	!	+	+	+	+	+	-
Cesur et al. (2009)[53]	!	-	+	+	-	!	+	-
Chung et al. (2008)[54]	!	!	+	+	+	+	+	!
Deguchi et al. (2019)[57]	-	-	-	-	-	-	-	-
Donangelo et al. (2002)[60]	-	!	+	+	+	+	+	-
Duchateau et al. (1981)[61]	-	!	+	!	+	+	+	-
Eskici et al. (2017)[62]	-	!	+	+	+	+	+	-
Eskici et al. (2016)[63]	-	!	+	+	+	+	+	-
Farrell et al. (2011)[65]	-	!	+	+	+	+	+	-
Fischer et al. (1984)[68]	-	!	+	!	-	+	+	-
Freeland-Graves et al. (1981)[69]	-	!	+	!	!	+	!	-
Gupta et al. (1998)[73]	!	!	+	+	!	+	!	!
Hollingsworth et al. (1987)[76]	-	!	+	!	+	+	+	-
Leite et al. (2009)[86]	!	!	+	+	+	+	+	!
Lowe et al. (2004)[87]	-	!	+	-	-	+	+	-
Lukaski et al. (1984)[89]	!	!	+	+	+	+	+	!
Marques, 2011)[91]	-	!	+	+	+	+	+	-
Milne et al. (1987)[95]	-	!	+	+	+	+	+	-
Pachotikarn et al. (1985)[98]	-	-	+	+	+	+	+	-
Palin et al. (1979)[99]	-	!	-	!	+	+	+	-
Peretz et al. (1993)[101]	!	!	+	+	+	+	+	!
Pinna et al. (2002)[102]	!	!	+	+	+	+	+	!
Prasad et al. (1996)[103]	-	!	+	+	-	+	+	-
Ruz et al. (1992)[106]	!	!	+	+	+	+	+	!
Song et al. (2009)[110]	!	!	+	+	+	+	+	!
Takacs et al. (2020)[116]	-	!	+	+	+	+	+	-
Vale et al. (2014)[120]	!	!	+	+	+	+	+	!
Yadrick et al. (1989)[125]	!	!	+	!	!	+	+	!

Figure 2. Risk of Bias summary of all non-randomized studies included in the meta-analysis, shown as the authors judgment for each ROBINS-I (Risk of bias in non-randomized studies of interventions) category for each study included.

Randomized control trials included in the narrative analysis

Study	Randomisation process	Deviations from the intended interventions	Missing outcome data	Measurement of the outcome	Selection of the reported result	Overall
Abdollahi et al. (2019)[35]	!	+	+	+	+	!
Abdulla and Suck. (1998)[36]	!	+	-	+	+	-
Adriani & Wirjatmadi. (2014)[38]	+	+	+	+	+	+
Ahmadi et al. (2020)[39]	+	+	+	+	+	+
Attia et al. (2022)[41]	!	+	-	+	+	-
Ayatollahi et al. (2022)[42]	+	+	+	+	+	+
Ba Lo et al. (2011)[43]	+	+	+	+	+	+
Bao et al. (2010)[45]	+	+	+	+	+	+
Barrie et al. (1987)[46]	+	+	+	+	+	+
Becquey et al. (2016)[47]	+	+	+	+	+	+
Berger et al. (2015)[48]	+	+	+	+	+	+
Bertinato et al. (2012)[49]	+	+	+	+	+	+
Black et al. (1988)[32]	-	+	-	+	+	-
Bogden et al. (1988)[50]	+	+	+	+	+	+
Bogale et al. (2015)[51]	!	+	+	+	+	!
Brown et al. (2007)[52]	+	+	+	+	+	+
Crouse et al. (1984)[55]	!	+	+	+	+	!
de Brito et al. (2014)[56]	!	+	+	+	+	!
Demetree et al. (1980)[58]	!	+	+	+	+	!
DiSilvestro et al. (2015)[59]	!	+	+	+	+	!
Fahmida et al. (2007)[64]	+	+	+	+	+	+
Fernandes de Oliveira et al. (2009)[66]	+	+	+	+	+	+
Field et al. (1987)[67]	!	!	-	+	+	-
Gatto and Samman. (1995)[70]	+	-	+	!	+	-
Gomes Dantas Lopes et al. (2015)[71]	!	+	+	+	+	!
Gülsan et al. (2013)[72]	!	!	-	+	+	-
Heckmann et al. (2005)[75]	+	+	+	+	+	+
Hininger-Favier et al. (2007)[31]	!	+	+	+	-	-
Hodikson et al. (2007)[29]	+	+	+	+	+	+
Hunt et al. (1985)[77]	!	+	-	-	+	-
Islam et al. (2016)[78]	+	+	+	+	+	+
Islam et al. (2022)[79]	+	+	+	+	+	+

Study	Randomisation process	Deviations from the intended interventions	Missing outcome data	Measurement of the outcome	Selection of the reported result	Overall
Jafari et al. (2020)[80]	!	+	+	+	+	!
Joray et al. (2014)[81]	+	+	+	+	+	+
Kaseb et al. (2013)[82]	+	+	+	+	+	+
Khorsandi et al. (2019)[83]	+	!	+	+	+	!
Kim et al. (2014)[84]	!	-	-	+	+	-
Long et al. (2022) [88]	+	+	+	+	+	+
Massih et al. (2021)[92]	+	+	+	+	+	+
Mazaheri Nia et al. (2021)[93]	!	+	+	+	+	!
Medeiros et al. (1987)[30]	+	+	+	+	+	+
Mesdaghinia et al. (2019)[94]	!	+	+	+	+	!
Mujica-Coopman et al. (2015)[96]	+	+	+	+	+	+
Noh et al. (2014)[97]	+	+	+	+	+	+
Payahoo et al. (2013)[100]	!	+	+	+	+	!
Prasad et al. (2007)[104]	!	+	+	+	+	!
Rohmawati et al. (2021)[105]	+	+	+	+	+	+
Samman and Roberts. (1987)[107]	+	-	+	+	!	-
Shaaban et al. (2005)[108]	!	!	-	+	+	-
Solati et al. (2015)[109]	+	+	+	+	+	+
Stur et al. (1996)[111]	!	+	-	+	+	-
Sullivan et al. (1997)[112]	!	+	+	+	+	+
Sullivan et al. (1998)[113]	!	+	-	+	+	-
Surono et al. (2014)[114]	+	+	+	+	+	+
Swanson et al. (1988)[115]	!	-	-	+	+	-
Tamura et al. (1996)[117]	!	+	+	+	!	!
Tamura et al. (2001)[118]	!	+	+	+	!	!
Thomas et al. (1992)[119]	!	+	+	+	+	!
Wang et al. (2021)[121]	!	+	+	+	+	!
Weismann et al. (1977)[122]	!	+	-	+	+	-
Wessells et al. (2010)[123]	+	+	+	+	+	+
Wessells et al. (2012)[124]	+	+	+	+	+	+
Wessells et al. (2021)[13]	!	+	+	+	+	!
Yalda and Ibrahiem. (2010)[126]	!	+	+	+	+	!
Yosaee et al. (2020)[127]	+	+	+	+	+	+

Figure 3. Risk of Bias summary of all randomized control trials included in the narrative analysis, shown as the authors judgment for each RoB2 category for each study included.

Non-Randomized studies included in the narrative analysis

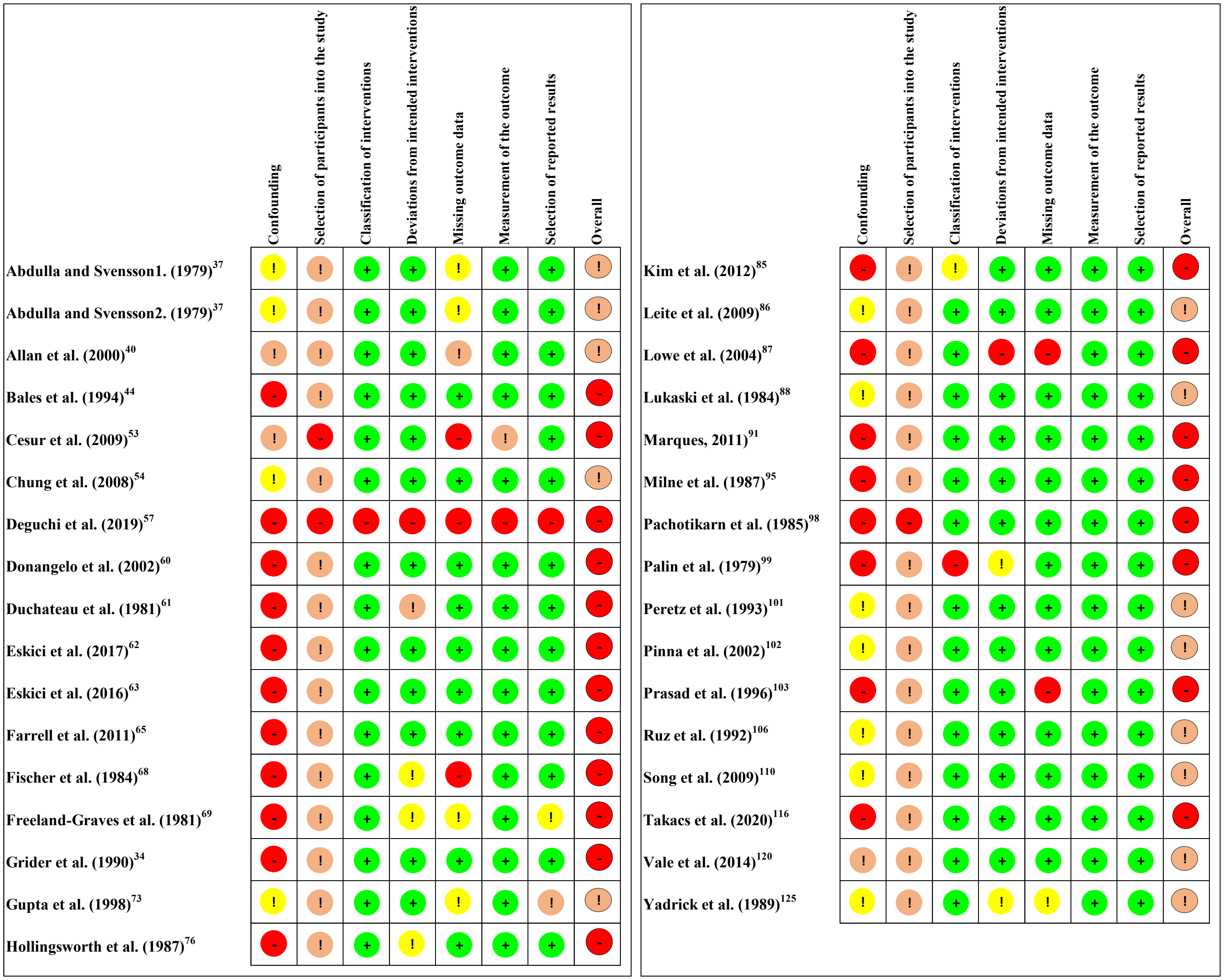

Figure 4. Risk of Bias summary of all non-randomized studies included in the narrative analysis, shown as the authors judgment for each ROBINS-I (Risk of bias in non-randomized studies of interventions) category for each study included.

Risk of Bias Graphs

Randomized control trials included in the meta-analysis

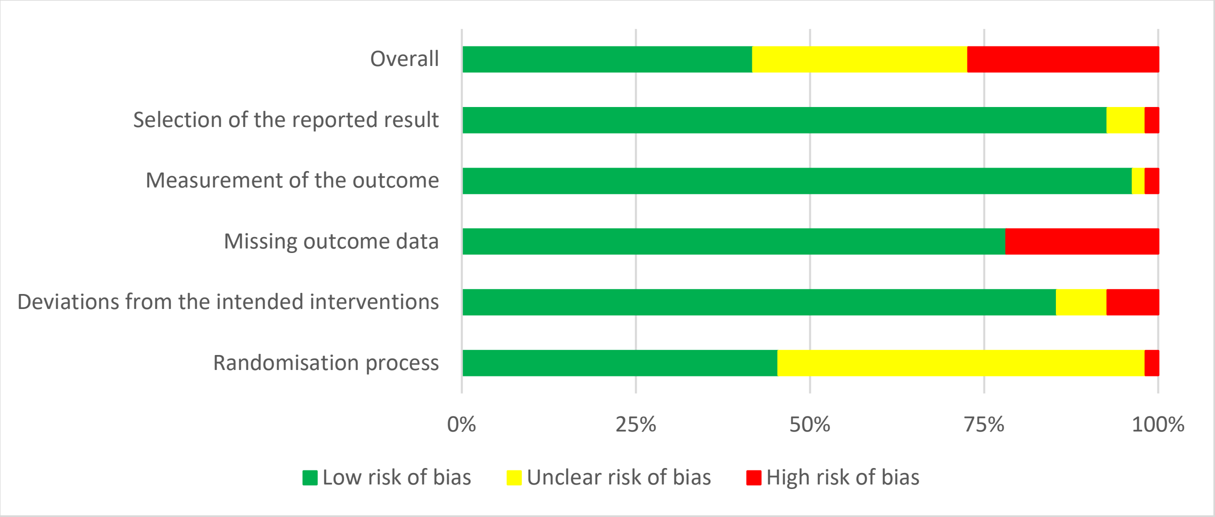

Figure 5. Risk of Bias graph of all randomized control trials included in the meta-analysis. Each risk of bias category is presented as a percentage of all the studies included in the meta-analysis, the overall bias is calculated as per the Cochrane RoB 2 algorithm (low risk if all categories are low risk, unclear risk if some categories have some concerns, and high risk if many categories have some concerns or if one or more categories has high risk).

Non-randomized studies included in the meta-analysis

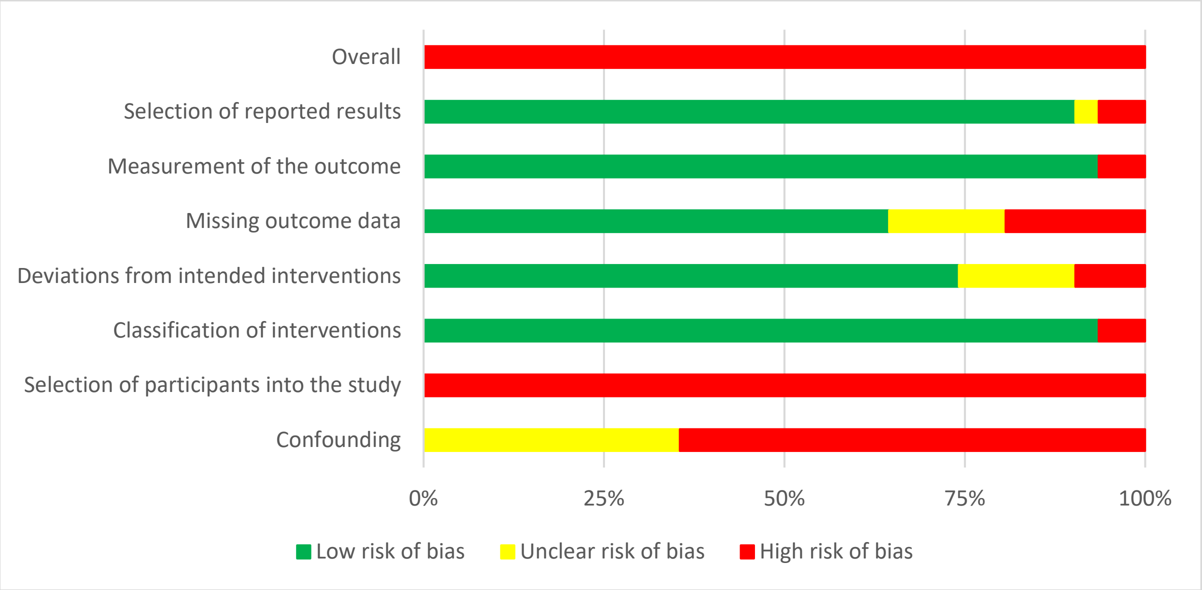

Figure 6. Risk of Bias graph of all non-randomized studies included in the meta-analysis. Each risk of bias category is presented as a percentage of all the studies included in the meta-analysis, the overall bias is calculated based on the same principles as the Cochrane RoB 2 algorithm (low risk if all categories are low risk, unclear risk if some categories have some concerns, and high risk if many categories have some concerns or if one or more categories has high risk).

Randomized control trials included in the narrative analysis

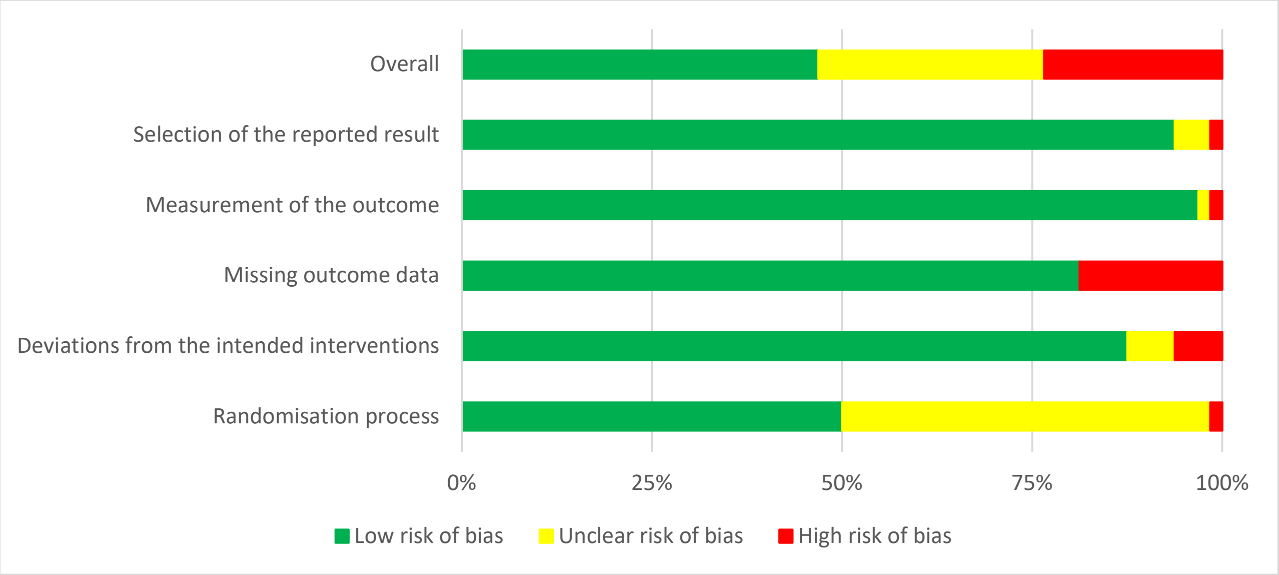

Figure 7. Risk of Bias graph of all randomized control trials included in the narrative analysis. Each risk of bias category is presented as a percentage of all the studies included in the narrative analysis, the overall bias is calculated as per the Cochrane RoB 2 algorithm (low risk if all categories are low risk, unclear risk if some categories have some concerns, and high risk if many categories have some concerns or if one or more categories has high risk).

Non-randomized studies included in the narrative analysis

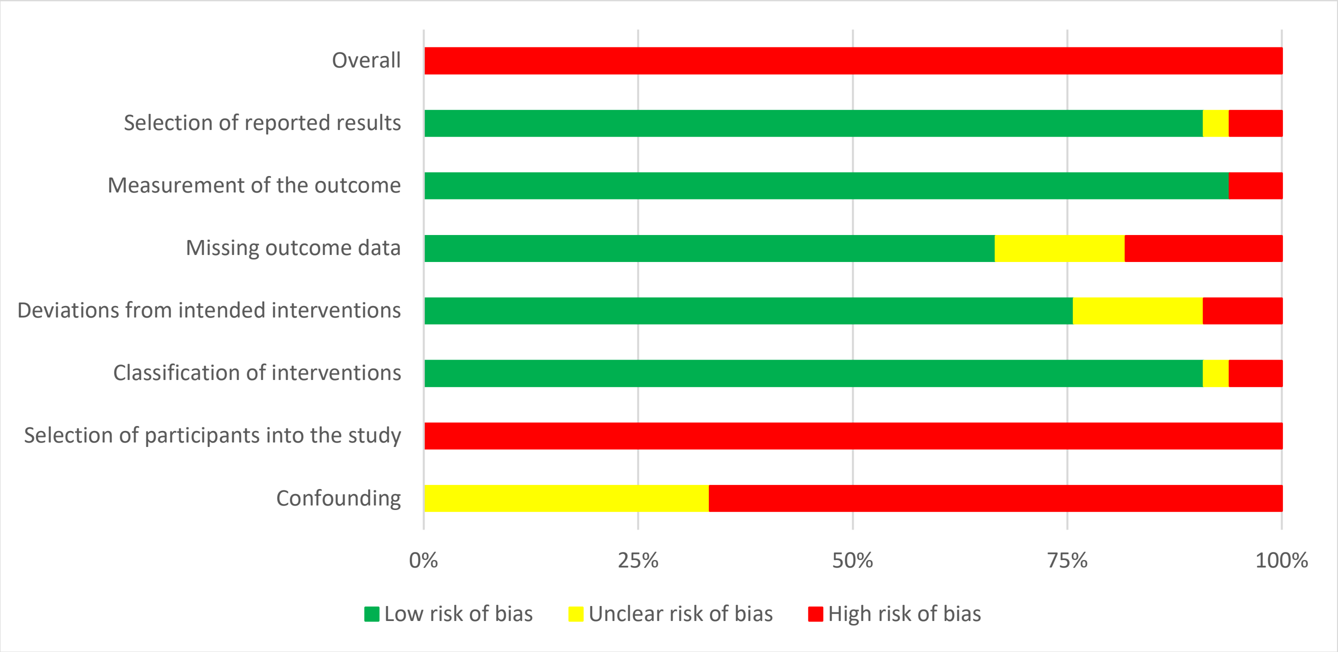

Figure 8. Risk of Bias graph of all non-randomized studies included in the narrative analysis. Each risk of bias category is presented as a percentage of all the studies included in the narrative analysis, the overall bias is calculated based on the same principles as the Cochrane RoB 2 algorithm (low risk if all categories are low risk, unclear risk if some categories have some concerns, and high risk if many categories have some concerns or if one or more categories has high risk).

GRADE

Grade Evidence table: Serum/Plasma zinc, controlled trials (mmol/L)

Certainty assessment							№ of patients		Effect		Certainty	Importance
№ of studies	Study design	Risk of bias	Inconsistency	Indirectness	Imprecision	Other considerations	Zinc supplement	Control	Relative (95% CI)	Absolute (95% CI)		
**Serum/plasma zinc, controlled trials by study design: All studies**												
48	RCTs / NRS	very serious[a]	serious[b]	not serious	serious[c]	publication bias strongly suspected[d]	2223	2093	-	MD **2.18 mmol/L higher** (1.74 higher to 2.61 higher)	⊕◯◯◯ Very low	CRITICAL
**Serum/plasma zinc, controlled trials by study design: RCTs only**												
45	RCTs	very serious[e]	serious[b]	not serious	serious[c]	publication bias strongly suspected[d]	2196	2065	-	MD **1.97 mmol/L higher** (1.55 higher to 2.4 higher)	⊕◯◯◯ Very low	CRITICAL
**Serum/plasma zinc, controlled trials by study design: Non-randomised trials**												
3	NRS	very serious[f]	serious[b]	not serious	serious[c]	none	27	28	-	MD **5.41 mmol/L higher** (2.42 lower to 13.23 higher)	⊕◯◯◯ Very low	IMPORTANT
**Serum/plasma zinc, controlled trials by sex: Males**												
8	RCTs / NRS	very serious[g]	not serious	not serious	serious[c]	publication bias strongly suspected[d]	138	114	-	MD **1.67 mmol/L higher** (1.34 higher to 2.01 higher)	⊕◯◯◯ Very low	IMPORTANT

Certainty assessment							№ of patients		Effect		Certainty	Importance
№ of studies	Study design	Risk of bias	Inconsistency	Indirectness	Imprecision	Other considerations	Zinc supplement	Control	Relative (95% CI)	Absolute (95% CI)		
**Serum/plasma zinc, controlled trials by sex: Female**												
13	RCTs	serious[h]	serious[i]	not serious	serious[c]	publication bias strongly suspected[d]	516	502	-	MD **1.58 mmol/L higher** (0.86 higher to 2.29 higher)	⊕◯◯◯ Very low	IMPORTANT
**Serum/plasma zinc, controlled trials by sex: Mixed male and female**												
26	RCTs / NRS	serious[j]	serious[b]	not serious	serious[c]	publication bias strongly suspected[d]	1569	1477	-	MD **2.39 mmol/L higher** (1.84 higher to 2.94 higher)	⊕◯◯◯ Very low	IMPORTANT
**Serum/plasma zinc, controlled trials by population: Infants (0-12 months)**												
4	RCTs	not serious	serious[k]	not serious	serious[l]	none	157	180	-	MD **2.72 mmol/L higher** (1.68 higher to 3.75 higher)	⊕⊕◯◯ Low	IMPORTANT
**Serum/plasma zinc, controlled trials by population: Children and adolescents**												
11	RCTs	serious[m]	serious[b]	not serious	serious[c]	publication bias strongly suspected[d]	882	907	-	MD **0.96 mmol/L higher** (0.07 higher to 1.86 higher)	⊕◯◯◯ Very low	IMPORTANT
**Serum/plasma zinc, controlled trials by population: Pregnancy and lactation**												
3	RCTs	serious[n]	serious[i]	not serious	serious[l]	none	155	151	-	MD **1.3 mmol/L higher** (0.09 higher to 2.7 higher)	⊕◯◯◯ Very low	IMPORTANT

Certainty assessment							№ of patients		Effect		Certainty	Importance
№ of studies	Study design	Risk of bias	Inconsistency	Indirectness	Imprecision	Other considerations	Zinc supplement	Control	Relative (95% CI)	Absolute (95% CI)		
**Serum/plasma zinc, controlled trials by population: Adults**												
23	RCTs / NRS	serious[o]	serious[p]	not serious	serious[c]	publication bias strongly suspected[d]	508	488	-	MD **2.65 mmol/L higher** (1.8 higher to 3.5 higher)	⨁◯◯◯ Very low	IMPORTANT
**Serum/plasma zinc, controlled trials by population: Postmenopausal women**												
1	RCTs	not serious	not serious	not serious	not serious	none	57	55	-	MD **4.64 mmol/L higher** (3.93 higher to 5.35 higher)	⨁⨁⨁⨁ High	IMPORTANT
**Serum/plasma zinc, controlled trials by population: Elderly**												
4	RCTs	very serious[q]	not serious	not serious	not serious	none	147	120	-	MD **3.54 mmol/L higher** (2.8 higher to 4.28 higher)	⨁⨁◯◯ Low	IMPORTANT
**Serum/plasma zinc, controlled trials by status at baseline: Normal serum/plasma zinc status at baseline**												
44	RCTs / NRS	serious[r]	serious[b]	not serious	serious[c]	publication bias strongly suspected[d]	1976	1868	-	MD **2.15 mmol/L higher** (1.69 higher to 2.6 higher)	⨁◯◯◯ Very low	IMPORTANT
**Serum/plasma zinc, controlled trials by status at baseline: Low serum/plasma zinc status at baseline**												
4	RCTs	serious[s]	serious[t]	not serious	serious[c]	none	247	225	-	MD **2.46 mmol/L higher** (0.9 higher to 4.01 higher)	⨁◯◯◯ Very low	IMPORTANT

Certainty assessment							№ of patients		Effect		Certainty	Importance
**№ of studies**	**Study design**	**Risk of bias**	**Inconsistency**	**Indirectness**	**Imprecision**	**Other considerations**	**Zinc supplement**	**Control**	**Relative (95% CI)**	**Absolute (95% CI)**		
**Serum/plasma zinc, controlled trials by dose: Supplement 1-2.9 mg Zn/d**												
2	RCTs	serious[u]	not serious	not serious	not serious	none	87	87	-	MD **0.58 mmol/L higher** (0.37 lower to 1.54 higher)	⊕⊕⊕◯ Moderate	IMPORTANT
**Serum/plasma zinc, controlled trials by dose: Supplementation 3 to 15 mg Zn/d**												
15	RCTs	serious[v]	serious[b]	not serious	serious[b]	publication bias strongly suspected[d]	1156	1121	-	MD **2.05 mmol/L higher** (1.43 higher to 2.67 higher)	⊕◯◯◯ Very low	IMPORTANT
**Serum/plasma zinc, controlled trials by dose: Supplementation 16 to 25 mg Zn/d**												
10	RCTs / NRS	serious[w]	serious[i]	not serious	serious[c]	publication bias strongly suspected[d]	360	347	-	MD **1.55 mmol/L higher** (0.68 higher to 2.42 higher)	⊕◯◯◯ Very low	IMPORTANT
**Serum/plasma zinc, controlled trials by dose: Supplementation 26 to 50 mg Zn/d**												
19	RCTs / NRS	serious[x]	serious[y]	not serious	serious[c]	publication bias strongly suspected[d]	544	484	-	MD **1.9 mmol/L higher** (1.38 higher to 2.42 higher)	⊕◯◯◯ Very low	IMPORTANT
**Serum/plasma zinc, controlled trials by dose: Supplementation 51 to 100 mg Zn/d**												
4	RCTs	very serious[z]	not serious	not serious	serious[c]	none	56	37	-	MD **4.16 mmol/L higher** (2.92 higher to 5.41 higher)	⊕◯◯◯ Very low	IMPORTANT

Certainty assessment							№ of patients		Effect		Certainty	Importance
№ of studies	Study design	Risk of bias	Inconsistency	Indirectness	Imprecision	Other considerations	Zinc supplement	Control	Relative (95% CI)	Absolute (95% CI)		
**Serum/plasma zinc, controlled trials by dose: Supplementation 101 to 151 mg Zn/d**												
2	RCTs / NRS	very serious[aa]	very serious[ab]	not serious	serious[c]	none	20	17	-	MD **7.55 mmol/L higher** (1.7 lower to 16.8 higher)	⊕◯◯◯ Very low	IMPORTANT
**Serum/plasma zinc, controlled trials by supplement type: Zinc sulphate**												
29	RCTs / NRS	serious[ac]	serious[b]	not serious	serious[c]	publication bias strongly suspected[d]	1526	1555	-	MD **1.96 mmol/L higher** (1.38 higher to 2.54 higher)	⊕◯◯◯ Very low	IMPORTANT
**Serum/plasma zinc, controlled trials by supplement type: Zinc gluconate**												
17	RCTs / NRS	serious[ad]	serious[ae]	not serious	serious[c]	publication bias strongly suspected[d]	612	485	-	MD **2.17 mmol/L higher** (1.55 higher to 2.8 higher)	⊕◯◯◯ Very low	IMPORTANT
**Serum/plasma zinc, controlled trials by supplement type: Zinc acetate**												
2	RCTs / NRS	very serious[af]	not serious	not serious	not serious	none	85	53	-	MD **4.05 mmol/L higher** (3.2 higher to 4.9 higher)	⊕⊕◯◯ Low	IMPORTANT

CI: confidence interval; **MD:** mean difference; **RCT**: randomized control trial; **NRS**: non-randomized studies

Explanations

a. 48 studied included in the analysis, 45 RCTs and 3 NRS. RCTs - One had high risk of bias and 22 had unclear risk of bias in the randomisation process (selection bias), two had high risk of bias in deviations from the intended interventions (performance bias), eight had high risk of bias in

missing outcome data (attrition bias), one high risk of bias in measurement of the outcome (detection bias), and one has high risk of bias in selection of the reported result (selective outcome reporting bias). NRS - Two had high risk of bias due to confounding, three had high risk of bias in selection of participants into the study, one had high risk of bias in classification of intervention (selection bias), two had unclear risk of bias in deviations from intended interventions (performance bias), and one had high risk of bias and one had unclear risk of bias in missing outcome data (attrition bias). Overall, 14 had unclear risk of bias and 12 had high risk of bias.

b. Wide difference in point estimates, considerable heterogeneity, I^2 >95%.

c. Small number of events, wide confidence intervals including appreciable benefit and harm.

d. Publication bias suspected because of asymmetrical funnel plot.

e. 45 RCTs included in the analysis. One had high risk of bias and 22 had unclear risk of bias in the randomisation process (selection bias), two had high risk of bias in deviations from the intended interventions (performance bias), eight had high risk of bias in missing outcome data (attrition bias), one had high risk of bias in measurement of the outcome (detection bias), and one had high risk of bias in selection of the reported result (selective outcome reporting bias). Overall, 14 had unclear risk of bias and nine had high risk of bias.

f. Three studies included in the analysis. Two had high risk of bias due to confounding, three had high risk of bias in selection of participants into the study, one had high risk of bias in classification of intervention (selection bias), two had unclear risk of bias in deviations from intended interventions (performance bias), and one had high risk of bias and one had unclear risk of bias in missing outcome data (attrition bias). Overall, three had high risk of bias.

g. Eight studies were included in the analysis, seven RCTs and one NRS. RCTs – One had high risk of bias and four had unclear risk of bias in the randomisation process (selection bias), two had high risk of bias in missing outcome data (attrition bias). NRS – High risk of bias due to confounding and selection of participants into the study (selection bias), unclear risk of bias in deviations from the intended interventions (performance bias), and high risk of bias in missing outcome data (attrition bias). Overall, 3 had high risk of bias and two had unclear risk of bias.

h. 13 RCTs were included in the analysis. Seven had unclear risk of bias in the randomisation process (selection bias), one had high risk of bias in deviations from the intended interventions (performance bias), two had high risk of bias in missing outcome data (attrition bias), and one had high risk of bias in measurement of the outcome (detection bias). Overall, two had high risk of bias and five had unclear risk of bias.

i. Considerable heterogeneity, I^2>95%.

j. 27 studies included in the analysis, 25 RCTs and two NRS. RCTs – 11 had unclear risk of bias in randomisation process (selection bias), one had high risk of bias in deviations from the intended interventions (performance bias), four had high risk of bias in missing outcome data (attrition bias), and one had high risk of bias in the selection of reported results (selective outcome reporting bias). NRS – One had high risk and one had unclear risk of confounding, two had high risk of bias in selection of participants into the study and one had high risk of bias in classification of interventions

(selection bias), one had unclear risk of bias in deviations from intended interventions (performance bias), and one had unclear risk of missing outcome data (attrition bias). Overall, seven had unclear risk of bias and seven had high risk of bias.

k. Considerable heterogeneity I^2 >90%.

l. Small sample size and small number of events.

m. 11 RCTs included in analysis. Two had high risk in missing outcome data (attrition bias), one high risk in measurement of the outcome (detection bias). Overall, two studies had high risk of bias.

n. 3 RCTs included in the analysis. Two had unclear risk of bias due to the randomisation process (selection bias), and one had unclear risk of bias in selection of the reported result (selective outcome reporting bias). Overall, two studies had unclear risk of bias.

o. 23 studies included in the analysis, 21 RCTs and two NRS. RCTs - One had high risk of bias and 10 had unclear risk of bias in the randomisation process (selection bias), one had high risk of bias in deviations from intended interventions (performance bias), and four had high risk of bias in missing outcome data (attrition bias). NRS - One had high risk of bias and one had unclear risk of bias in confounding, two had high risk of bias in selection of participants into the study (selection bias), one had unclear risk of bias in deviations from intended interventions (performance bias), and one had high risk of bias and one had unclear risk of bias in missing outcome data (attrition bias). Overall, six had high risk of bias and seven had unclear risk of bias.

p. Wide difference in point estimates, considerable heterogeneity, I^2 >90%.

q. Four RCTs included in the analysis. Four had unclear risk of bias for randomisation process (selection bias), one had high risk of bias in deviations from intended interventions (performance bias), and two had high risk of bias in missing outcome data (attrition bias). Overall, two had high risk of bias and two had unclear rick of bias.

r. 44 studies included in analysis, 41 RCTs, three NRS. RCTs – One had high risk of bias and 20 had unclear risk of bias in the randomisation process (selection bias), two had high risk of bias in deviations from intended interventions (performance bias), eight had high risk of bias in missing outcome data (attrition bias), one had high risk of bias in measurement of the outcome (detection bias), and one had high risk of bias in selection of the reported result (selective outcome reporting bias). NRS – Two had high risk of bias and one had unclear risk of bias in confounding, three had high risk of bias in selection of participants into the study, and one had high risk of bias in classification intervention (selection bias), two had unclear risk of bias in deviations from intended interventions (performance bias), and one had high risk of bias in missing outcome data (attrition bias). Overall, 12 had unclear risk of bias and 12 had high risk of bias.

s. Four RCTs included in the analysis. Two had unclear risk of bias in the randomisation process (selection bias). Overall, two studies had unclear risk of bias.

t. Considerable heterogeneity, I^2 >85%.

u. Two RCTs analysed. One had unclear risk of bias in randomisation process (selection bias), deviations from the intended interventions (performance bias), and high risk of bias in missing outcome data (attrition bias). Overall, one had high risk of bias.

v. 15 RCTs analysed. One had high risk of bias in selection of the reported result (selective outcome reporting bias). Overall, one had high risk of bias.

w. Ten studies analysed, nine RCT and one NRS. RCTs- One had high risk of bias in missing outcome data (attrition bias), and one high risk of bias in measurement of the outcome (detection bias). NRS - High risk of bias in confounding, classification intervention, and selection of participants into the study (selection bias), unclear risk of bias in deviations from intended interventions (performance bias). Overall, two had high risk of bias.

x. 19 studies analysed, 18 RCTs and one NRS. RCTs – 11 had unclear risk of bias and one high risk of bias in randomisation process (selection bias), two had high risk of bias in deviations from the intended interventions (performance bias), five had high risk of bias in missing outcome data (attrition bias), and one had high risk of bias in selection of the reported results (selective outcome reporting bias). NRS - high risk of bias in confounding and selection of participants into the study (selection bias), unclear risk of bias in deviations from the intended interventions (performance bias), and high risk of bias in missing outcome data (attrition bias). Overall, six studies had high risk of bias and six studies unclear risk of bias.

y. Considerable heterogeneity, I^2>75%.

z. Four RCTs analysed. One had high risk of bias and three had unclear risk of bias in randomisation process (selection bias), one had high risk of bias in missing outcome data (attrition bias). Overall, one had high risk of bias, three had unclear rick of bias.

aa. Two studies were analysed, one RCT and one NRS. RCT - Unclear risk of bias randomisation process (selection bias), and high risk of bias in missing outcome data (attrition bias). NRS - Unclear risk of bias in confounding and high risk of bias in deviations from the intended interventions (selection bias), unclear risk of bias in missing outcome data (attrition bias). Overall, two had high risk of bias.

ab. Wide difference in point estimates, confidence intervals do not overlap, considerable heterogeneity, I^2 >95%.

ac. 29 studies analysed, 27 RCTs and two NRS. RCTs – Four had high risk of bias in missing outcome data (attrition bias), one had high risk of bias in measurement of the outcome (detection bias). NRS – One had high risk of bias and one had unclear risk of bias in confounding, two had high risk of bias in selection of participants into the study, and one had had high risk of bias in classification of interventions (selection bias), one had unclear risk of bias in deviations from the intended interventions (performance bias), and one had unclear risk of bias in missing outcome data (attrition bias). Overall, seven had unclear risk of bias and six had high risk of bias.

ad. 17 studies analysed, 16RCT and one NRS. RCTs - One had high risk of bias and 10 had unclear risk of bias in the randomisation process (selection bias), one had high risk of bias in deviations from the intended interventions (performance bias), three had high risk of bias in missing outcome data (attrition bias), and one high risk of bias in selection of reported results (selective outcome reporting bias). NRS – High risk of bias in confounding, high risk of bias in selection of participants into the study (selection bias), unclear risk of bias in deviations from the intended

interventions (performance bias), and high risk of bias in missing outcome data (attrition bias). Overall, six studies had unclear risk of bias and five had high risk of bias.

ae. Wide difference in point estimates, considerable heterogeneity, I^2 >80%.

af. Two RCTs analysed. Two had unclear risk of bias in the randomisation process (selection bias), one high risk of bias in deviations from the intended interventions (performance bias), and one high risk of bias in missing outcome data (attrition bias). Overall, one had unclear risk of bias and one had high risk of bias.

Grade Evidence table: Serum/Plasma zinc, before and after studies (mmol/L)

Certainty assessment							№ of patients		Effect		Certainty	Importance
№ of studies	Study design	Risk of bias	Inconsistency	Indirectness	Imprecision	Other considerations	Before	After	Relative (95% CI)	Absolute (95% CI)		
**Serum/plasma zinc, before and after studies by study design: All studies**												
79	RCTs / NRS	very serious[a]	serious[b]	not serious	serious[c]	none	2829	2931	-	MD **2.85 mmol/L higher** (2.43 higher to 3.28 higher)	⊕◯◯◯ Very low	CRITICAL
**Serum/plasma zinc, before and after studies by sex: Male**												
22	RCTs / NRS	very serious[d]	serious[e]	not serious	serious[c]	none	306	309	-	MD **2.59 mmol/L higher** (1.85 higher to 3.33 higher)	⊕◯◯◯ Very low	IMPORTANT
**Serum/plasma zinc, before and after studies by sex: Female**												
22	RCTs / NRS	very serious[f]	serious[b]	not serious	serious[c]	none	664	665	-	MD **2.82 mmol/L higher** (2.05 higher to 3.6 higher)	⊕◯◯◯ Very low	IMPORTANT
**Serum/plasma zinc, before and after studies by sex: Mixed male and female**												
37	RCTs / NRS	very serious[g]	serious[b]	not serious	serious[c]	none	1859	1957	-	MD **2.96 mmol/L higher** (2.39 higher to 3.54 higher)	⊕◯◯◯ Very low	IMPORTANT

Certainty assessment							№ of patients		Effect		Certainty	Importance
№ of studies	Study design	Risk of bias	Inconsistency	Indirectness	Imprecision	Other considerations	Before	After	Relative (95% CI)	Absolute (95% CI)		
**Serum/plasma zinc, before and after studies by population: Infants (0-12 months)**												
3	RCTs	not serious	serious[h]	not serious	not serious	none	157	174	-	MD **2.8 mmol/L higher** (0.83 higher to 4.78 higher)	⊕⊕⊕◯ Moderate	IMPORTANT
**Serum/plasma zinc, before and after studies by population: Children and adolescents**												
14	RCTs / NRS	very serious[i]	serious[b]	not serious	serious[c]	publication bias strongly suspected[j]	1127	1201	-	MD **2.24 mmol/L higher** (1.38 higher to 3.09 higher)	⊕◯◯◯ Very low	IMPORTANT
**Serum/plasma zinc, before and after studies by population: Pregnancy and lactation**												
3	RCTs	serious[k]	not serious	not serious	not serious	none	155	155	-	MD **0.82 mmol/L higher** (0.86 lower to 2.51 higher)	⊕⊕⊕◯ Moderate	IMPORTANT
**Serum/plasma zinc, before and after studies by population: Adults**												
46	RCTs / NRS	serious[l]	serious[m]	not serious	serious[c]	none	865	872	-	MD **3.28 mmol/L higher** (2.62 higher to 3.94 higher)	⊕◯◯◯ Very low	IMPORTANT
**Serum/plasma zinc, before and after studies by population: Post-menopausal women**												
1	RCTs	not serious	not serious	not serious	not serious	none	57	58	-	MD **5.12 mmol/L higher** (4.42 higher to 5.82 higher)	⊕⊕⊕⊕ High	IMPORTANT

Certainty assessment							№ of patients		Effect		Certainty	Importance
№ of studies	Study design	Risk of bias	Inconsistency	Indirectness	Imprecision	Other considerations	Before	After	Relative (95% CI)	Absolute (95% CI)		
**Serum/plasma zinc, before and after studies by population: Elderly**												
8	RCTs / NRS	very serious[n]	serious[o]	not serious	serious[c]	none	184	187	-	MD **3.23 mmol/L higher** (2.31 higher to 4.16 higher)	⨁◯◯◯ Very low	IMPORTANT
**Serum/plasma zinc, before and after studies by status at baseline: Normal serum zinc level**												
74	RCTs / NRS	very serious[p]	serious[b]	not serious	serious[c]	none	2582	2681	-	MD **2.87 mmol/L higher** (2.43 higher to 3.31 higher)	⨁◯◯◯ Very low	IMPORTANT
**Serum/plasma zinc, before and after studies by status at baseline: Low serum zinc level**												
4	RCTs	serious[q]	serious[r]	not serious	serious[c]	none	247	250	-	MD **2.57 mmol/L higher** (0.89 higher to 4.26 higher)	⨁◯◯◯ Very low	IMPORTANT
**Serum/plasma zinc, before and after studies by dose: Depletion < 3 mg/d Zn**												
2	NRS	very serious[s]	very serious[t]	not serious	serious[c]	none	10	10	-	MD **3.85 mmol/L higher** (5.65 higher to 13.36 higher)	⨁◯◯◯ Very low	IMPORTANT
**Serum/plasma zinc, before and after studies by dose: Depletion 3 to 15 mg/d Zn**												
9	RCTs / NRS	very serious[u]	serious[v]	not serious	serious[c]	publication bias strongly suspected[j]	78	78	-	MD **1.42 mmol/L higher** (0.27 higher to 2.58 higher)	⨁◯◯◯ Very low	IMPORTANT

Certainty assessment							№ of patients		Effect		Certainty	Importance
№ of studies	Study design	Risk of bias	Inconsistency	Indirectness	Imprecision	Other considerations	Before	After	Relative (95% CI)	Absolute (95% CI)		
**Serum/plasma zinc, before and after studies by dose: Supplementation 1 to 2.9 mg/d Zn**												
2	RCTs	very serious[w]	not serious	not serious	not serious	none	87	87	-	MD **1.05 mmol/L higher** (0.3 higher to 1.79 higher)	⊕⊕◯◯ Low	IMPORTANT
**Serum/plasma zinc, before and after studies by dose: Supplementation 3 to 15 mg/d Zn**												
18	RCTs/ NRS	serious[x]	serious[b]	not serious	serious[c]	publication bias strongly suspected[j]	1331	1422	-	MD **2.09 mmol/L higher** (1.46 higher to 2.73 higher)	⊕◯◯◯ Very low	IMPORTANT
**Serum/plasma zinc, before and after studies by dose: Supplementation 16 to 25 mg/d Zn**												
13	RCTs/ NRS	serious[y]	serious[b]	not serious	serious[c]	publication bias strongly suspected[j]	411	412	-	MD **1.74 mmol/L higher** (0.92 higher to 2.57 higher)	⊕◯◯◯ Very low	IMPORTANT
**Serum/plasma zinc, before and after studies by dose: Supplementation 26 to 50 mg/d Zn**												
27	RCTs / NRS	very serious[z]	serious[m]	not serious	serious[c]	none	662	665	-	MD **3.23 mmol/L higher** (2.43 higher to 4.02 higher)	⊕◯◯◯ Very low	IMPORTANT
**Serum/plasma zinc, before and after studies by dose: Supplementation 51 to 100 mg/d Zn**												
8	RCTs / NRS	very serious[aa]	serious[b]	not serious	serious[e]	publication bias strongly suspected[j]	84	84	-	MD **5.19 mmol/L higher** (1.81 higher to 8.58 higher)	⊕◯◯◯ Very low	IMPORTANT

Certainty assessment							№ of patients		Effect		Certainty	Importance
№ of studies	Study design	Risk of bias	Inconsistency	Indirectness	Imprecision	Other considerations	Before	After	Relative (95% CI)	Absolute (95% CI)		
**Serum/plasma zinc, before and after studies by dose: Supplementation 101 to 151 mg/d Zn**												
7	RCTs / NRS	extremely serious[ab]	serious[e]	not serious	serious[c]	publication bias strongly suspected[j]	166	173	-	MD **5.46 mmol/L higher** (2.04 higher to 8.89 higher)	⊕◯◯◯ Very low	IMPORTANT
**Serum/plasma zinc, before and after studies by supplement type: Zinc Sulphate**												
39	RCTs / NRS	very serious[ac]	serious[b]	not serious	serious[c]	none	1919	2018	-	MD **3.22 mmol/L higher** (2.59 higher to 3.85 higher)	⊕◯◯◯ Very low	IMPORTANT
**Serum/plasma zinc, before and after studies by supplement type: Zinc gluconate**												
24	RCTs / NRS	very serious[ad]	serious[e]	not serious	serious[c]	publication bias strongly suspected[j]	706	709	-	MD **2.56 mmol/L higher** (1.94 higher to 3.18 higher)	⊕◯◯◯ Very low	IMPORTANT
**Serum/plasma zinc, before and after studies by supplement type: Zinc acetate**												
3	RCTs / NRS	very serious[ae]	not serious	not serious	not serious	none	94	94	-	MD **3.6 mmol/L higher** (2.87 higher to 4.33 higher)	⊕⊕◯◯ Low	IMPORTANT
**Serum/plasma zinc, before and after studies by supplement type: Depletion**												
11	RCTs / NRS	extremely serious[af]	serious[b]	not serious	serious[e]	publication bias strongly suspected[j]	88	88	-	MD **1.88 mmol/L higher** (0.39 higher to 3.37 higher)	⊕◯◯◯ Very low	IMPORTANT

Certainty assessment							№ of patients		Effect		Certainty	Importance
№ of studies	Study design	Risk of bias	Inconsistency	Indirectness	Imprecision	Other considerations	Before	After	Relative (95% CI)	Absolute (95% CI)		
**Serum/plasma zinc, before and after studies by supplement type: Mixed zinc gluconate and zinc acetate**												
1	NRS	very serious[ag]	not serious	not serious	not serious	none	22	22	-	MD **2.53 mmol/L higher** (0.45 higher to 4.62 higher)	⨁⨁◯◯ Low	IMPORTANT

CI: confidence interval; **MD:** mean difference**; RCT**: randomized control trial; **NRS**: non-randomized studies

Explanations

a. 49 RCTs and 28 NRS and 2 studies not available for RoB assessment. RCTs-High risk of bias in the randomization process (selection bias), deviations from intended interventions (performance bias), missing outcome data (attrition bias), measurement of the outcome (detection bias), selection of the reported results (selective outcome reporting bias). NRS–high risk of bias in confounding, selection of participants into the study, classification of the interventions (selection bias), deviations from intended interventions (performance bias), missing outcome data (attrition bias), measurement of the outcome (detection bias), selection of the reported results (selective outcome reporting bias). Overall, 40/79 had high risk of bias.

b. Wide difference in point estimates, considerable heterogeneity, I^2 >95%.

c. Small number of events, wide confidence intervals including appreciable benefit and harm.

d. 22 studies included in the analysis, 11 RCTs, 10 NRS, 1 study was not available for RoB. RCTs – 5 studies had unclear risk of bias and 1 had high risk of bias in randomization process (selection bias), 2 had high risk of bias in deviations from intended interventions (performance bias), and 2 had high risk of bias in missing outcome data (attrition bias). NRS - 6 had high risk of bias in confounding, 10 had high risk of bias in selection of participants into the study (selection bias), 2 had high risk of bias in deviations from intended interventions (performance bias), and 3 had high risk of bias in missing outcome data (attrition bias). Overall, 14 had high risk of bias.

e. Wide difference in point estimates, considerable heterogeneity, I^2 >90%.

f. 22 studies included in the analysis, 15 RCTs and 7 NRS. RCTs – 8 had unclear risk of bias in randomization process (selection bias), 2 had high risk of bias in deviations from intended interventions (performance bias), 3 had high risk of bias in missing outcome data (attrition bias), and 1 had high risk of bias in measurement of the outcome (detection bias). NRS - 6 had high risk of bias in confounding, 7 had high risk of bias in selection of participants into the study (selection bias), and 1 had high risk of bias in in deviations from intended interventions (performance bias). Overall, 10 had high risk of bias and 6 had unclear risk of bias.

g. 37 studies analysed, 24 RCTs, 12 NRS, 1 study not available for RoB. RCTs- 2 had unclear risk of bias in randomization process (selection bias), 1 had high risk of bias in deviations from intended interventions (performance bias), 5 had had high risk of bias in missing outcome data (attrition bias), and 1 had high risk of bias in the selection of the reported results (selective outcome reporting bias). NRS–7 had high risk of bias in confounding, 12 had high risk of bias in selection of participants into the study and 2 had high risk of bias in classification of the interventions (selection bias), 1 had high risk of bias in deviations from intended interventions (performance bias), 2 had high risk of bias in missing outcome data (attrition bias), 2 had high risk of bias in measurement of the outcome (detection bias), and 2 had high risk of bias in the selection of the reported results (selective outcome reporting bias). Overall, 18 had high risk of bias and 7 had unclear risk of bias.

h. Considerable heterogeneity, I^2 >95%.

i. 14 papers were in included in the analysis, 11 RCTs and 3 NRS. RCTs – 2 had high risk of bias in missing outcome data (attrition bias), and 1 had high risk of bias in measurement of the outcome (detection bias). NRS – 2 had high risk of bias in confounding and 3 had high risk of bias in selection of participants into the study (selection bias), 1 had high risk of bias in missing outcome data (attrition bias), and 1 had high risk of bias in measurement of the outcome (detection bias). Overall, 5 had high risk of bias and 3 had unclear risk of bias.

j. Publication bias suspected because of asymmetrical funnel plot.

k. 3 RCTs were included in the analysis. 2 had unclear risk of bias in randomization process (selection bias). Overall, 2 had unclear risk of bias.

l. 46 papers were included in the analysis, 25 RCTs, 18 NRS, 2 papers were unavailable for RoB. RCTs – 12 had unclear risk of bias and 1 had high risk of bias in randomization process (selection bias). 3 had high risk of bias in deviations from intended interventions (performance bias), and 5 had high risk of bias in missing outcome data (attrition bias). NRS - 10 had high risk of bias and 9 had unclear risk of bias in confounding, 19 had high risk of bias in selection of participants into the study (selection bias), 2 had high risk of bias in deviations from intended interventions (performance bias), 2 had high risk of bias in missing outcome data (attrition bias), and 1 had high risk of bias in the selection of the reported results (selective outcome reporting bias). Overall, 26 had high risk of bias and 8 had unclear risk of bias.

m. Wide difference in point estimates, considerable heterogeneity, I^2 >90%.

n. 8 papers were included in the analysis, 5 RCTs and 3 NRS. RCTs – 5 had unclear risk of bias in the randomization process (selection bias), 1 had high risk of bias in deviations from intended interventions (performance bias), and 3 had high risk of bias in missing outcome data (attrition bias). NRS – 3 had high risk of bias in confounding, 3 had high risk of bias in selection of participants into the study, and 1 had high risk of bias in

classification of the interventions (selection bias), 1 had high risk of bias in deviations from intended interventions (performance bias), 2 had high risk of bias in missing outcome data (attrition bias), 1 had high risk of bias in measurement of the outcome (detection bias), and 1 had high risk of bias in the selection of the reported results (selective outcome reporting bias). Overall, 6 had high risk of bias.

o. Considerable heterogeneity, I^2 >55%.

p. 74 papers analysed, 45 RCTs, 27 NRS, 2 papers unavailable for RoB. RCTs–High risk of bias in the randomization process (selection bias), high risk of bias in deviations from intended interventions (performance bias), high risk of bias in missing outcome data (attrition bias), high risk of bias in measurement of the outcome (detection bias), high risk of bias in the selection of the reported results (selective outcome reporting bias). NRS-high risk of bias in confounding, selection of participants into the study and classification of the interventions (selection bias), high risk of bias in deviations from intended interventions (performance bias), high risk of bias in missing outcome data (attrition bias), high risk of bias in measurement of the outcome (detection bias), high risk of bias in the selection of the reported results (selective outcome reporting bias). Overall, 40 papers had high risk of bias.

q. 4 RCTs included in the analysis. 2 had unclear risk of bias the randomization process (selection bias). Overall, 2 had unclear risk of bias.

r. Considerable heterogeneity, I^2 >90%.

s. 2 NRS included in the analysis. 2 had high risk of bias in confounding and selection of participants into the study (selection bias), 1 had high risk of bias in deviations from intended interventions (performance bias), and 1 had high risk of bias in missing outcome data (attrition bias). Overall, 2 had high risk of bias.

t. Wide difference in point estimates, confidence intervals do not overlap, considerable heterogeneity, I^2 >95%.

u. 9 papers were included in the analysis, 1 RCT and 7 NRS, 1 paper was unavailable for RoB. RCT – Unclear risk of bias in the randomization process (selection bias). NRS – 4 had unclear risk of bias and 3 had high risk of bias in confounding, 7 had high risk of bias in selection of participants into the study (selection bias), and 1 had high risk of bias in missing outcome data (attrition bias). Overall, 1 had unclear risk of bias and 7 had high risk of bias.

v. Wide difference in point estimates, considerable heterogeneity, I^2 >85%.

w. 2 RCTs were included in the analysis. 1 had unclear risk of bias in the randomization process (selection bias), 1 had unclear risk of bias in deviations from intended interventions (performance bias), and 1 high risk of bias in in missing outcome data (attrition bias). Overall, 1 had high risk of bias.

x. 18 papers were included in the analysis, 15 RCTs and 3 NRS. RCTs – 1 had high risk of bias in missing outcome data (attrition bias), and 1 had high risk of bias in the selection of the reported results (selective outcome reporting bias). NRS – 2 had high risk of bias in confounding, and 3 had high risk of bias in selection of participants into the study (selection bias). Over all 5 had high risk of bias.

y. 13 papers were included in the analysis, 10 RCTs and 3 NRS. RCTs - 1 had high risk of bias in missing outcome data (attrition bias), and 1 had high risk of bias in measurement of the outcome (detection bias). NRS – 3 had high risk of bias in confounding, 3 had high risk of bias in selection of participants into the study, and 1 had high risk of bias in classification of the interventions (selection bias). Overall, 4 had high risk of bias.

z. 27 papers analysed, 20 RCTs and 7 NRS. RCTs – 1 had high risk of bias in the randomization process (selection bias), 3 had high risk of bias in deviations from intended interventions (performance bias), 7 had high risk of bias in missing outcome data (attrition bias), and 1 had high risk of bias in the selection of the reported results (selective outcome reporting bias). NRS - 5 had high risk of bias in confounding, 7 had high risk of bias in selection of participants into the study, and 1 had high risk of bias in classification of the interventions (selection bias), 1 had high risk of bias in deviations from intended interventions (performance bias), 3 had high risk of bias in missing outcome data (attrition bias), 2 had high risk of bias in measurement of the outcome (detection bias), and 1 had high risk of bias in the selection of the reported results (selective outcome reporting bias). Overall, 16 had high risk of bias.

aa. 8 papers were included in the analysis, 5 RCTs and 3 NRS. RCTs – 4 had unclear risk of bias and 1 had high risk of bias in the randomization process (selection bias), and 2 had high risk of bias in missing outcome data (attrition bias). NRS - 3 had high risk of bias in confounding and 3 had high risk of bias in selection of participants into the study (selection bias). Overall, 5 had high risk of bias.

ab. 7 papers were included in the analysis, 3 RCTs and 3 NRS, 1 paper was unavailable for RoB. RCTs – 2 had unclear risk of bias in the randomization process (selection bias), 1 had unclear risk of bias and 1 had high risk of bias in deviations from intended interventions (performance bias), and 2 had high risk of bias in missing outcome data (attrition bias). NRS – 2 had unclear risk of bias and 1 had high risk of bias in confounding, and 3 had high risk of bias in selection of participants into the study (selection bias), 1 had high risk of bias in deviations from intended interventions (performance bias), 2 had unclear risk of bias in missing outcome data (attrition bias), and 1 had high risk of bias in the selection of the reported results (selective outcome reporting bias). Overall, 6 papers had high risk of bias.

ac. 39 papers analysed, 29 RCTs and 9 NRS, 1 paper unavailable for RoB. RCTs–11 had unclear risk of bias in the randomization process (selection bias), 2 had high risk of bias in deviations from intended interventions (performance bias), 5 had high risk of bias in missing outcome data (attrition bias), and 1 had high risk of bias in measurement of the outcome (detection bias). NRS-6 had high risk of bias in confounding, 9 had high risk of bias in selection of participants into the study, and 1 had high risk of bias in classification of the interventions (selection bias), 1 had high risk of bias in deviations from intended interventions (performance bias), 1 had high risk of bias in missing outcome data (attrition bias), 1 had high risk of bias in measurement of the outcome (detection bias), and 1 had high risk of bias in the selection of the reported results (selective outcome reporting bias). Overall, 16 had high risk of bias.

ad. 24 papers were included in the analysis, 17 RCTs and seven NRS. RCTs - 11 had unclear risk of bias and one had high risk of bias in the randomization process (selection bias), one had high risk of bias in deviations from intended interventions (performance bias), four had high risk of bias in missing outcome data (attrition bias), and one had high risk of bias in the selection of the reported results (selective outcome reporting bias). NRS - Two had unclear risk of bias and five had high risk of bias in confounding, seven had high risk of bias in selection of participants into the

study (selection bias), and one had high risk of bias in missing outcome data (attrition bias). Overall, 12 had high risk of bias and six had unclear risk of bias.

ae. 3 papers were included in the analysis, 2 RCTs and 1 NRS. RCTs – 2 had unclear bias in the randomization process (selection bias), 1 had high risk of bias in deviations from intended interventions (performance bias), and 1 had high risk of bias in missing outcome data (attrition bias). NRS – High risk of bias in confounding, selection of participants into the study and classification of the interventions (selection bias), deviations from intended interventions (performance bias), missing outcome data (attrition bias), measurement of the outcome (detection bias), and the selection of the reported results (selective outcome reporting bias). Overall, 2 had high risk of bias and 1 had unclear risk of bias.

af. 11 papers were included in the analysis, 1 RCT and 9 NRS, 1 paper was unavailable for RoB. RCT – Unclear risk of bias in the randomization process (selection bias). NRS – 4 had unclear risk of bias and 5 had high risk of bias in confounding, and 9 had high risk of bias in selection of participants into the study (selection bias), 1 had high risk of bias in deviations from intended interventions (performance bias), and 2 had high risk of bias in missing outcome data (attrition bias). Overall, 9 had high risk of bias.

ag. 1 NRS. High risk of bias in confounding and selection of participants into the study (selection bias).

Grade Evidence table: Urinary zinc

Certainty assessment							№ of patients		Effect		Certainty	Importance
№ of studies	Study design	Risk of bias	Inconsistency	Indirectness	Imprecision	Other considerations	Urinary zinc	Control	Relative (95% CI)	Absolute (95% CI)		
**Urinary Zinc (mmol/mol Creatinine): All studies**												
4	RCTs / NRS	very serious[a]	serious[b]	not serious	serious[c]	none	311	176	-	MD **0.39 mmol/mol Creatinine higher** (0.17 higher to 0.62 higher)	⨁◯◯◯ Very low	CRITICAL
**Urinary Zinc (mmol/mol Creatinine) by sex: Males**												
2	RCTs	serious[d]	not serious	not serious	serious[c]	none	43	35	-	MD **0.71 mmol/mol Creatinine higher** (0.53 higher to 0.89 higher)	⨁⨁◯◯ Low	IMPORTANT
**Urinary Zinc (mmol/mol Creatinine) by sex: Females**												
1	NRS	very serious[e]	not serious	not serious	serious[c]	none	11	11	-	MD **0.27 mmol/mol Creatinine higher** (0.02 higher to 0.52 higher)	⨁◯◯◯ Very low	IMPORTANT
**Urinary Zinc (mmol/mol Creatinine) by sex: Mixed males and females**												
1	RCT	very serious[f]	not serious	not serious	serious[c]	none	257	130	-	MD **0.21 mmol/mol Creatinine higher** (0.03 higher to 0.4 higher)	⨁◯◯◯ Very low	IMPORTANT
**Urinary Zinc (mmol/mol Creatinine) by population: Children and adolescents**												
1	RCT	not serious	serious[g]	not serious	not serious	none	21	26	-	MD **0.77 mmol/mol Creatinine higher** (0.56 higher to 0.98 higher)	⨁⨁⨁◯ Moderate	IMPORTANT

Certainty assessment							№ of patients		Effect		Certainty	Importance
№ of studies	Study design	Risk of bias	Inconsistency	Indirectness	Imprecision	Other considerations	Urinary zinc	Control	Relative (95% CI)	Absolute (95% CI)		
**Urinary Zinc (mmol/mol Creatinine) by population: Adults**												
3	RCTs / NRS	very serious[h]	serious[i]	not serious	serious[c]	none	290	150	-	MD **0.25 mmol/mol Creatinine higher** (0.13 higher to 0.37 higher)	⊕◯◯◯ Very low	IMPORTANT
**Urinary Zinc (mmol/mol Creatinine) by dose: Supplementation 15 to 25 mg Zn/d**												
3	RCTs / NRS	very serious[j]	serious[k]	not serious	serious[c]	none	158	102	-	MD **0.38 mmol/mol Creatinine higher** (0.03 lower to 0.79 higher)	⊕◯◯◯ Very low	IMPORTANT
**Urinary Zinc (mmol/mol Creatinine) by dose: Supplementation 26 to 50 mg Zn/d**												
2	RCTs	very serious[l]	not serious	not serious	serious[c]	none	144	70	-	MD **0.32 mmol/mol Creatinine higher** (0.18 higher to 0.47 higher)	⊕◯◯◯ Very low	IMPORTANT
**Urinary Zinc (mmol/mol Creatinine) by dose: Supplementation 51 to 100 mg Zn/d**												
1	RCT	very serious[m]	not serious	not serious	serious[c]	none	9	4	-	MD **0.59 mmol/mol Creatinine higher** (0.04 lower to 1.22 higher)	⊕◯◯◯ Very low	IMPORTANT
**Urinary Zinc (mmol/mol Creatinine) by supplement type: Zinc gluconate**												
4	RCTs / NRS	very serious[a]	serious[b]	not serious	serious[c]	none	311	176	-	MD **0.39 mmol/mol Creatinine higher** (0.17 higher to 0.62 higher)	⊕◯◯◯ Very low	IMPORTANT

Certainty assessment							№ of patients		Effect		Certainty	Importance
№ of studies	Study design	Risk of bias	Inconsistency	Indirectness	Imprecision	Other considerations	Urinary zinc	Control	Relative (95% CI)	Absolute (95% CI)		
**Urinary Zinc (µmol/d): All studies**												
6	RCTs / NRS	very serious[n]	serious[k]	not serious	serious[c]	publication bias strongly suspected[o]	71	64	-	MD **3.09 µmol/d higher** (0.16 higher to 6.02 higher)	⊕◯◯◯ Very low	IMPORTANT
**Urinary Zinc (µmol/d) by sex: Males**												
4	RCTs / NRS	very serious[p]	serious[k]	not serious	serious[c]	none	36	33	-	MD **3.87 µmol/d higher** (0.25 higher to 7.49 higher)	⊕◯◯◯ Very low	IMPORTANT
**Urinary Zinc (µmol/d) by sex: Females**												
3	RCTs / NRS	very serious[q]	serious[r]	not serious	serious[c]	none	35	31	-	MD **2.99 µmol/d higher** (0.7 lower to 6.67 higher)	⊕◯◯◯ Very low	IMPORTANT
**Urinary Zinc (µmol/d) by population: Adults**												
4	RCTs / NRS	very serious[s]	serious[k]	not serious	serious[c]	none	49	49	-	MD **2.5 µmol/d higher** (1.01 lower to 6 higher)	⊕◯◯◯ Very low	IMPORTANT
**Urinary Zinc (µmol/d) by population: Elderly**												
1	RCT	very serious[t]	not serious	not serious	not serious	none	17	10	-	MD **9.3 µmol/d higher** (5.98 higher to 12.62 higher)	⊕⊕◯◯ Low	IMPORTANT

Certainty assessment							№ of patients		Effect		Certainty	Importance
№ of studies	Study design	Risk of bias	Inconsistency	Indirectness	Imprecision	Other considerations	Urinary zinc	Control	Relative (95% CI)	Absolute (95% CI)		
**Urinary Zinc (µmol/d) by dose: Depletion <5 mg Zn/d**												
4	RCTs / NRS	very serious[u]	serious[k]	not serious	serious[c]	none	29	29	-	MD **2.98 µmol/d higher** (0.48 lower to 6.43 higher)	⊕◯◯◯ Very low	IMPORTANT
**Urinary Zinc (µmol/d) by dose: Supplementation 15 to 25 mg Zn/d**												
1	RCT	serious[v]	not serious	not serious	not serious	none	5	5	-	MD **0.3 µmol/d lower** (2.11 lower to 1.51 higher)	⊕⊕⊕◯ Moderate	IMPORTANT
**Urinary Zinc (µmol/d) by dose: Supplementation 26 to 50 mg Zn/d**												
2	RCTs	very serious[w]	very serious[x]	not serious	serious[c]	none	37	30	-	MD **5.31 µmol/d higher** (2.41 lower to 13.03 higher)	⊕◯◯◯ Very low	IMPORTANT
**Urinary Zinc (µmol/d) by supplement type: Zn sulphate**												
1	RCT	serious[v]	not serious	not serious	not serious	none	5	5	-	MD **0.3 µmol/d lower** (2.11 lower to 1.51 higher)	⊕⊕⊕◯ Moderate	IMPORTANT
**Urinary Zinc (µmol/d) by supplement type: Zn gluconate**												
1	RCT	very serious[t]	not serious	not serious	serious[y]	none	20	20	-	MD **1.42 µmol/d higher** (1.44 lower to 4.28 higher)	⊕◯◯◯ Very low	IMPORTANT

Certainty assessment							№ of patients		Effect		Certainty	Importance
№ of studies	Study design	Risk of bias	Inconsistency	Indirectness	Imprecision	Other considerations	Urinary zinc	Control	Relative (95% CI)	Absolute (95% CI)		
**Urinary Zinc (µmol/d) by supplement type: Zn acetate**												
1	RCT	serious[t]	not serious	not serious	serious[z]	none	17	10	-	MD **9.3 µmol/d higher** (5.98 higher to 12.62 higher)	⨁⨁◯◯ Low	IMPORTANT
**Urinary Zinc (µmol/L): All studies**												
4	RCTs / NRS	serious[aa]	serious[k]	not serious	serious[c]	none	63	64	-	MD **2.88 µmol/L higher** (1.55 lower to 7.31 higher)	⨁◯◯◯ Very low	IMPORTANT
**Urinary Zinc (µmol/L) by sex: Males**												
1	NRS	very serious[ab]	not serious	not serious	serious[y]	none	14	15	-	MD **1.6 µmol/L lower** (9.29 lower to 6.09 higher)	⨁◯◯◯ Very low	IMPORTANT
**Urinary Zinc (µmol/L) by sex: Females**												
2	RCTs / NRS	serious[ac]	very serious[x]	not serious	serious[ad]	none	34	34	-	MD **4.38 µmol/L higher** (2.49 lower to 11.25 higher)	⨁◯◯◯ Very low	IMPORTANT
**Urinary Zinc (µmol/L) by sex: Mixed**												
1	RCT	not serious	not serious	not serious	serious[y]	none	15	15	-	MD **2.29 µmol/L higher** (0.35 higher to 4.23 higher)	⨁⨁⨁◯ Moderate	IMPORTANT

Certainty assessment							№ of patients		Effect		Certainty	Importance
№ of studies	Study design	Risk of bias	Inconsistency	Indirectness	Imprecision	Other considerations	Urinary zinc	Control	Relative (95% CI)	Absolute (95% CI)		
**Urinary Zinc (µmol/L) by population: Children and adolescents**												
1	NRS	very serious[e]	not serious	not serious	not serious	none	10	10	-	MD **7.87 µmol/L higher** (6.79 higher to 8.96 higher)	⊕⊕◯◯ Low	IMPORTANT
**Urinary Zinc (µmol/L) by population: Adults**												
3	RCTs / NRS	serious[ae]	not serious	not serious	serious[c]	none	53	54	-	MD **1.28 µmol/L higher** (0.16 higher to 2.39 higher)	⊕⊕◯◯ Low	IMPORTANT
**Urinary Zinc (µmol/L) by dose: Depletion <5 mg Zn/d**												
1	NRS	very serious[ab]	not serious	not serious	serious[y]	none	14	15	-	MD **1.6 µmol/L higher** (9.29 lower to 6.09 higher)	⊕◯◯◯ Very low	IMPORTANT
**Urinary Zinc (µmol/L) by dose: Supplementation 15 to 25 mg Zn/d**												
1	RCT	not serious	not serious	not serious	not serious	none	24	24	-	MD **0.86 µmol/L higher** (0.52 lower to 2.24 higher)	⊕⊕⊕⊕ High	IMPORTANT
**Urinary Zinc (µmol/L) by dose: Supplementation 26 to 50 mg Zn/d**												
2	RCTs / NRS	serious[ac]	serious[k]	not serious	serious[ad]	none	25	25	-	MD **5.14 µmol/L higher** (0.33 lower to 10.61 higher)	⊕◯◯◯ Very low	IMPORTANT

Certainty assessment							№ of patients		Effect		Certainty	Importance
№ of studies	Study design	Risk of bias	Inconsistency	Indirectness	Imprecision	Other considerations	Urinary zinc	Control	Relative (95% CI)	Absolute (95% CI)		
**Urinary Zinc (µmol/L) by supplement type: Zinc sulphate**												
2	RCTs / NRS	serious[ac]	very serious[x]	not serious	serious[ad]	none	34	34	-	MD **4.38 µmol/L higher** (2.49 lower to 11.25 higher)	⊕◯◯◯ Very low	IMPORTANT
**Urinary Zinc (µmol/L) by supplement type: Zinc gluconate**												
1	RCT	not serious	not serious	not serious	serious[y]	none	15	15	-	MD **2.29 µmol/L higher** (0.35 higher to 4.23 higher)	⊕⊕⊕◯ Moderate	IMPORTANT

CI: confidence interval; **MD:** mean difference; **RCT**: randomized control trial; **NRS**: non-randomized studies

Explanations

a. Four studies were included in the analysis, three RCTs and 1 NRS. RCTs - One had high risk of bias and one had unclear risk of bias in the randomization process (selection bias), one had high risk of bias in missing outcome data (attrition bias), and one had high risk of bias in the selection of the reported results (selective outcome reporting bias). NRS – High risk of bias in confounding, and selection of participants into the study (selection bias). Overall, three had high risk of bias.

b. Wide difference in point estimates, considerable heterogeneity, $I^2 > 80\%$.

c. Small number of events, wide confidence intervals including appreciable benefit and harm.

d. Two RCTs were included in the analysis. One had high risk of bias in the randomization process (selection bias), and one had high risk of bias in missing outcome data (attrition bias). Overall, one had high risk of bias.

e. One NRS included in the analysis – High risk of bias in confounding, and high risk of bias in selection of participants into the study (selection bias).

f. One RCT paper only. Unclear risk of bias in the randomization process (selection bias), high risk of bias in the selection of the reported results (selective outcome reporting bias).

g. $I^2 > 100\%$.

h. Three studies included in the analysis, two RCTs and one NRS. RCTs- One had high risk of bias and one had unclear risk of bias in the randomization process (selection bias), one had high risk of bias in missing outcome data (attrition bias), and one had high risk of bias in the selection of the reported results (selective outcome reporting bias). NRS – High risk of bias in confounding, and selection of participants into the study (selection bias). Overall, three had high risk of bias.

i. Wide difference in point estimates.

j. Three studies included in the analysis, two RCTs and one NRS. RCTs- One had unclear risk of bias in the randomization process (selection bias), and one had high risk of bias in the selection of the reported results (selective outcome reporting bias). NRS - High risk of bias in confounding and selection of participants into the study (selection bias). Overall, two had high risk of bias.

k. Wide difference in point estimates, considerable heterogeneity, $I^2 > 90\%$.

l. Two RCTs were included in the analysis. One had high risk of bias and one had unclear risk of bias in the randomization process (selection bias), one had high risk of bias in missing outcome data (attrition bias), and one had high risk of bias in the selection of the reported results (selective outcome reporting bias). Overall, two had high risk of bias.

m. One RCTs included in the analysis. High risk of bias in the randomization process (selection bias), and high risk of bias in missing outcome data (attrition bias).

n. Six studies were included in the analysis, three RCTs and three NRS. RCTs- Three had unclear risk of bias in the randomization process (selection bias), two had high risk of bias in deviations from intended interventions (performance bias), and two had high risk of bias in missing outcome data (attrition bias). NRS – One had unclear risk of bias and two had high risk of bias in confounding, and three had high risk of bias in selection of participants into the study (selection bias), one had high risk of bias in deviations from intended interventions (performance bias), and one had high risk of bias in missing outcome data (attrition bias). Overall, five had high risk of bias and one had unclear risk of bias.

o. Publication bias suspected because of asymmetrical funnel plot.

p. Four studies were included in the analysis, two RCTs and two NRS. RCTs - Two had unclear risk of bias in the randomization process (selection bias), one had high risk of bias in deviations from intended interventions (performance bias), and one had high risk of bias in missing outcome data (attrition bias). NRS – One had unclear risk of bias and one had high risk of bias in confounding, and two had high risk of bias in selection of participants into the study (selection bias), one had high risk of bias in deviations from intended interventions (performance bias), and one had high risk of bias in missing outcome data (attrition bias). Overall, all three had high risk of bias and one had unclear risk of bias.

q. Three studies were included in the analysis, two RCTs and one NRS. RCTs. Two had unclear risk of bias in the randomization process (selection bias), two had high risk of bias in deviations from intended interventions (performance bias), and two had high risk of bias in missing outcome data (attrition bias). NRS – High risk of bias in confounding and selection of participants into the study (selection bias). Overall, all three had high risk of bias.

r. Wide difference in point estimates, considerable heterogeneity, $I^2 > 75\%$.

s. Four studies were included in the analysis, two RCTs and two NRS. RCTs- Two had unclear risk of bias in the randomization process (selection bias), one had high risk of bias in deviations from intended interventions (performance bias), and one had high risk of bias in missing outcome data (attrition bias). NRS – One had unclear risk of bias and one had high risk of bias in confounding, and two had high risk of bias in selection of participants into the study (selection bias), one had high risk of bias in deviations from intended interventions (performance bias), and one had high risk of bias in missing outcome data (attrition bias). Overall, three had high risk of bias and one had unclear risk of bias.

t. One RCT included in the analysis. Unclear risk of bias in the randomization process (selection bias), high risk of bias in deviations from intended interventions (performance bias), and high risk of bias in missing outcome data (attrition bias).

u. Four studies were included in the analysis, one RCTs and three NRS. RCTs - One had unclear risk of bias in the randomization process (selection bias). NRS – One had unclear risk of bias and two had high risk of bias in confounding, and three had high risk of bias in selection of participants into the study (selection bias), one had high risk of bias in deviations from intended interventions (performance bias), and one had high risk of bias in missing outcome data (attrition bias). Overall, three had high risk of bias and one had unclear risk of bias.

v. One RCT included in the analysis. Unclear risk of bias in the randomization process (selection bias).

w. Two RCTs were included in the analysis. Two had unclear risk of bias in the randomization process (selection bias), two had high risk of bias in deviations from intended interventions (performance bias), and two had high risk of bias in missing outcome data (attrition bias). Overall, two had high risk of bias.

x. Wide difference in point estimates, confidence intervals do not overlap, considerable heterogeneity, $I^2 >90\%$.

y. Wide confidence intervals including appreciable benefit and harm.

z. Wide confidence intervals.

aa. Four studies were included in the analysis, two RCTs and two NRS. RCTs - Both at low risk. NRS – One had unclear risk of bias and one had high risk of bias in confounding, and two had high risk of bias in selection of participants into the study (selection bias). Overall, two had high risk of bias and one had unclear risk of bias.

ab. One NRS included in the analysis. Unclear risk of bias in confounding, and high risk of bias in selection of participants into the study (selection bias).

ac. Two studies were included in the analysis, one RCT and one NRS. RCT at low risk of bias. NRS – High risk of bias in confounding and selection of participants into the study (selection bias). Overall, one had high risk of bias

ad. Small number of events, wide estimate points indicate appreciable benefit and harm..

ae. Three studies were included in the analysis, two RCTs and one NRS. RCTs - Both at low risk. NRS –Unclear risk of bias in confounding and high risk of bias in selection of participants into the study (selection bias). Overall, one had high risk of bias.

Grade Evidence table: Alkaline phosphatase (ALP; U/L)

Certainty assessment							№ of patients		Effect		Certainty	Importance
№ of studies	Study design	Risk of bias	Inconsistency	Indirectness	Imprecision	Other considerations	ALP	Control	Relative (95% CI)	Absolute (95% CI)		
**Alkaline phosphatase (U/L): All studies**												
7	RCTs / NRS	very serious[a]	serious[b]	not serious	serious[c]	publication bias strongly suspected[d]	364	237	-	MD **3.88 higher** (0.43 higher to 7.33 higher)	⊕◯◯◯ Very low	CRITICAL
**Alkaline phosphatase (U/L) by sex: Males**												
1	NRS	very serious[e]	not serious	not serious	not serious	none	5	5	-	MD **21.8 higher** (8.91 higher to 34.69 higher)	⊕⊕◯◯ Low	IMPORTANT
**Alkaline phosphatase (U/L) by sex: Female**												
3	RCTs / NRS	very serious[f]	not serious	not serious	serious[c]	none	55	55	-	MD **5.44 higher** (1.38 lower to 12.25 higher)	⊕◯◯◯ Very low	IMPORTANT
**Alkaline phosphatase (U/L) by sex: Mixed male and female**												
3	RCTs / NRS	very serious[g]	not serious	not serious	serious[c]	none	304	177	-	MD **1.72 higher** (0.14 higher to 3.3 higher)	⊕◯◯◯ Very low	IMPORTANT
**Alkaline phosphatase (U/L) by intake: Depletion < 3 mg/d Zn**												
2	NRS	very serious[h]	serious[i]	not serious	serious[c]	none	10	10	-	MD **12.17 higher** (6.47 lower to 31.09 higher)	⊕◯◯◯ Very low	IMPORTANT

Certainty assessment							№ of patients		Effect		Certainty	Importance
№ of studies	Study design	Risk of bias	Inconsistency	Indirectness	Imprecision	Other considerations	ALP	Control	Relative (95% CI)	Absolute (95% CI)		
**Alkaline phosphatase (U/L) by intake: Supplementation 3 to 15 mg/d Zn**												
2	RCTs / NRS	very serious[j]	not serious	not serious	serious[c]	none	141	80	-	MD **1.78 higher** (0.13 higher to 3.44 higher)	⊕◯◯◯ Very low	IMPORTANT
**Alkaline phosphatase (U/L) by intake: Supplementation 16 to 25 mg/d Zn**												
1	RCT	serious[k]	not serious	not serious	serious[l]	none	30	30	-	MD **12 higher** (11.81 lower to 35.81 higher)	⊕⊕◯◯ Low	IMPORTANT
**Alkaline phosphatase (U/L) by intake: Supplementation 26 to 50 mg/d Zn**												
2	RCT	very serious[m]	not serious	not serious	serious[l]	none	151	85	-	MD **2.33 higher** (2.23 lower to 6.89 higher)	⊕◯◯◯ Very low	IMPORTANT
**Alkaline phosphatase (U/L) by intake: Supplementation 51 to 100 mg/d Zn**												
1	RCT	serious[n]	not serious	not serious	serious[l]	none	32	32	-	MD **6 higher** (21.65 lower to 33.65 higher)	⊕⊕◯◯ Low	IMPORTANT
**Alkaline phosphatase (U/L) by supplementation type: Zinc sulphate**												
1	RCT	serious[k]	not serious	not serious	serious[l]	none	30	30	-	MD **12 higher** (11.81 lower to 35.81 higher)	⊕⊕◯◯ Low	IMPORTANT
**Alkaline phosphatase (U/L) by supplementation type: Zinc gluconate**												
2	RCTs	very serious[m]	not serious	not serious	serious[o]	none	277	150	-	MD **2.76 higher** (1.11 lower to 6.64 higher)	⊕◯◯◯ Very low	IMPORTANT

Certainty assessment							№ of patients		Effect		Certainty	Importance
**№ of studies**	**Study design**	**Risk of bias**	**Inconsistency**	**Indirectness**	**Imprecision**	**Other considerations**	**ALP**	**Control**	**Relative (95% CI)**	**Absolute (95% CI)**		
**Alkaline phosphatase (U/L) by supplementation type: Zinc acetate**												
1	RCT	serious[n]	not serious	not serious	serious[l]	none	32	32	-	MD **6 higher** (21.65 lower to 33.65 higher)	⊕⊕◯◯ Low	IMPORTANT
**Alkaline phosphatase (U/L) by supplementation type: Depletion**												
3	NRS	very serious[p]	serious[i]	not serious	serious[c]	none	25	25	-	MD **7.63 higher** (4.02 lower to 19.28 higher)	⊕◯◯◯ Very low	IMPORTANT

CI: confidence interval; **MD:** mean difference**; RCT**: randomized control trial; **NRS**: non-randomized studies

Explanations

a. Seven papers included in the analysis, four RCTs and three NRS. RCTs – Four had unclear risk of bias in the randomization process (selection bias), one had high risk of bias in deviations from intended interventions (performance bias), one had high risk of bias in missing outcome data (attrition bias), one had unclear risk of bias and one had high risk of bias in the selection of the reported results (selective outcome reporting bias). NRS – Three had high risk of bias in confounding and selection of participants into the study (selection bias), one had high risk of bias in deviations from intended interventions (performance bias), and one had high risk of bias in missing outcome data (attrition bias). Overall, five had high risk of bias and two had unclear risk of bias.

b. Wide difference in point estimates, considerable heterogeneity, I^2 >35%.

c. Small number of events, wide confidence intervals including appreciable benefit and harm.

d. Publication bias suspected because of asymmetrical funnel plot.

e. One NRS included in the analysis - High risk of bias in confounding and selection of participants into the study (selection bias), high risk of bias in deviations from intended interventions (performance bias), and high risk of bias in missing outcome data (attrition bias).

f. Three papers included in the analysis, two RCTs and one NRS. RCTs – Two had unclear risk of bias in the randomization process (selection bias), one had high risk of bias in deviations from intended interventions (performance bias), one had high risk of bias in missing outcome data (attrition bias), and one unclear risk of bias in the selection of the reported results (selective outcome reporting bias). NRS –High risk of bias in confounding and selection of participants into the study (selection bias). Overall, two had high risk of bias and one had unclear risk of bias.

g. Three papers included in the analysis, two RCTs and one NRS. RCTs – Two had unclear risk of bias in the randomization process (selection bias), and one had high risk of bias in the selection of the reported results (selective outcome reporting bias). NRS – High risk of bias in confounding and selection of participants into the study (selection bias). Overall, two had high risk of bias and one had unclear risk of bias.

h. Two NRS included in the analysis. NRS – Two had high risk of bias in confounding and selection of participants into the study (selection bias), one had high risk of bias in deviations from intended interventions (performance bias), and one had high risk of bias in missing outcome data (attrition bias). Overall, two had high risk of bias.

i. Wide difference in point estimates, serious heterogeneity, I^2 >75%.

j. Two papers included in the analysis, one RCTs and one NRS. RCTs – Unclear risk of bias in the randomization process (selection bias), and high risk of bias in the selection of the reported results (selective outcome reporting bias). NRS – High risk of bias in confounding and selection of participants into the study (selection bias). Overall, two had high risk of bias.

k. One RCT - Unclear risk of bias in the randomization process (selection bias), and unclear risk of bias in the selection of the reported results (selective outcome reporting bias).

l. Wide confidence intervals including appreciable benefit and harm.

m. Two RCTs included in the analysis - Two had unclear risk of bias in the randomization process (selection bias), one had high risk of bias in deviations from intended interventions (performance bias), one had high risk of bias in missing outcome data (attrition bias), and one high risk of bias in the selection of the reported results (selective outcome reporting bias). Overall, two had high risk of bias.

n. One RCT - Unclear risk of bias in the randomization process (selection bias).

o. Confidence intervals indicative of appreciable benefit and harm.

p. Three NRS – Three had high risk of bias in confounding and selection of participants into the study (selection bias), one had high risk of bias in deviations from intended interventions (performance bias), and one had high risk of bias in missing outcome data (attrition bias). Overall, three had high risk of bias.

Grade Evidence table: Other biomarkers

Certainty assessment							№ of patients		Effect		Certainty	Importance
№ of studies	Study design	Risk of bias	Inconsistency	Indirectness	Imprecision	Other considerations	Other biomarkers	control	Relative (95% CI)	Absolute (95% CI)		
**Serum superoxide dismutase (SOD)**												
2	RCTs	very serious[a]	not serious	not serious	serious[b]	none	44	48	-	MD **0.42 U/mL higher** (0.71 lower to 1.55 higher)	⊕◯◯◯ Very low	CRITICAL
**Erythrocyte superoxide dismutase (SOD)**												
3	RCTs / NRS	very serious[c]	serious[d]	not serious	serious[b]	none	276	149	-	SMD **0.3 SD higher** (0.26 lower to 0.85 higher)	⊕◯◯◯ Very low	CRITICAL
**Fasting glucose: All studies**												
5	RCTs / NRS	very serious[e]	serious[f]	not serious	serious[b]	none	113	120	-	MD **0.68 mg/dL lower** (4.56 lower to 3.19 higher)	⊕◯◯◯ Very low	CRITICAL
**Fasting glucose by dose: Supplementation 16 to 25 mg/d Zn**												
1	NRS	very serious[g]	not serious	not serious	serious[h]	none	7	7	-	MD **1.4 mg/dL lower** (12.87 lower to 10.07 higher)	⊕◯◯◯ Very low	IMPORTANT
**Fasting glucose by dose: Supplementation 26 to 50 mg/d Zn**												
4	RCTs	very serious[i]	serious[j]	not serious	serious[b]	none	106	113	-	MD **0.62 mg/dL lower** (4.98 lower to 3.74 higher)	⊕◯◯◯ Very low	IMPORTANT

Certainty assessment							№ of patients		Effect		Certainty	Importance
№ of studies	Study design	Risk of bias	Inconsistency	Indirectness	Imprecision	Other considerations	Other biomarkers	control	Relative (95% CI)	Absolute (95% CI)		
**Fasting insulin: All studies**												
3	RCTs / NRS	very serious[k]	not serious	not serious	serious[b]	none	53	53	-	MD **2.02 µIU/ml lower** (3.01 lower to 1.02 lower)	⊕◯◯◯ Very low	CRITICAL
**Fasting Insulin by sex: Males**												
1	NRS	very serious[l]	not serious	not serious	serious[h]	none	7	7	-	MD **2.1 µIU/ml lower** (6.25 lower to 2.05 higher)	⊕◯◯◯ Very low	IMPORTANT
**Fasting Insulin by sex: Females**												
2	RCTs	very serious[m]	serious[n]	not serious	serious[h]	none	46	46	-	MD **1.65 µIU/ml lower** (3.63 lower to 0.33 higher)	⊕◯◯◯ Very low	IMPORTANT
**Hair zinc**												
4	RCTs	very serious[o]	serious[j]	not serious	serious[b]	none	191	190	-	MD **7.52 µg/g higher** (0.94 lower to 15.99 higher)	⊕◯◯◯ Very low	CRITICAL
**Nail zinc**												
2	RCTs	very serious[p]	serious[q]	not serious	serious[h]	none	126	102	-	MD **10.47 µg/g higher** (12.09 lower to 33.03 higher)	⊕◯◯◯ Very low	CRITICAL

Certainty assessment							№ of patients		Effect		Certainty	Importance
№ of studies	Study design	Risk of bias	Inconsistency	Indirectness	Imprecision	Other considerations	Other biomarkers	control	Relative (95% CI)	Absolute (95% CI)		
**Brain derived neurotrophic factor (BDNF)**												
2	RCTs	serious[r]	serious[s]	not serious	serious[b]	none	49	54	-	MD **2.79 ng/mL higher** (3.23 lower to 8.8 higher)	⊕◯◯◯ Very low	CRITICAL
**Insulin-like growth factor 1 (IGF-1)**												
2	RCT / NRS	very serious[t]	serious[u]	not serious	serious[b]	none	104	101	-	MD **3.15 µg/L higher** (49.6 lower to 55.91 higher)	⊕◯◯◯ Very low	CRITICAL
**Interleukin 6 (IL-6)**												
2	RCTs	serious[v]	not serious	not serious	serious[h]	none	40	40	-	MD **0.64 pg/mL lower** (1.18 lower to 0.1 lower)	⊕⊕◯◯ Low	CRITICAL
**Insulin Resistance (HOMA-IR)**												
3	RCTs / NRS	very serious[w]	serious[x]	not serious	serious[h]	none	53	53	-	MD **0.08 lower** (0.69 lower to 0.53 higher)	⊕◯◯◯ Very low	CRITICAL
**Total Antioxidant Capacity (TAC)**												
3	RCTs / NRS	very serious[y]	serious[q]	not serious	serious[z]	none	62	65	-	MD **116.96 µmol/L higher** (25.46 higher to 208.45 higher)	⊕◯◯◯ Very low	CRITICAL

Certainty assessment							№ of patients		Effect		Certainty	Importance
№ of studies	Study design	Risk of bias	Inconsistency	Indirectness	Imprecision	Other considerations	Other biomarkers	control	Relative (95% CI)	Absolute (95% CI)		
**Exchangeable Zinc Pool (EZP)**												
2	RCTs / NRS	serious[ab]	not serious	not serious	serious[h]	none	59	59	-	MD **14.44 mg higher** (9.44 higher to 19.44 higher)	⊕⊕◯◯ Low	CRITICAL

CI: confidence interval; **MD:** mean difference; **SMD:** standardized mean difference; **RCT**: randomized control trial; **NRS**: non-randomized studies

Explanations

a. Two RCTs included in the analysis. Two had unclear risk of bias in randomization process (selection bias), one had high risk of bias in deviations from intended interventions (performance bias), one had high risk of bias in missing outcome data (attrition bias), and one had unclear risk of bias in the selection of the reported results (selective outcome reporting bias). Overall, one had high risk of bias and one had unclear risk of bias.

b. Small number of events, wide confidence intervals including appreciable benefit and harm.

c. Three papers included in the analysis, two RCTs and one NRS. RCTs – Two had unclear risk of bias in the randomization process (selection bias), and one had high risk of bias in the selection of the reported results (selective outcome reporting bias). NRS - Unclear risk of bias in confounding and high risk of bias in selection of participants into the study (selection bias). Overall, two had high risk of bias and one had unclear risk of bias.

d. Wide difference in point estimates, I^2 > 80%.

e. Five papers included in the analysis, Four RCTs and one NRS. RCTs – Four had unclear risk of bias in the randomization process (selection bias), one had high risk of bias in deviations from intended interventions (performance bias), and two had high risk of bias in missing outcome data (attrition bias). NRS - High risk of bias in confounding and selection of participants into the study (selection bias). Overall, three had high risk of bias and two had unclear risk of bias.

f. Wide difference in point estimates, considerable heterogeneity, I^2 > 60%.

g. One NRS - High risk of bias in in confounding and selection of participants into the study (selection bias).

h. Wide confidence interval including appreciable benefit and harm.

i. Four RCTs included in the analysis. Four had unclear risk of bias in the randomization process (selection bias), one had high risk of bias in deviations from intended interventions (performance bias) and two had high risk of bias in missing outcome data (attrition bias). Overall, two had high risk of bias and two had unclear risk of bias.

j. Wide difference in point estimates, serious heterogeneity, $I^2 > 70\%$.

k. Three papers included in the analysis, two RCTs and one NRS. RCTs – Two had unclear risk of bias in the randomization process (selection bias), one had high risk of bias in deviations from intended interventions (performance bias), and one had high risk of bias in missing outcome data (attrition bias). NRS - High risk of bias in confounding and selection of participants into the study (selection bias). Overall, two had high risk of bias and one had unclear risk of bias.

l. One NRS - High risk of bias in in confounding and selection of participants into the study (selection bias).

m. Two RCTs included in the analysis. Two had unclear risk of bias in the randomization process (selection bias), one had high risk of bias in deviations from intended interventions (performance bias), and one had high risk of bias in missing outcome data (attrition bias). Overall, one had high risk of bias and one had unclear risk of bias.

n. $I^2 > 35\%$.

o. Four RCTs included in the analysis. Three had unclear risk of bias in the randomization process (selection bias), one had unclear risk of bias in deviations from intended interventions (performance bias), one had high risk of bias in missing outcome data (attrition bias). Overall, one had high risk of bias and two had unclear risk of bias.

p. Two RCTs included in the analysis. Two had unclear risk of bias in the randomization process (selection bias), one had unclear risk of bias in deviations from intended interventions (performance bias), one had high risk of bias in missing outcome data (attrition bias). Overall, one had high risk of bias and one had unclear risk of bias.

q. Wide difference in point estimates, considerable heterogeneity, $I^2 > 80\%$.

r. Two RCTs included in the analysis. One had unclear risk of bias in the randomization process (selection bias).

s. Wide difference in point estimates, serious heterogeneity, $I^2 > 85\%$.

t. Two studies included in the analysis, one RCT and NRS. RCT at low risk. NRS – high risk of bias in confounding and selection of participants into the study (selection bias), high risk of bias in missing outcome data (attrition bias), and high risk of bias in measurement of the outcome (detection bias). Overall, one study at high risk of bias.

u. Wide difference in point estimates, $I^2 > 35\%$.

v. Two RCTs included in the analysis. One had unclear risk of bias in the randomization process (selection bias), one had high risk of bias in deviations from intended interventions (performance bias), and one had high risk of bias in missing outcome data (attrition bias). Overall, one had high risk of bias.

w. Three papers included in the analysis, two RCTs and one NRS. RCTs – Two had unclear risk of bias in the randomization process (selection bias), one had high risk of bias in deviations from intended interventions (performance bias), and one had high risk of bias in missing outcome data (attrition bias). NRS - High risk of bias in confounding and selection of participants into the study (selection bias). Overall, two had high risk of bias and one had unclear risk of bias.

x. Serious heterogeneity, $I^2 > 75\%$.

y. Three papers included in the analysis, two RCTs and one NRS. RCTs - Two had unclear risk of bias in the randomization process (selection bias). NRS – Unclear risk of bias in confounding and high risk of bias in selection of participants into the study (selection bias). Overall, one had high risk of bias and two had unclear risk of bias. Confidence intervals including appreciable benefit and harm.

z. Confidence intervals including appreciable benefit and harm.

aa. One RCTs included in the analysis. Unclear risk of bias in the randomization process (selection bias).

ab. Two studies included in the analysis, one RCT and one NRS. NRS - High risk of bias in confounding, high risk of bias in deviations from intended interventions (performance bias), high risk of bias in missing outcome data (attrition bias). Overall, one had high risk of bias.
