# Supplementary material for: Methods of Assessment of Zinc Status in Humans: An Updated Review and Meta-analysis
Source: Nutr Rev. 2024 Jun 25;83(3):e778–800. doi: 10.1093/nutrit/nuae072 (PMC11819495; doi:10.1093/nutrit/nuae072)
Supplement: nuae072_Supplementary_Data [file nuae072_supplementary_data.zip › nuae072_Supplementary_Data/Supplemental Information 3.pdf]

Supplemental Information 3. Summary of the results of the potential biomarkers where meta-analysis could not be performed.

Note: Reference numbers refer to the reference list in the review

**Biomarkers**	**Outcomes**	**Conclusions**	**Reference**
Very low-density lipoprotein (VLDL)	Although zinc supplementation for 10 weeks to women with IUGR had beneficial effects on FPG, insulin, HOMA-IR, QUICKI, hs-CRP, TAC, and MDA status; it did not have any effect on the Pulsatility Index and other metabolic profiles including VLDL (Placebo: baseline, 33.9±6.9 mg/dL and endline 33.8 ± 11.2 mg/dL; intervention: 34.4±14.5 mg/dL, endline: 33.3±9.0 mg/dL)	No significant effect of zinc supplementation on VLDL.	[94]
AA:DGLA ratio (FADS1 activity)	In response to two weeks of zinc supplementation, the PZCs were 18% higher in the group consuming zinc before breakfast (ZWB) compared with the group that consumed supplement with the breakfasts (ZBB) (105±5.88 µg/dL compared with 88.7±2.36 µg/dL, p = < 0.05) but FADS1 activity indices were 15% higher in the ZWB than the ZBB participants (ZWB: 6.45 (5.84, 7.13); ZBB: 5.57 (5.05, 6.14); p < 0.05).	Lack of congruence between the effects of zinc supplements on PZC and EFA metabolism (FADS1) in response to whether zinc supplement is taken with or without food.	[92]
ARA, arachidonic acid	Controlling for baseline values, the ARA concentrations were 13.7% higher in the ZWB participants (baseline: 0.825 (0.753, 0.896) mM; end line 0.855 (0.784, 0.926) mM, p = 0.042) as compared with ZBB (baseline line: 0.829 (0.760, 0.897) mM; end line: 0.752 (0.683, 0.821) mM) while PZCs levels increased significantly in the ZBB group (baseline: 87.6±2.31 µg/dL; end line: 105±5.88, p = < 0.05) but not in ZWB group (Baseline: 84.8±2.34 µg/dL, Endline: 88.7±2.36 µg/dL).	Lack of congruence between the effects of zinc supplements on PZC and ARA in response to whether a zinc supplement is taken with or without food.	[92]

	No changes were observed in plasma ARA concentrations during the zinc depletion (baseline: 141.4±13.7 µg/mL, end of depletion: 138±6 14.5 µg/mL) or repletion periods (end of depletion: 138.0± 14.5 µg/mL; end of repletion 139.3±11.7 µg/mL)	Arachidonic acid levels in plasma were unaffected by changes in the dietary zinc intake	110
DNA fragmentation (Comet Assay)	Dietary zinc depletion was associated with increased DNA strand breaks in peripheral blood cells (57% increase in average tail moment from baseline to end of depletion; P= 0.05), while these changes were ameliorated by zinc repletion (decrease in average tail moment by 39.9% between the end of depletion and end of repletion; P< 0.01).	Changes in dietary zinc intake affected DNA single strand breaks. Zinc appears to be a critical factor for maintaining DNA integrity.	110
	There were no significant differences in the calculated tail moment between baseline and endpoint samples of the placebo group (p = 0.51) while the 17 days of 20 mg zinc supplementation reduced the comet tail moment (baseline: 39.7 ± 2.7 %xµ; Endline: to 30.0 ± 1.8 %xµ; p<0.01)	comet assay was sufficiently sensitive to detect changes in zinc status as a result of supplementation despite no significant changes in plasma zinc.	81
Cervicovaginal lavage (CVL) zinc level	Daily oral supplementation for two weeks had no significant impact on the CVL zinc level in either pre- (baseline: 0.009 ± 0.01 mg/L; endline: 0.004 ± 0.003 mg/L) or postmenopausal women (baseline: 0.004± 0.006 mg/L; endline: 0.003 ± 0.002 mg/L) regardless of a significant increase seen for PZC in both the groups (pre-menopausal women, baseline: 0.88 ± 0.17 mg/L; endline: 1.06 ± 0.23 mg/L, p < 0.01 and postmenopausal women, Baseline: 0.83 ± 0.24 mg/L; Endline 0.96 ± 0.33 mg/L, p < 0.01).	Daily Zinc supplementation had no significant impact on CVL zinc level.	116
DGLA, dihomo-γ - linolenic acid	No change in response to supplementation was observed for DGLA either among those consuming zinc supplements before breakfast (Baseline: 0.147 (0.128, 0.169) mM; endline: 0.146 (0.128, 0.166) mM) or those consuming with breakfast (Baseline: 0.142 (0.128, 0.157) mM; endline: 0.149 (0.130, 0.170) mM).	Daily zinc supplementation either with or without food did not affect DGLA concentrations.	92
DGLA:GLA molar ratio (ELOVL5)	No change in response to supplementation was observed for ELOVL5 activity either in the group consuming zinc supplements before breakfast (Baseline: 4.65 (3.99, 5.43); end line: 4.94 (4.22, 5.79)) or those consuming it with breakfast (Baseline: 4.70 (3.98, 5.54); end line: 4.02 (3.41, 4.73)).	Daily zinc supplementation either with or without food did not affect ELOVL5 activity.	92

DGLA/LA molar ratio	There was no significant difference in the DGLA/LA molar ratio between the ZBB and ZWB group either at baseline (ZBB: 0.109 (0.095, 0.126); ZWB: 0.111 (0.098, 0.126)) or after the supplementation (ZBB: 0.119 (0.107, 0.134); 0.113 (0.100, 0.127))	Supplementing with zinc on a daily basis, whether taken with or without meals, had no impact on the metabolism of essential fatty acids (DGLA/LA molar ratio)	92
GLA, γ -linolenic acid	Consuming zinc supplements before breakfast (ZBB, baseline: 31.7 (25.7, 39.0) nM; endline: 29.6 (25.1, 35.0) nM) had no significant effect on plasma GLA as compared to taking it along with the breakfast (ZWB, baseline: 30.2 (25.4, 35.9) nM; endline: 36.8 (31.0, 43.7) nM).	Daily zinc supplementation, whether taken with or without food, did not alter GLA	92
GLA:LA (FADS2 activity)	There was no significant difference in GLA: LA molar ratio between the ZBB and ZWB group either at baseline (ZBB: 0.025 (0.021, 0.29); ZWB: 0.024(0.020, 0.028)) or at the end of supplementation (ZBB: 0.024 (0.021, 0.028); 0.028(0.024, 0.033))	Daily Zinc supplementation either with or without food did not impact FADS2 activity.	92
LA, linoleic acid	No change in response to supplementation was observed for LA either among those consuming zinc supplements before breakfast (Baseline: 1.35 (1.26, 1.44) mM; end line:1.23 (1.13, 1.33) or those consuming with breakfast (Baseline:1.28 (1.21, 1.36) mM; end line: 1.31 (1.21, 1.42) mM).	Daily zinc supplementation either with or without food did not affect LA.	92
erythrocyte CCS:SOD1 ratio	Zinc supplementation of 5–15 mg/d for 4 months did not alter erythrocytes CCS: SOD1 ratio (baseline for all the groups: 1.00 ± 0.00; endline: placebo= -0.05 ± 0.07, 5mg/d = 0.02± 0.07, 10mg/d= 0.08± 0.07, 15 mg/d=-0.02± 0.08)	zinc supplementation did not affect erythrocyte CCS: SOD1 ratio	49
Erythrocyte osmotic fragility (%) (EOF)	The decrease in erythrocyte osmotic fragility was significantly higher ($p < 0.010$) in the zinc supplementation group (baseline 28± 18; endline: 15 ± 13) as compared to the placebo (baseline:29±12; endline: 24± 16).	Zinc supplementation decreased the erythrocyte osmotic fragility.	66
fecal sIgA	Changes in fecal sIgA levels of the placebo, probiotic, zinc, and combination of probiotic and zinc groups in the subjects, after 90 days were 13.58 ± 2.26, 30.33 ± 3.32, 20.5 ± 1.73, and 27.55 ± 2.28 µg/g feces, respectively. The changes in fecal sIgA levels in the subjects after 90 days of zinc supplementation (without probiotics) were statistically not different from placebo, probiotic alone, or a combination of zinc and placebo.	Zinc supplementation alone did not alter the fecal sIgA levels	114

Gene expression MT1	There was no difference between the baseline and endpoint for MT1 mRNA abundance in either the Placebo or zinc-supplemented group.	Zinc supplementation did not change MT1 mRNA expression.	[51]
Gene expression Zip 3*	For both, the placebo and zinc supplementation groups, there was no significant change in the abundance of the zinc transporter Zip3 mRNA from baseline to endpoint of the study.	Zinc supplementation did not alter zip 3 mRNA abundance.	[51]
Gene expression Zip 4*	Baseline mRNA abundance for zinc transporters did not differ significantly between the placebo and zinc groups. However, after 23 days of supplementation, ZIP4 mRNA abundance decreased significantly ($p = 0.036$) in the zinc group, but not in the placebo group.	Zip4 mRNA was responsive to zinc supplementation and requires further investigation	[51]
Gene expression Zip 8*	Analysis of baseline and endpoint samples showed that the Zinc transporter mRNA levels remained unaffected in the Placebo group but ZIP8 mRNA significantly decreased in response to zinc supplementation ($p = 0.038$).	ZIP8 mRNA displayed responsiveness to zinc supplementation, warranting further investigation	[51]
Gene expression ZnT1*	There was no difference between the baseline and endpoint in zinc transporter, ZnT1, and mRNA abundance in either the placebo or zinc-supplemented group.	Zinc supplementation did not change ZnT1 mRNA abundance.	[51]
	ZnT1 in the zinc group increased significantly after 8 weeks of zinc supplementation. The relative change of ZnT1 gene expression after zinc supplementation in the zinc group was 1.3 times higher than the changes in the placebo group ($p<0.01$)	Zinc supplementation led to an increase in the expression of the zinc transporter ZnT1	[97]
HbA1c	The baseline-adjusted mean group difference between the zinc supplement group and placebo for HbA1c −0.06 (−0.19, 0.06) was statistically non-significant (Zinc supplement group, Baseline: 5.87±0.19; endline: 5.84±0.29 Vs. Placebo, baseline: 5.90± 0.20; endline: 5.96±0.30). There were no overall differences in HbA1c across the three follow-up time points namely 1, 6, and 12 months.	Zinc supplementation to pre-diabetic individuals had no effect on HbA1c.	[41]
IGFBP-3	Two months of zinc supplementation to children with Zn deficiency and growth retardation but without systemic disease did not have any significant effect on IGFBP-3 (Baseline: 2773.5±797. 1 ng/mL; Endline: 2696.4± 818.3 ng/mL)	Zinc supplementation did not change IGFBP-3	[53]
Kinetics Parameters of venous zinc	All pharmacokinetic parameters studied in serum zinc (SZn), biological half-life of serum zinc ($T_{1/2}$), Elimination constant of serum zinc (K_{el}),	Zinc kinetics showed a positive response to	[86]

tolerance test (VZnTT)	and the total body clearance of zinc (CZn) were significantly different before and after zinc supplementation (SZn: baseline 102 ± 10.1 μg/dL Vs.endline 122 ± 19.5 μg/dL, p<0.0001; $T_{1/2}$: baseline 2.73 ± 0.597 hrs Vs. endline 2.39± 0.261 hrs, p = 0.0006; K_{el} : baseline 0.265 ± 0.051 kel/h Vs. endline 0.294 ± 0.033 kel/h, p = 0.0004; CZn: baseline: 5.52 ± 1.20 mL/kg/h Vs. endline 6.22 ± 1.05 mL/kg/h p= 0.0002, except the distribution volume (baseline: 0.0209 ± 0.0028; Endline: 0.0211 ± 0.0022, p = 0.6432).	supplementation and even maybe a sensitive parameter in children without a deficiency of this mineral.	
Plasma conjugated dienes (nmol g-1 total lipid) (PCD)	The increase in the plasma conjugated dienes was significantly lower (p < 0.010) in the zinc supplementation group (baseline 1.1 ± 0.2 nmol/g total lipid; endline 1.3 ± 0.2 nmol/g total lipid) as compared to the placebo (Baseline1.1 ± 0.3 nmol/g total lipid; Endline 1.7 ± 0.3 nmol/g total lipid).	Zinc supplementation led to lower levels of plasma conjugated dienes compared with the control group.	[66]
Plasma Zn: Cu ratio (Pl Zn:Cu)	Plasma Zn/Cu ratio was significantly increased at the end of zinc supplementation (day 30) and on discontinuing supplementation (and instead continuing placebo) for the next 30 days (0.7±0.1 at day 30 and 0.8±0.2 at day 60, respectively), compared to baseline (0.6±0.2) (p=0.04).	Plasma Zn/Cu ratio responded to zinc supplementation but not to its discontinuation.	[91]
Renal zinc clearance (Note this is not total body zinc clearance)	The increase in Renal zinc clearance in response to supplementation was not significant (before 0.45 ml/kg/h; after 0.51ml/kg/h; p=0.0732).	Renal clearance was non-responsive to zinc supplementation	[120]
secretory phospholipase (sPLA)	Six months of zinc supplementation did not change the plasma sPLA (Baseline 73.3 ± 34.6 U/mL; Endline: 70.0 ±32.2 U/m; p=0.314) while a significant increase was reported for the placebo group (Baseline:76.0 ± 25.8; Endline: 100.6 ± 28.8; p= 0.001)	Zinc supplementation led to a reduction in sPLA after 6 months compared with placebo in elderly participants.	[45]
Serum retinol	There was a significant increase in the serum retinol level in the group receiving zinc plus vitamin A supplementation as compared to the group receiving placebo with vitamin A (p < 0.03) for six days a week for a total of six months.	Zinc supplementation led to increase in Serum retinol level	[38]
Total body zinc clearance (CZn)	The total body clearance of zinc (CZn) increased significantly after zinc supplementation (CZn: baseline: 5.52 ± 1.20 mL/kg/h Vs. endline 6.22 ± 1.05 mL/kg/h p= 0.0002).	Total body Zinc clearance responded to zinc supplementation	[86]

	Total body zinc clearance increased significantly in response to supplementation (Median, before 5.20 ml/kg/h; after 5.93; p= 0.002 ml/kg/h). CZn was more effective (P= 0.0002) more effective than systemic clearance (P= 0.6028) and renal clearance (P= 0.0732) in detecting small variations in body zinc status.	Zinc supplementation led to increase in total body zinc clearance	[120]
Total glutathione (GSH)	The change in GSH levels was not significantly different (p=0.71) between the placebo (Baseline: 708.3 ± 138.7 mmol/L; Endline 700.7 ± 136.8 mmol/L) and zinc-supplemented group (baseline: 671.2 ± 144.8 mmol/L; Endline: 703.6 ± 171.4 mmol/L)	Zinc Supplementation did not have an impact on GSH	[94]
ZnT2	The change in ZnT2 gene expression after zinc supplementation in the zinc group was similar to change in the placebo group.	Zinc supplementation did not alter ZnT2 mRNA expression	[97]
ZnT5	ZnT5 in the zinc group increased significantly after 8 weeks of zinc supplementation. The relative change of ZnT5 gene expression after zinc supplementation in the zinc group was 1.2 times higher than the changes in the placebo group (P<0.01)	Zinc supplementation led to an increase in the expression of the zinc transporter ZnT5	[97]
ZnT6	Although the relative change of ZnT6 gene expression after zinc supplementation in the zinc group was 1.3 times higher than the changes in the placebo group, it was not significantly different.	Zinc supplementation did not alter ZnT6 mRNA expression	[97]
ZnT9	While the zinc group exhibited a 1.2-fold increase in ZnT6 gene expression following zinc supplementation compared to the placebo group, this difference did not reach statistical significance.	Zinc Supplementation did not change ZnT9 mRNA expression	[97]
